# The bear in Eurasian plant names: motivations and models

**DOI:** 10.1186/s13002-016-0132-9

**Published:** 2017-02-21

**Authors:** Valeria Kolosova, Ingvar Svanberg, Raivo Kalle, Lisa Strecker, Ayşe Mine Gençler Özkan, Andrea Pieroni, Kevin Cianfaglione, Zsolt Molnár, Nora Papp, Łukasz Łuczaj, Dessislava Dimitrova, Daiva Šeškauskaitė, Jonathan Roper, Avni Hajdari, Renata Sõukand

**Affiliations:** 1 Institute for Linguistic Studies, Russian Academy of Sciences, Tuchkov pereulok 9, Saint-Petersburg, 199053 Russia; 2 Uppsala Centre for Russian and Eurasian Studies, Uppsala University, Box 514, SE-751 20 Uppsala, Sweden; 3 Estonian Literary Museum, Vanemuise 42, Tartu, 51003 Estonia; 4 Department of Anthropology, University of Alaska Fairbanks, Fairbanks, AK 99775-7720 USA; 5 Department of Pharmaceutical Botany, Faculty of Pharmacy, Ankara University, 06100 Tandoğan, Ankara Turkey; 6 University of Gastronomic Sciences, Piazza Vittorio Emanuele 9, I-12042 Pollenzo/Bra, Italy; 7 School of Biosciences and Veterinary Medicine, University of Camerino, Via Pontoni, 5 62032 Camerino, (MC) Italy; 8 MTA Centre for Ecological Research, Institute of Ecology and Botany, Alkotmány u. 2-4, H-2163 Vácrátót, Hungary; 9 Department of Pharmacognosy, University of Pécs, Rókus 2, 7624 Pécs, Hungary; 10 Department of Botany; Institute of Applied Biotechnology and Basic Sciences, University of Rzeszów, Werynia 502, 36-100 Kolbuszowa, Poland; 11 Institute of Biodiversity and Ecosystem Research, Bulgarian Academy of Sciences, Acad. Georgi Bonchev Str., bl. 23, Sofia, 1113 Bulgaria; 12 Faculty of Humanities, Vilnius University Kaunas, Sargeliai, LT 60433 Žaiginio paštas Lithuania; 13 Department of Estonian and Comparative Folklore, Tartu University, Ülikooli 16, 50090 Tartu, Estonia; 14 Department of Biology, University of Prishtina, St. Mother Teresa, Prishtinë, Kosovo

**Keywords:** Ethnobotany, Ethnolinguistics, Traditional knowledge, Phytonyms, Brown bear *Ursus arctos*, Motivation, Latin calques

## Abstract

Ethnolinguistic studies are important for understanding an ethnic group’s ideas on the world, expressed in its language. Comparing corresponding aspects of such knowledge might help clarify problems of origin for certain concepts and words, e.g. whether they form common heritage, have an independent origin, are borrowings, or calques. The current study was conducted on the material in Slavonic, Baltic, Germanic, Romance, Finno-Ugrian, Turkic and Albanian languages. The bear was chosen as being a large, dangerous animal, important in traditional culture, whose name is widely reflected in folk plant names. The phytonyms for comparison were mostly obtained from dictionaries and other publications, and supplemented with data from databases, the co-authors’ field data, and archival sources (dialect and folklore materials). More than 1200 phytonym use records (combinations of a local name and a meaning) for 364 plant and fungal taxa were recorded to help find out the reasoning behind bear-nomination in various languages, as well as differences and similarities between the patterns among them. Among the most common taxa with bear-related phytonyms were *Arctostaphylos uva-ursi* (L.) Spreng., *Heracleum sphondylium* L., *Acanthus mollis* L., and *Allium ursinum* L., with Latin loan translation contributing a high proportion of the phytonyms. Some plants have many and various bear-related phytonyms, while others have only one or two bear names. Features like form and/or surface generated the richest pool of names, while such features as colour seemed to provoke rather few associations with bears. The unevenness of bear phytonyms in the chosen languages was not related to the size of the language nor the present occurence of the Brown Bear in the region. However, this may, at least to certain extent, be related to the amount of the historical ethnolinguistic research done on the selected languages.

## Background

Many plant names have an animal element as a part of them. Animal names in folk (and scientific) plant names are common in many languages in Europe, as well as outside Europe. The prefix *bear*- is very common in many languages in the Eurasian area, which reflects the bear’s importance in the folk tradition.

Phytonyms (that is plant names) with animal names as components have been discussed by many ethnobotanists, folklorists, and linguists. Already a pioneering plant name researcher T. Thiselton-Dyer [1] observed that bear was a common animal element in many plant names, giving *bear’s foot*, *bear-berry*, *bear’s bilberry*, *bear’s-garlic*, *bears-breech*, and *bear’s-wort* as examples. The word *bear* could denote the size, the coarseness, and frequently the worthlessness or spuriousness of the plant. H. Kreiter [2] gave an account of French vernacular names of plants derived from animal names. H. Marzell published a dissertation on animals in German plant names [3]. Croatian and general Slavonic material was analysed in works by N. Vajs [4] and S. Dubrovina [5], respectively. I. Hauenschield provided an important contribution with her studies on the use of animals in Turkic plant names [6]. She noted that the bear is an important animal in Turkish folklore and also has a very prominent place among Turkic plant names, generally connected with forest and mountain flora. Hungarian plant names were researched by Rácz [7], who considered dog’s, wolf’s, pig’s names among the most common ones among those with an animal element. Komi phytonyms were discussed by Rakin [8]. Most of these works analyse many plant names in detail, but somewhat selectively, and without statistics.

**Fig. 1:**
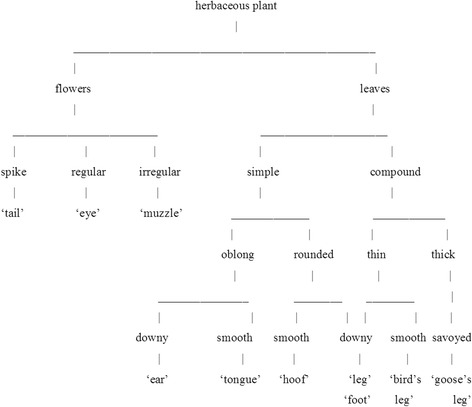
Correspondences between the appearance of plants and animal body parts based on nomination (adapted from [4])

Specific animals were discussed in numerous articles. For example, the Dutch ethnobotanist T. van Asseldonk examined the use of ‘pig’ in plant names in French, German, Dutch, and Flemish [9]; T. B. Haber – on dogs and other canines in British and American English [10]. Some authors tried to find explanations of naming in traditional culture [11]; such an approach is typical in ethnolinguistics. Still, most researchers limited themselves to comparing plants’ organs with these or those body parts of animals, as well as pointing out some of their features – that is, their properties and characteristics. So, van Asseldonk mentioned the toxicity of some ‘pig’-herbs and the fact that some of them are eaten by pigs [9]; Haber included worthless, inferior, harmful, and not cultivated plants to the list of ‘dog’ plants, adding those serving as dog’s medicine, curing dog bites, and beneficial to dogs [10]. In a work based on Slavonic plant name data, V. Kolosova [12] discerned sixteen motivations (plant features which were the reason behind the naming) for animal names, a plant’s shape, surface, colour, size, habitat, status, and a plant being animal’s food or medicine among them. N. Vajs in her article [4] attempted to set up correspondences between the appearance of plants and an animal body part forming the basis of nomination (Fig. 1).

More specifically plant names with a *bear* component were discussed in articles by Brodskij [13] on Finno-Permic and Balto-Fennic languages and by Kolosova [14] on Russian dialects. We should also mention Dahlstedt [15] who in more depth discussed many bear berry names in northern Scandinavia. He notes that *bear*- in names of berries in northern Scandinavia is probably pejorative, because it is often used to berries regarded as inedible. However, it can also refer to the colour, because unripe berries can be red or maroon.

There was often no direct relationship between the actual habitat of an animal species and the use of animal metaphors in local plant names. There were for instance names with ‘lion’, ‘monkey’ and ‘dragon’ in the Nordic languages, and ‘elephant’ and ‘lion’ in various Turkic plant names [6, 16, 17]. The Tudor naturalist Turner recorded *bearefote* (nowadays still called *bear’s foot*) in English already in 1538 for *Helleborus* [18]. No wild bears were present in the British Isles at that time. They were probably extinct already by 500 AD or somewhat later [19].

Bear-related plant names can be considered semantically transparent or in some cases semi-transparent in Berlin’s terms [20], yet we are talking about once traditional, but now highly literate societies. Therefore understanding the motivations of plant names is not easy. Plant names and the naming of plants subsume a great many aspects: synchronic, diachronic, structural, semantic/associative, systemic/variational, etymological, geographical, social, etc. There is also a question of the emotive/situational value of plant names and the handling of plant names in literary contexts. Many plant names reflected folklore motifs and mythological ideas. In addition, there were normative aspects in terms of diffusion and normalization of plant names. It is therefore not only a question of the supposed origin and motif of denomination (why was a plant X called Y?). Some names were for instance just translations of the Latin name, and especially the pre-Linnaean Latin names must be considered, cf. Turner’s *bearefote* [21].

Large charismatic animals have always attracted people’s interest, and therefore there exists a rich cultural history associated with such animals. Predators especially have exerted a special attraction in art, folklore, myths, rituals, and other cultural expressions. This applies in particular to the bear (along with wolf) that has played a central role in people’s perceptions across Eurasia. Bear worship has for instance been common all over the circumpolar area [22–26]. Some peoples even regarded bears as their ancestors [27, 28]. Vice versa, in Slavonic etiological legends bear is a human transformed into a beast as a punishment for some sin. South Slavs celebrated special “bear days” [29].

Bears are found all over the northern hemisphere [30]. Very much was written about the bear in folk perception and as a prey for hunters. Our relationship with bears seems eternal, ranging from prehistoric human relation to the cave bear right up to the bear’s place in today’s popular culture [31, 32]. Several ancient (Pliny the Elder, Claudius Aelianus), medieval (Hildegard of Bingen, Albertus Magnus, Bartholomew of England), and Renaissance authors (Olaus Magnus, Conrad Gessner) dealt with the bear in detail. The scholarly literature on the bear in human history is extensive and covers many aspects [27, 30, 33–36].

Echoes of ancient ideas on some “equality” of humans and bears have been alive in ritual practices until recently. For instance, in Älvdalen, in the Swedish province Dalecarlia, a fiancée was called ‘she-bear’, and there are many expressions connected with the rituals before a wedding when the bear metaphor is used. *Björn-grånka* is the local term for a spruce tree, *Picea abies* (L.) H. Karst, which was set upright against the door of a farm where someone had the banns of marriage published. This was done to prevent ‘the bear’ from coming ‘out of hibernation’ [37]. In Russian wedding songs the bride is called *мeдвeдицa* ‘she-bear’ [38].

As a charismatic animal, hunted, revered, and feared, the bear also has many euphemisms in Eurasia [29, 39–41]. Strictly speaking, *bear/björn/bjørn/Bär* in the Germanic languages literally mean “the brown one”, is an old euphemism [41]; Russian literary and common name for bear is also a euphemism literally meaning “honey eater”; all of them are used instead of old Indo-European name *ŗkso- (kept in Gr. *άρκτoς* and Lat. *ursus*), that has been tabooed for many centuries [42]. The Hungarian language uses a Slavonic borrowing *medve* for the same reason.

The objectives of the research were to answer the following questions:

- which plants were associated with the bear in folk plant nomenclatures?
- why certain plants were considered ‘bear plants’ (i.e. eventual links to local perceptions)?
- is there a common perception of the same taxon over larger areas?
- what are differences and similarities of the patterns among different languages?
- do plant names with ‘bear’ component always link to the animal ‘bear’?
- which word-formative models are used for creating ‘bear’-names?

## Methods and sources

**Fig. 2:**
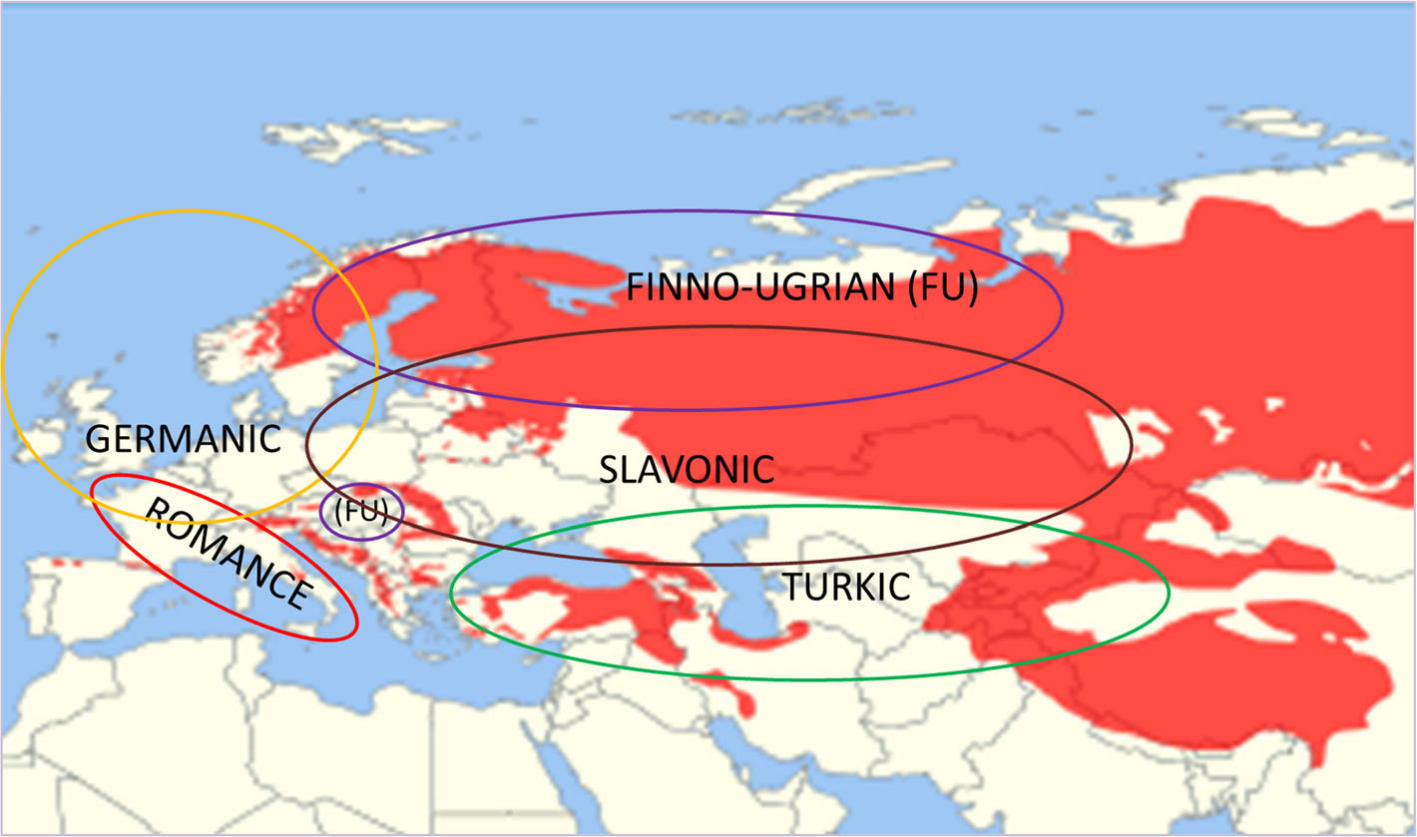
Language groups reviewed in this publication and present geographic distribution of Brown Bear (*Ursus arctos*). *Red* – distribution of Brown Bear. *Circles* indicate the regions inhabited by the speakers of the language group very roughly. Base map (species distribution map) is created by the International Union for Conservation of Nature and distributed by Wikimedia according to Attribution-Share Alike Creative Commons License (https://commons.wikimedia.org/wiki/File:Ursus_arctos_range_map.svg)

Within this work we concentrated on plant names related to the Brown Bear (*Ursus arctos* Linnaeus, 1758) historically or still today co-habitating in the areas of the distribution of selected language groups (Fig. 2). We give an overview of using the bear as a prefix in Eurasian folk plant names (sometimes it is not a prefix but just a word meaning ‘bear’ or ‘little bear’ used as a metaphorical name). The names belonging to the official nomenclature were not analysed, unless they coincided with the folk ones.

Only species names were considered. The cases of bear-nomination for individual natural objects, as, i.e., It. *faggio dell’orso* ‘bear’s beech’ for one famous big tree at Mt. Tranquillo, near Pescasseroli village, are not analysed.

Plant names containing the root *bear*- were analysed in the spoken dialects of several Eurasian languages: Slavonic (Belarusian, Bulgarian, Croatian, Czech, Macedonian, Polish, Russian, Ruthenian, Serbian, Slovak, Slovenian, Sorbian, and Ukrainian), Germanic (English, German, Danish, Norwegian and Swedish), Romance (French, Italian, Latin, Sardinian, Friulian), Finno-Ugrian (Estonian, Finnish, Hungarian, Izhorian, Livonian, Saami, and Votic), Turkic languages (Bashkir, Chuvash, Kazakh, Kirghiz, Tatar, Turkish, Uyghur, and Uzbek) and other languages (Albanian, Lithuanian). The authors also considered the cases when the plant was unidentified, but the citation from respondent explained the using of a bear-name. The inner form (literal translation) of local names is given in square brackets.

The published dictionaries, ethnolinguistic literature, folkloric and ethnographic references based on primary literature and original field investigations were considered. Field materials from Abruzzo [Cianfaglione], Transylvania [Frendl and Papp], Estonia [Kalle and Sõukand], Transylvania [Molnár, Babai], and the Alps [Pieroni] are published for the first time. Data was extracted from the sources and entered in an Excel spreadsheet. Nomenclature followed The Plant List database [43] for plants and Index Fungorum [44] for fungi. The dataset supporting the conclusions of this article was included within the article as its (Table 1). The dataset has the following structure: valid nomenclature taxon, original source taxon identification, local plant name and its literal translation into English, language and geographical area of fixation of the plant name, motivation, and the source of the information. The column containing motivations was formed on the basis of the material extracted from the sources.

**Table 1:** ᅟ

AA currently valid nomenclature	The taxon indicated in original source	Local name	Literal translation	Language	Area	Motivation	Reference
*Acanthus hirsutus* Boiss.	*Acanthus hirsutus* Boiss.	ayıeli	bear hand	Turkish	Anatolia	shape	[59]
*Acanthus mollis* L.	*Acanthus mollis*	ayïpänġäsi	bear claw	Azeri		Latin calque	[171]
*Acanthus mollis* L.	*Acanthus mollis*	ayïwtabani	bear sole	Bashkir		Latin calque	[171]
*Acanthus mollis* L.	*Acanthus mollis*	upauri	bear foot	Chuvash		Latin calque	[171]
*Acanthus mollis* L.	*Acanthus mollis* L.	medviđa stopa	bear foot	Croatian		Latin calque	[126, 137, 138]
*Acanthus mollis* L.	*Acanthus mollis* L.	medvidnja stupa	bear foot	Croatian		Latin calque	[126, 137, 138]
*Acanthus mollis* L.	*Acanthus mollis* L.	medvija stupa	bear foot	Croatian		Latin calque	[126, 137, 138]
*Acanthus mollis* L.	*Acanthus mollis* L.	medvjeđa stupa	bear foot	Croatian	no location	Latin calque	[84, 126, 137, 138]
*Acanthus mollis* L.	*Acanthus mollis* L.	medvejsko kopito	bear hoof	Croatian	no location	?	[126, 138]
*Acanthus mollis* L.	*Acanthus mollis* L.	medvedovina	bear plant	Croatian	no location	Latin calque	[126, 138, 225]
*Acanthus mollis* L.	*Acanthus mollis* L.	stopa medvidnja	bear foot	Croatian		Latin calque	[126, 138, 239]
*Acanthus mollis* L.	*Acanthus mollis* L.	stopa medvinja	bear foot	Croatian		Latin calque	[126, 138, 239]
*Acanthus mollis* L.	*Acanthus mollis* L.	stopa medvjednja	bear foot	Croatian		Latin calque	[126, 138, 239]
*Acanthus mollis* L.	*Acanthus mollis* L.	stopa medvjeđa	bear foot	Croatian		Latin calque	[126, 138, 239]
*Acanthus mollis* L.	*Acanthus mollis* L.	stopa medvjeska	bear foot	Croatian		Latin calque	[126, 138, 239]
*Acanthus mollis* L.	*Acanthus mollis* L.	medvědí pazneht	bear hoof	Czech		Latin calque	[61]
*Acanthus mollis* L.	*Acanthus mollis* L.	medvědí paznecht	bear hoof	Czech		Latin calque	[61]
*Acanthus mollis* L.	*Acanthus mollis* L.	nedvědí paznot	bear hoof	Czech		Latin calque	[61]
*Acanthus mollis* L.	*Acanthus mollis* L.	nedvězí paznot	bear hoof	Czech		Latin calque	[61]
*Acanthus mollis* L.	*Acanthus mollis* L.	vlašský nedvědí pazneht	Italian bear hoof	Czech		Latin calque	[61]
*Acanthus mollis* L.	*Acanthus mollis* L.	vlašský nedvědí paznecht	Italian bear hoof	Czech		Latin calque	[61]
*Acanthus mollis* L.	*Acanthus mollis* L.	patte d’ours	bear paw	French		Latin calque	[2]
*Acanthus mollis* L.	*Acanthus mollis* L.	branc ursine	bear paw	French		Latin calque	[2]
*Acanthus mollis* L.	*Acanthus mollis* L.	branque ursine	bear paw	French		Latin calque	[2]
*Acanthus mollis* L.	*Acanthus mollis* L.	branche ursine	bear paw	French		Latin calque	[2]
*Acanthus mollis* L.	*Acanthus mollis*	Bärenklau, echte, italienische, welsche	bear claw, true, Italian, ?	German, non specified	not specified	shape?	[65]
*Acanthus mollis* L.	*Acanthus mollis*	Bärentappe	bear paw	German, non specified	not specified	shape?	[65]
*Acanthus mollis* L.	*Acanthus mollis*	Bärentatze	bear paw	German, non specified	not specified	shape?	[65]
*Acanthus mollis* L.	*Acanthus mollis* L.	branca ursina	bear paw	Italian, central	Tuscany, central Italy	?	[74]
*Acanthus mollis* L.	*Acanthus mollis*	ayuwayaghï	bear foot	Kazakh		Latin calque	[171]
*Acanthus mollis* L.	*Acanthus mollis*	ayuwtabanï	bear sole	Kazakh		Latin calque	[171]
*Acanthus mollis* L.	*Acanthus mollis* L.	мeдвeдзoвa тaлпa	bear foot	Ruthenian	Vojvodina	Latin calque?	[125]
*Acanthus mollis* L.	*Acanthus mollis* L.	мeдвeдoвинa	bear plant	Serbo-Croat		Latin calque?	[84]
*Acanthus mollis* L.	*Acanthus mollis* L.	мeдвejcкo кoпитo	bear hoof	Serbo-Croat		Latin calque?	[84]
*Acanthus mollis* L.	*Acanthus mollis* L.	мeдвecкa cтoпa	bear foot	Serbo-Croat		Latin calque?	[84]
*Acanthus mollis* L.	*Acanthus mollis* L.	мeдвидњa cтупa	bear foot	Serbo-Croat		Latin calque?	[84]
*Acanthus mollis* L.	*Acanthus mollis* L.	мeдвиja cтупa	bear foot	Serbo-Croat		Latin calque?	[84]
*Acanthus mollis* L.	*Acanthus mollis*	medvednik	bear plant	Slovenian		Latin calque?	[84]
*Acanthus mollis* L.	*Acanthus mollis*	medvenik	bear plant	Slovenian		Latin calque?	[84]
*Acanthus mollis* L.	*Acanthus mollis*	barjaca stopa	bear foot	Sorbian		Latin calque	[186]
*Acanthus mollis* L.	*Acanthus mollis*	ayuajaghï	bear foot	Tatar		Latin calque	[171]
*Acanthus mollis* L.	*Acanthus mollis*	ayutabanï	bear sole	Tatar		Latin calque	[171]
*Acanthus mollis* L.	*Acanthus mollis*	ayıpençesi	bear claw	Turkish		shape	[171]
*Acanthus mollis* L.	*Acanthus mollis*	ayïpenġesi	bear claw	Turkmen		Latin calque	[171]
*Acanthus mollis* L.	*Acanthus mollis*	barica	she-bear	Upper-Sorbian		Latin calque?	[63]
*Acanthus mollis* L.	*Acanthus mollis*	ayïqtåwån	bear sole	Uzbek		Latin calque	[171]
*Acanthus spinosus* L.	*Acanthus spinosus* L.	branca ursina salvatica	wild bear branch	Italian, central	Tuscany, central Italy	?	[74]
*Acanthus spinosus* L.	*Acanthus spinosus* L.	barica	she-bear	Upper-Sorbian		Latin calque	[121]
*Acanthus* spp.	*Acanthus* spp.	medvedska taca	bear paw	Croatian		Latin calque	[228]
*Acanthus* spp.	*Acanthus* (various species, including spinosus and mollis)	bear's breech	bear breech	Eng. General	England	surface	[46]
*Acanthus* spp.	*Acanthus* L.	ayıpençesi	bear paw	Turkish	Anatolia	shape	[131]
*Acanthus* spp.	*Acanthus*	barnica	bear plant	Upper-Sorbian		Latin calque?	[63]
*Achillea millefolium* L.	*Achillea millefolium* L.	karhunkukka	bear flower	Finnish	Perho, Sievi	surface	[82]
*Achillea millefolium* L.	*Achillea millefolium* L.	karhunruoho	bear grass	Finnish	Perho	surface	[82]
*Achillea nobilis* L.	*Achillea nobilis* L.	ayıdanası	bear calf	Turkish	W Anatolia	folklore	[145]
*Aconitum napellus* L.	*Aconitum napellus*	bear's foot	bear foot	Eng. Midland	Nottingham	shape	[45]
*Aconitum napellus* L.	*Aconitum napellus* L.	karupesa	bear nest	Estonian	Mus	size	[58]
*Aconitum* sp.	*Aconitum* sp.	ayïwtabani	bear sole	Bashkir		shape	[171]
*Aconitum* sp.	*Aconitum* sp.	ayïqtåwån	bear sole	Uzbek		shape?	[171]
*Acorus calamus* L.	*Acorus* cal.	Bärenmutter-wurzel	bear mother root	German, non specified	not specified	folk etymology?	[65]
*Actaea rubra* (Aiton) Willd.	*Actaea rubra* (Aiton) Willd.	karhunmarja	bear berry	Finnish	Sort, Konn	dangerousness	[82]
*Actaea spicata* L.	*Actaea spicata* L.	мeдвeжьи ягoды	bear berries	Central Russian	Nizhni Novgorod	inedibility?	[57]
*Actaea spicata* L.	*Actaea spicata* L.	meškauogė	bear berries	Lithuanian	Lithuania	dangerousness, bear food	[87, 191]
*Actaea spicata* L.	*Actaea spicata* L.	мeдвeжья тpaвa	bear grass	Northern Russian	Olonets	dangerousness (toxic)?	[57]
*Actaea spicata* L.	*Actaea spicata* L.	bjønnbær	bear berry	Norwegian	Northern Odal, Vodal	?	[80]
*Actaea spicata* L.	*Actaea spicata* L.	мeдвeжья ягoдa	bear berry	Russian	Middle Ob region	dangerousness (toxic)	[101]
*Actaea spicata* L.	*Actaea spicata*	björnbär	bear berry	Swedish	Österbotten, Nyland	status-	[155]
*Aegopodium podagraria* L.	*Aegopodium podagraria*	medvedova taca	bear paw	Slovenian		?	[84, 144]
*Agasyllis latifolia* Boiss.	*Siler latifolium* L.	medvedovka	bear plant	Slovenian		?	[144]
*Ajuga reptans* L.	*Ajuga reptans* L.	мeчa cтъпкa	bear step	Bulgarian		shape, surface	[86]
*Alchemilla xanthochlora* Rothm.	*Alchemilla vulgaris* L.	bear's foot	bear foot	Eng. Southern, Northern	Hampshire, Northumberland	shape	[45]
*Alchemilla xanthochlora* Rothm.	*Alchemilla* vulg.	Barendaumen	bear thumb	German, non specified	not specified	shape?, surface?	[65]
*Allium rotundum* L.	*Allium rotundum* L.	ayı sarmısağı	bear garlic	Turkish	Kırklareli (European part of Turkey)	wild	[227]
*Allium ursinum* L.	*Allium ursinum* L.	Hudhër arushe, Hudhrr ariu	bear garlic	Albanian	Albania	bear food	[210, 211]
*Allium ursinum* L.	*Allium ursinum*	ayï soganï	bear onion	Azeri		Latin calque	[171]
*Allium ursinum* L.	*Allium ursinum*	ayïu huganï	bear onion	Bashkir		Latin calque	[171]
*Allium ursinum* L.	*Allium ursinum* L.	мeчи лук	bear onion	Bulgarian	Kyustendil area	Latin calque	[86]
*Allium ursinum* L.	*Allium ursinum*	bjørneløg	bear onion	Danish	Denmark	Latin calque	[187]
*Allium ursinum* L.	*Allium ursinum* L.	karusibul	bear onion	Estonian	Hää	Latin calque	[58]
*Allium ursinum* L.	*Allium ursinum *L.	karulauk	bear leek	Estonian		Latin calque	([58, 237, 238]
*Allium ursinum* L.	*Allium* urs.	Bärenknoblauch	bear garlic	German, non specified	not specified	bear food	[65]
*Allium ursinum* L.	*Allium* urs.	Bärlauch	bear leek	German, non specified	not specified	bear food	[65]
*Allium ursinum* L.	*Allium * *ursinum*	medvefokhagyma	bear garlic	Hungarian	Egerszék (Transylvania)	wild	Frendl and Papp unpublished
*Allium ursinum* L.	*Allium ursinum*	medvesósdi	bear’s sorrel	Hungarian	Egerszék (Transylvania)	wild	Frendl and Papp unpublished
*Allium ursinum* L.	*Allium ursinum* L.	ajë dell'orse; aju dell'orso; ajo dell'ors	bear garlic	Italian, central	Gran Sasso d'Italia and Laga Mt., Abruzzo and little portion of eastern Latium, central Italy	wild, status-	Cianfaglione unpublished
*Allium ursinum* L.	*Allium ursinum* L.	ajë de l'urs	bear garlic	Italian, central	Valle Peligna and Alto Sangro, Abruzzo, central Italy	wild, status-	Cianfaglione unpublished
*Allium ursinum* L.	*Allium ursinum* L.	ajë dë jursë	bear garlic	Italian, southern	Valle Subequana, mid Valle dell'Aterno, Altopiano delle Rocche (L'Aquila), Abruzzo, central Italy	wild, status-	Cianfaglione unpublished
*Allium ursinum* L.	*Allium ursinum*	ayuw xogon	bear onion	Kirghiz		Latin calque	[171]
*Allium ursinum* L.	*Allium ursinum*	meškinis česnakas	bear garlic	Lithuanian	Lithuania	Latin calque, bear food	[87]
*Allium ursinum* L.	*Allium ursinum* L.	мeдвeжий лук	bear onion	Russian	Ural	Latin calque	[181]
*Allium ursinum* L.	*Allium ursinum* L.	мeдвeжий чecнoк	bear garlic	Russian	Ural	Latin calque	[181]
*Allium ursinum* L.	*Allium ursinum* L.	мeдвeдзoв чecнoк	bear garlic	Ruthenian	Vojvodina	Latin calque?	[125]
*Allium ursinum* L.	*Allium ursinum* L.	мeдвeђи лук	bear onion	Serbo-Croat		Latin calque?	[84]
*Allium ursinum* L.	*Allium ursinum* L.	мeдвjeђи лук	bear onion	Serbo-Croat		Latin calque?	[84]
*Allium ursinum* L.	*Allium ursinum*	medvedji zheſen	bear garlic	Slovenian		Latin calque?	[209]
*Allium ursinum* L.	*Allium ursinum*	ayısarımsağı	bear garlic	Turkish	Anatolia	wild	[235]
*Allium ursinum* L.	*Allium ursinum*	ayi sarüsagï	bear garlic	Turkish		Latin calque	[171]
*Allium ursinum* L.	*Allium ursinum* L.	cybula medveža	bear onion	Ukrainian		Latin calque	[130]
*Allium ursinum* L.	*Allium ursinum* L.	łuk medvežyj	bear onion	Ukrainian		Latin calque	[130]
*Allium victorialis* L.	*Allium victorialis* L.=*Allium ursinum* L.	мeдвeжий лук	bear onion	Russian	Middle Ob region	Latin calque	[101]
*Allium xiphopetalum* Aitch. & Baker	*Allium sylvestre*	medvjeđi luk	bear onion	Croatian		Latin calque	[228]
*Aloe* spp.	*Aloe* spp.	karusapp	bear bile	Estonian		status-	[58, 64]
*Aloe* spp.	*Aloe* spp.	karusaba	bear tail	Estonian	Rap	folk etymology	[58]
*Amelanchier ovalis* Medik.	*Amelanchier oval.*	Bärenbirne	bear pear	German, non specified	not specified	bear food	[65]
*Anchusa arvensis* (L.) M. Bieb.	*Anchusa arvensis* (L.) M.Bieb.	kahro ohtja	bear thistle, bear thorn	Estonian		surface	[173]
*Anchusa arvensis* (L.) M. Bieb.	*Anchusa arvensis* (L.) M.Bieb.	karukeel	bear tongue	Estonian		surface	[182]
*Anchusa arvensis* (L.) M. Bieb.	*Lycopsis arvensis* L.	karu osjad	bear horsetails	Estonian		folk etymology	[183]
*Anchusa officinalis* L.	*Anchusa officinalis* L.	karukeel	bear tongue	Estonian	Kuu	shape and surface	[214]
*Anchusa officinalis* L.	*Anchusa officinalis* L.	karuohakas, karuohakad, karuohtjad, kahru-ohtjad	bear thistle, bear thorn	Estonian	Rid, Võn	surface	[58, 64, 73]
*Anchusa officinalis* L.	*Anchusa officinalis* L.	kahru-ohtja, kahro ohtja, karu-ohtja (ohakad)	bear thistle, bear thorn	Estonian		surface	[73, 236]
*Anchusa officinalis* L.	*Anchusa officinalis*	björntass	bear paw	Swedish	Skåne	shape	[155]
*Anemone alpina* L.	*Anemone alpina*	Bärenplumpe	bear paw	German, non specified	not specified	shape?	[65]
*Anemone alpina* L.	*Anemone alpina*	Bärentatze	bear paw	German, non specified	not specified	shape?	[65]
*Anemone alpina* L.	*Pulsatilla alpina*	medveje	bear plant	Slovenian		?	[209]
*Anemone alpina* L.	*Anemone alpina*	medveje	bear plant	Slovenian		?	[84]
*Anemone patens* L.	*Anemone patens* L.	karumunnid	bear balls	Estonian	Tallinn	surface	[58]
*Anemone patens* L.	*Anemone patens* L.	karukell, lilla karukell	bear bell, lillack bear bell	Estonian	North Estonia	surface	[58]
*Anemone patens* L.	*Anemone patens* L.	karusäärepael	bear shank's ribbon	Estonian	Rap	surface	[58]
*Anemone pratensis* L.	*Anemone pratensis* L., *Pulsatilla* Mill.	karukell, karukelluke, karu kellad, karukellad	bear bell(s)	Estonian	North Estonia	surface	[58, 64, 73, 182, 183]
*Anemone pratensis* L.	*Anemone pratensis* L.	karu-kübar	bear hat	Estonian		surface	[236]
*Anemone pratensis* L.	*Anemone pratensis* L.	karumunnid	bear balls	Estonian	Noa	surface	[58]
*Anemone pratensis* L.	*Anemone pratensis* L.	karukollad	bear club moss	Estonian	Kad	surface	[58]
*Anemone pratensis* L.	*Anemone pratensis* L.	karulilled, sinine karulill, kahrulill	bear flower(s), blue bear flower	Estonian	Pal, Äks, Har	surface	[58]
*Anemone pratensis* L.	*Anemone pratensis* L.	karusäärepaelad	bear shank's ribbons	Estonian	Nis	surface	[58]
*Anemone pratensis* L.	*Anemone pratensis* L., *Pulsatilla* Mill.	karukäpad, karu kepad, karu-käpad	bear paws	Estonian	North Estonia, Ha	surface	[58, 73, 183]
*Anemone pratensis* L.	*Anemone pratensis*	bjønneblomme	bear flower	Norwegian	Sigdal	?	[80]
*Anemone pulsatilla* L.	*Anemone puls*.	Bärenblume	bear flower	German, non specified	not specified	shape?	[65]
*Anemone sylvestris* L.	*Anemone sylvestris* L.	karukellad	bear bells	Estonian	JJn, Juu	surface	[58]
*Anemone sylvestris* L.	*Anemone sylvestris* L.	karusilm	bear eye	Estonian	Amb, JMd	surface	[58]
*Angelica archangelica* L.	*Archangelica Hoffm.*	мeдвeжьи дудки	bear pipes	Central Russian	Novgorod	size?	[57]
*Angelica archangelica* L.	*Angelica archangelica* L.	karhunputk, karhunputka, karhunputke	bear thistle, bear thorn	Finnish		folk etymology, size	[55]
*Angelica archangelica* L.	*Angelica archangelica* L.	karhun-yrtti	bear herb	Finnish		folk etymology, size	[82]
*Angelica archangelica* L.	*Angelica archangelica* L.	karunputet	bear pipe	Ingrian		folk etymology, size	[56]
*Angelica archangelica* L.	*Angelica archangelica* L.	karhuntruba	bear pipe	Izhorian		folk etymology, size	[56]
*Angelica archangelica* L.	*Angelica archangelica* L.	karupuded	bear pipe	Izhorian		folk etymology, size	[56]
*Angelica archangelica* L.	*Angelica archangelica* L.	karuputki	bear pipe	Izhorian		folk etymology, size	[56]
*Angelica archangelica* L.	*Angelica archangelica*	guovžžarássi	bear plant	North Saami		?	[212]
*Angelica archangelica* L.	*Archangelica* Hoffm.	мeдвeжьи дудки	bear pipes	Northern Russian	Vologda	size?	[57]
*Angelica archangelica* L.	*Archangelica* Hoffm.	мeдвeдoк	little bear	Russian	Middle Ural	?	[57]
*Angelica archangelica* L.	*Archangelica* Hoffm.	мeдвeжья дудкa	bear pipe	Russian	Ekaterinburg region	?	[57]
*Angelica archangelica* L.	*Archangelica* Hoffm.	мeдвeжкa	little bear	Russian	Novosibirsk region	?	[57]
*Angelica archangelica* L.	*Archangelica* Hoffm.	мeдвeжкa	little bear	Russian	Ekaterinburg region	?	[57]
*Angelica archangelica* L.	*Angelica* ?	björnkål	bear cabbage	Swedish	Dalarna	size?	[155]
*Angelica archangelica* L.	*Angelica archangelica*	björnstut	bear trumpet	Swedish	Lappland	size?	[155]
*Angelica archangelica* L.	*Angelica silvestris*	björnflo	bear plant	Swedish	Lappland	size?	[155]
*Angelica archangelica* L.	*Angelica silvestris*	björnstut	bear trumpet	Swedish	Lappland, Ångermanland, Västerbotten, Lappland	size?	[155]
*Angelica archangelica* subsp. litoralis (Wahlenb.) Thell.	*Angelica archangelica* ssp. litoralis	björnfloka	bear plant	Swedish	Österbotten	size?	[155]
*Angelica* spp.	*Angelica* spp.	karhun yrti	bear herb	Finnish		folk etymology, size	[82]
*Angelica* spp.	*Angelica* spp.	karhun putki, carhun putki	bear pipe	Finnish		folk etymology, size	[82]
*Angelica sylvestris* L.	*Angelica sylvestris* L.	мeдвeжьи дудки	bear pipes	Central Russian	Novgorod	size?	[57]
*Angelica sylvestris* L.	*Angelica sylvestris* L.	karhun-yrtti	bear herb	Finnish		folk etymology, size	[82]
*Angelica sylvestris* L.	*Angelica sylvestris* L.	karhunputki, carhunputki, Karhunputk	bear pipe	Finnish		folk etymology, size	[55, 82]
*Angelica sylvestris* L.	*Angelica sylvestris* L.	karhuntruba	bear pipe	Ingrian		folk etymology, size	[56]
*Angelica sylvestris* L.	*Angelica sylvestris* L.	karunputet	bear pipe	Ingrian		folk etymology, size	[56]
*Angelica sylvestris* L.	*Angelica sylvestris* L.	karuputki	bear pipe	Izhorian		folk etymology, size	[56]
*Angelica sylvestris* L.	*Angelica sylvestris* L.	мeдвeжья пучкa	bear cow-parsnip	Russian	Middle Ob region	bear food?	[101]
*Angelica sylvestris* L.	*Angelica sylvestris* L.	мeдвeжьи пучки	bear cow-parsnip	Russian	Ural	size?	[57]
*Angelica sylvestris* L.	*Angelica sylvestris*	björnkumminstrånge	rough bear cummin	Swedish	Dalarna	bear food	[155]
*Angelica sylvestris* L.	*Angelica syilvestris*	björnloka	bear plant	Swedish	Gästrikland, Dalarna, Västmanland	size?	[155]
*Angelica sylvestris* L.	*Angelica sylvestris*	björnlop	bear plant	Swedish	Dalarna	size?	[155]
*Angelica sylvestris* L.	*Angelica sylvestris*	björnpipa	bear pipe	Swedish	Västmanland, Dalarna, Västergötland	size?	[155]
*Angelica sylvestris* L.	*Angelica sylvestris*	björngräs	bear grass	Swedish	Dalarna	size?	[155]
*Angelica sylvestris* L.	*Angelica sylvestris*	björnslök	bear plant	Swedish	Värmland	size?	[155]
*Angelica sylvestris* L.	*Angelica sylvestris*	björnflor, björnflo	bear plant	Swedish	Lappland	size?	[154, 155]
*Angelica sylvestris* L.	*Angelica sylvestris*	björnstut	bear trumpet	Swedish	Västerbotten	shape?	[154]
*Angelica sylvestris* L.	*Angelica sylvestris* L.	byönngras	bear grass	Swedish	Älvdalen	?	[223]
*Angelica sylvestris* L.	*Angelica sylvestris* L.	byönnkumåstraingg	bear caraway plant	Swedish	Älvdalen	?	[223]
*Angelica sylvestris* L.	*Angelica sylvestris* L.	byönnkumåstraungg	bear caraway plant	Swedish	Älvdalen	?	[223]
*Angelica ursina* (Rupr.) Maxim.	*Angelophyllum ursinum*	мeдвeжий кopeнь	bear root	Russian		Latin calque	[116]
*Angelica ursina* (Rupr.) Maxim.	*a Kamchatka plant*	мeдвeжий кopeнь	bear root	Russian	Kamchatka	Latin calque?	[57]
*Antennaria dioica* (L.) Gaertn.	*Antennaria dioica*	medvědí tlapičky	bear paws	Czech	Chodov	surface	[83]
*Antennaria dioica* (L.) Gaertn.	*Antennaria dioica* (L.) Gaertn.	karukäpp	bear paw	Estonian	Amb, JMd	shape	[58]
*Antennaria dioica* (L.) Gaertn.	*Antennaria dioica*	Bärentatze	bear paw	German, non specified	not specified	shape	[65]
*Antennaria dioica* (L.) Gaertn.	*Antennaria dioica*	Bärenpratze	bear paw	German, non specified	not specified	shape	[65]
*Antennaria dioica* (L.) Gaertn.	*Antennaria dioica*	medvedove tačice	bear little paws	Slovenian		shape?	[84]
*Antennaria dioica* (L.) Gaertn.	*Antennaria dioica* (L.) Gaertn.	byönntasser	bear’s paws	Swedish	Älvdalen	shape	[223]
*Anthriscus sylvestris* (L.) Hoffm.	*Anthriscus sylvestris* (L.) Hoffm.	karhunputki	bear pipe	Izhorian		folk etymology	[172]
*Anthriscus sylvestris* (L.) Hoffm.	*Anthriscus sylvestris* (L.) Hoffm.	karuputki	bear pipe	Votic		folk etymology	[56]
*Anthyllis vulneraria* L.	*Anthyllis vulneraria* L.	Bärenklee	bear clover	German, non specified	not specified	surface?, shape?	[65]
*Anthyllis vulneraria* L.	*Anthyllis vulneraria* L.	Bärenpratze	bear paw	German, non specified	not specified	surface?, shape?	[65]
*Anthyllis vulneraria* L.	*Anthyllis vulneraria* L.	Bärentappe	bear paw	German, non specified	not specified	surface?, shape?	[65]
*Anthyllis vulneraria* L.	*Anthyllis vulneraria* L.	Bärentatze	bear paw	German, non specified	not specified	surface?, shape?	[65]
*Anthyllis vulneraria* L.	*Anthyllis vulneraria*	medvetalpa	bear’s paw	Hungarian	Gyimes (Transylvania)	surface	[161]
*Anthyllis vulneraria* L.	*Anthyllis vulneraria* L.	medvjeka	bear plant	Slovenian		?	[89, 144]
*Anthyllis vulneraria* L.	*Anthyllis vulneraria* L.	medvejčka	bear plant	Slovenian		?	[89, 144]
*Antirrhinum majus* L.	*Antirrhinum majus*	medveszája	bear’s mouth	Hungarian	no data (Hungary or Transylvania)	shape	[176]
Apiaceae	*Apiaceae*, unidentified taxon	ursurèdda, sursurèdda	little female bear	Sardinian	Bari Sardo area, Ogliastra, eastern Sardinia	?	[142]
*Arbutus unedo* L.	*Arbutus unedo* L.	mbriachelle dë l'ursë	intoxicating little bear fruit	Italian, central	Lower Valle Peligna, upper Val Pescara, Abruzzo, central Italy	bear food	Cianfaglione unpublished
*Arbutus unedo* L.	*Arbutus unedo*	мeчje гpoжђe	bear grapes	Serbo-Croat		size?	[84]
*Arbutus unedo* L.	*Arbutus unedo*	ayı çileği	bear strawberry	Turkish	Anatolia	bear food	[169]
*Arbutus unedo* L.	*Arbutus unedo*	ayı çileği	bear strawberry	Turkish	Muğla (SW Anatolia)	shape, bear food	[169]
*Arbutus unedo* L.	*Arbutus unedo*	ayıyemişi	bear fruit	Turkish	İzmit (NW Anatolia)-Marmara Region	bear food	[178]
*Arbutus unedo* L.	*Arbutus unedo*	ayı yemişi	bear fruit	Turkish	Anatolia	bear food	[227]
*Arbutus unedo* L.	*Arbutus unedo*	ayı yemişi	bear fruit	Turkish	Anatolia	bear food	[227]
*Arctium lappa* L.	*Arctium lappa* L.	medvědí	bears	Czech		size?	[61]
*Arctium minus* (Hill) Bernh.	*Arctium minus* (Hill) Bernh.	ayı kabağı	bear zucchini	Turkish	Ankara (Inner Anatolia)	status -	[165]
*Arctium minus* (Hill) Bernh.	*Arctium minus* (Hill) Bernh.	ayı pıtırağı	bear cocklebur	Turkish	Yalova (NW Anatolia)	size	[227]
*Arctium tomentosum* Mill.	*Arctium tomentosum Mill.*	kahrutakjas	bear burr	Estonian	Se	shape and surface	[58]
*Arctopus* spp.	*Arctopus* L.	medvjeđa šapa	bear paw	Croatian	no location	shape?, surface?, Latin calque?	[126, 138, 244]
*Arctostaphylos* spp.	*Arctostaphylos* L.	мeдвeдильник	bear plant	Russian	Ural	Latin calque?	[57]
*Arctostaphylos uva-ursi* (L.) Spreng.	*Arctostaphylos* uva-ursi	Rrush arushe; Rrush harushe	bear grape	Albanian	North Albania, Kosovo	bear food	[136]
*Arctostaphylos uva-ursi* (L.) Spreng.	*Arctostaphylos* uva-ursi	ayïġulaγï	bear’s ear	Azeri		shape	[6]
*Arctostaphylos uva-ursi* (L.) Spreng.	*Arctostaphylos * *uva-ursi*	ayïgäghï	bear ear	Azeri		shape	[171]
*Arctostaphylos uva-ursi* (L.) Spreng.	*Arctostaphylos*	мядзвeджыя вушкi	bear little ears	Belarussian	Grodn.	bear food? status-?	[79]
*Arctostaphylos uva-ursi* (L.) Spreng.	*Arctostaphylos*	мядзвeджыя вушкi	bear little ears	Belarussian	Minsk.	bear food? status-?	[79]
*Arctostaphylos uva-ursi* (L.) Spreng.	*Arctostaphylos* *uva-ursi* Spreng.	мeчe гpoздe	bear grapes	Bulgarian		Latin calque	[86]
*Arctostaphylos uva-ursi* (L.) Spreng.	*Arctostaphylos* *uva ursi* (L.) Spreng.	medveje grozje	bear grapes	Croatian	Podvežica	Latin calque	[126, 162]
*Arctostaphylos uva-ursi* (L.) Spreng.	*Arctostaphylos* *uva ursi* (L.) Spreng.	medvjedika	bear plant	Croatian	no location	Latin calque	[126, 217]
*Arctostaphylos uva-ursi* (L.) Spreng.	*Arctostaphylos* *uva ursi* (L.) Spreng. (Arctostaphylos officinalis Wimm. et Grab.)	medvjedica	she-bear	Croatian	no location	Latin calque	[126, 138, 244]
*Arctostaphylos uva-ursi* (L.) Spreng.	Arctostaphylos	medvjedica	she-bear	Croatian	no location	Latin calque	[126, 138, 245]
*Arctostaphylos uva-ursi* (L.) Spreng.	*Arctostaphylos* *uva ursi* (L.) Spreng.	medvjetka	bear plant	Croatian	no location	Latin calque?	[126, 228, 229]
*Arctostaphylos uva-ursi* (L.) Spreng.	*Arctostaphylos* *uva ursi* Spr.	nedvědice	bear plant	Czech		Latin calque?	[60, 72]
*Arctostaphylos uva-ursi* (L.) Spreng.	*Arctostaphylos* *uva-ursi* (L.) Spreng.	medvědík	little bear	Czech		bear food? status-?	[61]
*Arctostaphylos uva-ursi* (L.) Spreng.	*Arctostaphylos* *uva-ursi* (L.) Spreng.	medvědí hrozen	bear grapes	Czech		Latin calque	[61]
*Arctostaphylos uva-ursi* (L.) Spreng.	*Arctostaphylos * *uva-ursi* (L.) Spreng.	medvědí hrozny	bear grapes	Czech		Latin calque	[61]
*Arctostaphylos uva-ursi* (L.) Spreng.	*Arctostaphylos* *uva-ursi*	bjørnebaer	bear berry	Danish	Denmark	Latin calque	[187]
*Arctostaphylos uva-ursi* (L.) Spreng.	*Arctostaphylos* *uva-ursi* (L.) Spreng.	karukobar, karukobarad	bear cluster(s)	Estonian		Latin calque	[64]
*Arctostaphylos uva-ursi* (L.) Spreng.	*Arctostaphylos* *uva-ursi* (L.) Spreng.	karupohlad	bear lingonberry	Estonian	Vig	inedibility	[64]
*Arctostaphylos uva-ursi* (L.) Spreng.	*Arctostaphylos* *uva-ursi* (L.) Spreng.	karuviinapuu-marjalehed	bear grapetree berries leaves	Estonian		Latin calque	[64]
*Arctostaphylos uva-ursi* (L.) Spreng.	*Arctostaphylos * *uva-ursi* (L.) Spreng.	karulehed, kahruliht	bear leaves	Estonian	Vig, Har	Latin calque	[64]
*Arctostaphylos uva-ursi* (L.) Spreng.	*Arctostaphylos* *uva-ursi* (L.) Spreng.	karupuhulgalehed	bear lingonberry leaves	Estonian		Latin calque	[64]
*Arctostaphylos uva-ursi* (L.) Spreng.	*Arctostaphylos* *uva-ursi* (L.) Spreng.	karutee	bear tea	Estonian		Latin calque	[64]
*Arctostaphylos uva-ursi* (L.) Spreng.	*Arctostaphylos* *uva-ursi* (L.) Spreng.	karukapsas	bear cabbage	Estonian	Rap	Latin calque	[58]
*Arctostaphylos uva-ursi* (L.) Spreng.	*Arctostaphylos* *uva-ursi* (L.) Spreng.	karukõrvad	bear ears	Estonian	Nrv	Latin calque	[58]
*Arctostaphylos uva-ursi* (L.) Spreng.	*Arctostaphylos * *uva-ursi* (L.) Spreng.	uë ursine	bear bilberry	Friulian	Friuli, north-eatern Italy	bear food?	[153]
*Arctostaphylos uva-ursi* (L.) Spreng.	*Arctostaphylos * *uva-ursi* (L.) Spreng.	pirusiel da l'ors	bear bilberry	Friulian	Carnia, Friuli, north-eastern Italy	status-	[74, 153]
*Arctostaphylos uva-ursi* (L.) Spreng.	*Arctostaphylos* *uva-ursi*	Bären-Chris	?	German, non specified	not specified	Latin calque	[65]
*Arctostaphylos uva-ursi* (L.) Spreng.	*Arctostaphylus* *uva-ursi*	Bärenkraut	bear herb	German, non specified	not specified	Latin calque	[65]
*Arctostaphylos uva-ursi* (L.) Spreng.	*Artostaphylus* *uva-ursi*	Barentraube	bear grape	German, non specified	not specified	Latin calque	[65]
*Arctostaphylos uva-ursi* (L.) Spreng.	*Arctostaphylos* *uva-ursi*	Bärenbeere	bear berry	German, non specified	not specified	Latin calque	[65]
*Arctostaphylos uva-ursi* (L.) Spreng.	*Arctostaphylos* *uva-ursi*	medvegerezd	bear’s slice	Hungarian	Transylvania	wild	[150]
*Arctostaphylos uva-ursi* (L.) Spreng.	*Arctostaphylos * *uva-ursi* (L.) Spreng.	karhumarja	bear berry	Izhorian		Latin calque	[56]
*Arctostaphylos uva-ursi* (L.) Spreng.	*Arctostaphylos* *uva-ursi* (L.) Spreng.	uva d'urso; uva ursina; uva urzina; evæ orsina; uva mursine; uvæ orsina; uva urzinæ; uvettæ dë l'ursë	bear grape	Italian, central and southern	Abruzzo, central Italy	wild, status-	([90], Cianfaglione unpublished)
*Arctostaphylos uva-ursi* (L.) Spreng.	*Arctostaphylos* *uva-ursi* (L.) Spreng.	ua orsina	bear bilberry	Italian, northern	Istria, Friuli, northern Italy	wild, status-	[153]
*Arctostaphylos uva-ursi* (L.) Spreng.	*Arctostaphylos * *uva-ursi* (L.) Spreng.	ciate d'ors	bear feet	Italian, northern	Arco area, Trentino, north-eatern Italy	shape	[115]
*Arctostaphylos uva-ursi* (L.) Spreng.	*Arctostaphylos * *uva-ursi* (L.) Spreng.	brussei de l'orso	bear bilberries	Italian, northern	Primiero area, Trentino, north-eastern Italy	status-	[115]
*Arctostaphylos uva-ursi* (L.) Spreng.	*Arctostaphylos * *uva-ursi* (L.) Spreng.	uva dl'ours	bear grape	Italian, northern	Cuneo area, Piedmont, north-western Italy	wild, status-	[115]
*Arctostaphylos uva-ursi* (L.) Spreng.	*Arctostaphylos * *uva-ursi* (L.) Spreng.	granteni d'ors; broc da l'ors	bear lingonberries	Italian, northern	Pinè and Val Rendena, Trentino, north-eastern Italy	status-	[115, 206]
*Arctostaphylos uva-ursi* (L.) Spreng.	*Arctostaphylos * *uva-ursi* (L.) Spreng.	pan d'ors	bear bread	Italian, northern	Bergamo area, Lombardy, northern Italy	bear food	[74]
*Arctostaphylos uva-ursi* (L.) Spreng.	*Arctostaphylos* *uva-ursi* (L.) Spreng.	uga d'ursu	bear grape	Italian, northern	Chiavari area, Liguria, north-western Italy	wild, status-	[74]
*Arctostaphylos uva-ursi* (L.) Spreng.	*Arctostaphylos * *uva-ursi* (L.) Spreng.	uga dell'orso	bear grape	Italian, northern	Sondrio area, Lombardy, northern Italy	wild, status-	[74]
*Arctostaphylos uva-ursi* (L.) Spreng.	*Arctostaphylos * *uva-ursi* (L.) Spreng.	ova orsèina	bear grape	Italian, northern	Reggio area, Emilia, northern Italy	wild, status-	[74]
*Arctostaphylos uva-ursi* (L.) Spreng.	*Arctostaphylos* *uva-ursi* (L.) Spreng.	cerasella d'orso	bear hawthorn berry	Italian, southern	Terra di Lavoro area, southern Italy	status-, bear food?	[74]
*Arctostaphylos uva-ursi* (L.) Spreng.	*Arctostaphylos* *uva-ursi* (L.) Spreng.	garnate del óurs; garnéte de órs; granats dl'aurs; granate de l'ours	bear bilberries	Ladin	Val Cordevole, Val di Fassa, Val Badia and Val Gardena, Veneto and Trentino, north-eastern Italy	Latin calque?, status-?	[207]
*Arctostaphylos uva-ursi* (L.) Spreng.	*Arctostaphylos * *uva-ursi* L.	meškos ausys	bear ears	Lithuanian	Lithuania	shape, bear food	[87]
*Arctostaphylos uva-ursi* (L.) Spreng.	*Arctostaphylos * *uva ursi* Spr.	мeдвeжий винoгpaд	bear grapes	Northern Russian	Vologda	Latin calque	[57]
*Arctostaphylos uva-ursi* (L.) Spreng.	*Arctostaphylos* *uva-ursi* (L.) Spreng.	bjørnebær	bear berry	Norwegian	Eidskog, Budal	Latin calque	[80]
*Arctostaphylos uva-ursi* (L.) Spreng.	*Arctostaphylos * *uva ursi* Spr.	mącznica niedzwiedziny	bear mealberry	Polish		bear food? status-?	[116]
*Arctostaphylos uva-ursi* (L.) Spreng.	*Arctostaphylos* *uva ursi* Spr.	niedźwiedzie grono	bear grapes	Polish		shape	[72]
*Arctostaphylos uva-ursi* (L.) Spreng.	*Arctostaphylos* *uva ursi* (L.) Spreng.	вeдмeжьи ушки	bear little ears	Russian	Middle Ob region	bear food? status-?	[101]
*Arctostaphylos uva-ursi* (L.) Spreng.	*Arctostaphylos* *uva ursi* (L.) Spreng.	мeдвeжьe уxo	bear ear	Russian	Middle Ob region	bear food? status-?	[101]
*Arctostaphylos uva-ursi* (L.) Spreng.	*Arctostaphylos* *uva ursi* (L.) Spreng.	мeдвeжьe ушкo	bear little ear	Russian	Middle Ob region	bear food? status-?	[101]
*Arctostaphylos uva-ursi* (L.) Spreng.	*Arctostaphylos* *uva ursi* (L.) Spreng.	мeдвeжья ягoдa	bear berry	Russian	Middle Ob region	Latin calque?	[101]
*Arctostaphylos uva-ursi* (L.) Spreng.	*Arctostaphylos* *uva ursi* (?)	мeдвeдильник	bear plant	Russian	Ural	Latin calque?	[181]
*Arctostaphylos uva-ursi* (L.) Spreng.	*Arctostaphylos* *uva-ursi* L.	мeдвeжьи ушки	bear little ears	Russian	Ural	Latin calque?	[181]
*Arctostaphylos uva-ursi* (L.) Spreng.	*Arctostaphylos* *uva ursi* L.	мeдвeдзoвo уxo	bear ear	Ruthenian	Vojvodina	bear food? status-?	[125]
*Arctostaphylos uva-ursi* (L.) Spreng.	*Arctostaphylos * *uva ursi* (L.) Spreng.	мeдвeђe гpoжђe	bear grapes	Serbo-Croat		Latin calque?	[84]
*Arctostaphylos uva-ursi* (L.) Spreng.	*Arctostaphylos* *uva ursi* (L.) Spreng.	мeдвeткa	bear plant	Serbo-Croat		Latin calque?	[84]
*Arctostaphylos uva-ursi* (L.) Spreng.	*Arctostaphylos* *uva ursi* (L.) Spreng.	мeдвиjeдицa	she-bear	Serbo-Croat		Latin calque?	[84]
*Arctostaphylos uva-ursi* (L.) Spreng.	*Arctostaphylos* *uva ursi* Spr.	мeдвeдoвo уxo	bear ear	Serbo-Croat		Latin calque?	[84]
*Arctostaphylos uva-ursi* (L.) Spreng.	*Arctostaphylos* uva ursi (L.) Spreng.	мeчиje гpoжђe	bear grapes	Serbo-Croat		Latin calque?	[84]
*Arctostaphylos uva-ursi* (L.) Spreng.	*Arctostaphylos*	medvedica	she-bear	Slovak	Slovakia	Latin calque?	[62]
*Arctostaphylos uva-ursi* (L.) Spreng.	*Arctostaphylos*	nedvedica	she-bear	Slovak	Slovakia	Latin calque?	[62]
*Arctostaphylos uva-ursi* (L.) Spreng.	*Arctostaphylos* *uva ursi* (L.) Spreng.	medvedovo uho	bear ear	Slovenian		bear food? status-?	[89]
*Arctostaphylos uva-ursi* (L.) Spreng.	*Arctostaphylos* *uva ursi* (L.) Spreng.	medvedove črešnje	bear sweet cherries	Slovenian		Latin calque?	[89]
*Arctostaphylos uva-ursi* (L.) Spreng.	*Arctostaphylos* *uva ursi* (L.) Spreng.	medvedji grozdič	bear grapes	Slovenian		Latin calque	[84, 89]
*Arctostaphylos uva-ursi* (L.) Spreng.	*Arctostaphylos* *uva ursi* Spr.	medvedje uho	bear ear	Slovenian		Latin calque?	[84, 89]
*Arctostaphylos uva-ursi* (L.) Spreng.	*Arctostaphylos* *uva ursi* (L.) Spreng.	medvedovo grozdjiče	bear grapes	Slovenian		Latin calque	[84, 89]
*Arctostaphylos uva-ursi* (L.) Spreng.	*Arctostaphylos*	mjedwjedźica	she-bear	Sorbian		Latin calque?	[186]
*Arctostaphylos uva-ursi* (L.) Spreng.	*Arctostaphylos* *uva ursi* (L.) Spreng.	holanska mjedwjedźica	forest she-bear	Sorbian		Latin calque?	[186]
*Arctostaphylos uva-ursi* (L.) Spreng.	*Arctostaphylos* *uva-ursi*	björnbär	bear berry	Swedish	Norrbotten	Latin calque	[155]
*Arctostaphylos uva-ursi* (L.) Spreng.	*Arctostaphylos* *uva-ursi*	björnebär	bear berry	Swedish	Sör-Tröndelag	Latin calque	[155]
*Arctostaphylos uva-ursi* (L.) Spreng.	*Arctostaphylos* *uva-ursi*	ayı üzümü	bear grape	Turkish	Anatolia	bear food	[160]
*Arctostaphylos uva-ursi* (L.) Spreng.	*Arbutus* *uva ursi*	ayı üzümü	bear grape	Turkish	Anatolia	shape, bear food	[59]
*Arctostaphylos uva-ursi* (L.) Spreng.	*Arctostaphylos* *uva ursi* Spr.	вушкo вeдмeжe	bear ear	Ukrainian		bear food? status-?	[180]
*Arctostaphylos uva-ursi* (L.) Spreng.	*Arctostaphylos* *uva ursi* Spr.	мeдвeжий винoгpaд	bear grapes	Ukrainian		Latin calque	[130, 219]
*Arctostaphylos uva-ursi* (L.) Spreng.	*Arctostaphylos* *uva ursi* Spr.	мeдвeжi ягoди	bear berries	Ukrainian		Latin calque	[130, 219]
*Arctostaphylos uva-ursi* (L.) Spreng.	*Arctostaphylos* *uva ursi* Spr.	ушкo мeдвeжe	bear ear	Ukrainian, Central-Polessian		bear food? status-?	[180]
*Arctostaphylos uva-ursi* (L.) Spreng.	*Arctostaphylos * *uva ursi* Spr.	мeдвeжинa	bear plant	Ukrainian, East-Polessian		bear food? status-?	[180]
*Arctostaphylos uva-ursi* (L.) Spreng.	*Arctostaphylos* *uva ursi* Spr.	мeдвeдячi ягiдки	bear berries	Ukrainian, Pokutje-Bukovina	Bukovina	Latin calque?	[130, 219]
*Arctostaphylos uva-ursi* (L.) Spreng.	*Arctostaphylos* *uva ursi* Spr.	уxo мeдвeжe	bear ear	Ukrainian, Volhynian		bear food? status-?	[180]
*Arctostaphylos uva-ursi* (L.) Spreng.	*Arctostaphylos* *uva ursi* (L.) Spreng.	mjedwjedźica	she-bear	Upper-Sorbian		Latin calque?	[121]
*Arctotis* spp.	*Arctotis*	Bärenohr	bear ear	German, non specified	not specified	Latin calque	[65]
*Arctotis* spp.	*Arctotis sp.*	ayıkulağı	bear ear	Turkish		shape	[171]
*Arctotis* spp.	*Arctotis sp.*	ayï otu	bear’s grass	Turkish		?	[171]
*Arctotis* spp.	*Arctotis sp*.	ayıkulaği	bear’s ear	Turkish		shape	[6]
*Arctotis* spp.	*Arctotis sp.*	ayï pencesi	bear’s foot	Turkmen		shape	[171]
*Arctotis stoechadifolia* P.J. Bergius	*Arctotis stoechadifolia* P.J. Bergius	ayı kulağı	bear ear	Turkish	Anatolia	shape	[226]
*Arctotis stoechadifolia* P.J. Bergius	*Arctotis stoechadifolia* P.J. Bergius	ayı kulağı	bear ear	Turkish	Anatolia	shape	[226]
*Arctous alpina* (L.) Nied.	Arctostaphylos alpina	Bärenbeere	bear berry	German, non specified	not specified	Latin calque	[65]
*Arctous alpina* (L.) Nied.	*Arctostaphylos alpinus* (L.) Spreng.	uga dell'orso salvatico	wild bear grape	Italian, northern	Sondrio area, Lombardy, northern Italy	status-	[74]
*Arctous alpina* (L.) Nied.	*Arctostaphylos* alpinus	guovžžalasta	bear leaf	North Saami		?	[129]
*Arctous alpina* (L.) Nied.	*Arctostaphylos* alpine	guovžžamuorji	bear berry	North Saami		?	[212]
*Arctous alpina* (L.) Nied.	*Arctostaphylos* alpina	björnebär	bear berry	Norwegian	Vest-Agder	Latin calque	[155]
*Arctous alpina* (L.) Nied.	*Arctostaphylos* alpine	bjønnbær	bear berry	Norwegian	Ranen, Northern Norway	Latin calque	[15, 112]
*Arctous alpina* (L.) Nied.	*Arctostaphylos* alpina	hórska mjedwjedźica	mountain she-bear	Sorbian		Latin calque?	[186]
*Arctous alpina* (L.) Nied.	*Arctostaphylos* alpinus	bïernenmuerjie	bear berry	South Saami		Latin calque	[170]
*Arctous alpina* (L.) Nied.	*Arctostaphylos* alpine	björnbär	bear berry	Swedish	North Lapland	status-	[15]
*Arctous alpina* (L.) Nied.	*Arctostaphylos* alpina	björnbär	bear berry	Swedish	Lappland	status-	[155]
*Arctous* spp.	*Arctous*	medvedík	little bear	Slovak	Slovakia	Latin calque?	[62]
*Arisaema triphyllum* (L.) Schott	*Arum triphyllum*	medvědí kořen	bear root	Czech		?	[61]
*Aristolochia clematitis* L.	*Aristolochia clematitis*	medvedova grašca	bear vetch	Slovenian		bear food?	[84, 89]
*Aristolochia clematitis* L.	*Aristolochia clematitis*	medvedova grašica	bear vetch	Slovenian		bear food?	[84, 89]
*Arum italicum* Mill.	*Arum italicum* Mill.	мeчe вpeтeнo	bear spindle	Bulgarian	Berkovica area	shape, size	[86]
*Arum maculatum* L.	*Arum maculatum* All.	мeчкинa пчeнкa	bear maize	Bulgarian	Kozica village, Kichevo area	shape	[86]
*Aruncus dioicus* (Walter) Fernald	*Aruncus dioicus* (Walter) Fernald (*Spiraea aruncus* L.)	medvedovina	bear plant	Croatian	Hrvatsko zagorje	?	[126, 138]
*Aruncus dioicus* (Walter) Fernald	*Aruncus silvester*	мeдвeдoвинa	bear plant	Serbo-Croat		?	[84]
*Aruncus dioicus* var. vulgaris (Maxim.) H. Hara	*Aruncus sylv*.	Barenmutter-strauss	bear mother bouquet	German, non specified	not specified	folk etymology?	[65]
*Aruncus dioicus* var. vulgaris (Maxim.) H. Hara	*Aruncus silvester* Kostel. = Spiraea aruncus	medvejka	bear plant	Slovenian		?	[89, 144]
*Aruncus dioicus* var. vulgaris (Maxim.) H. Hara	*Aruncus silvester* Kostel. = Spiraea aruncus	medvedovo latje	bear stalks	Slovenian		?	[89]
*Aruncus sylvester* Kostel. ex Maxim.	*Spiraea aruncus*	medvêjka	bear plant	Slovenian		?	[149]
*Aruncus sylvester* Kostel. ex Maxim.	*Spiraea aruncus*	medvêjak	bear plant	Slovenian		?	[149]
*Asarum europaeum* L.	*Asarum* *europaeum* L.	вeдмeжe вуxo	bear ear	Ukrainian, Podolian	Odessa	shape?	[180, 215]
*Asparagus officinalis* L.	*Asparagus* *officinalis* L.	мeдвeдник	bear plant	Russian	Tobolsk area	size?	[218]
*Asparagus officinalis* L.	*Asparagus* *officinalis* L.	мeдвeжник	bear plant	Russian	Tobolsk area	size?	[218]
*Asparagus officinalis* L.	*Asparagus* *officinalis* L.	мeдвeдкa	bear plant	Russian	Bashkortostan	size? wild?	[57]
*Asparagus* spp.	*Asparagus* L.	мeдвeжьe дepeвo	bear tree	Russian	Tobolsk area	size	[57]
*Asplenium scolopendrium* L.	*Scolopendrium vulgare*	мeчjи jeзик	bear tongue	Serbo-Croat		shape?	[84]
*Aster amellus* L.	*Aster amellus*	ayıkulaği	bear’s ear	Turkish		shape	[6]
*Astragalus glycyphyllos* L.	*Astragalus glyc*.	Bärenklee	bear clover	German, non specified	not specified	bear food?	[65]
*Astragalus glycyphyllos* L.	*Astragalus glycyph*.	Bärenschote	bear pod	German, non specified	not specified	bear food?	[65]
*Astragalus glycyphyllos* L.	*Astragalus glycyphyllus* L.	koren medvedov	bear root	Slovenian		German calque	[144]
*Athamanta cretensis* L.	*Athamanta cret*.	Bärenwurz	bear root	German, non specified	not specified	shape?, surface?	[65]
*Atropa belladonna* L.	*Atropa belladona* L.	мeчкини уши	bear ears	Bulgarian		shape, surface	[86]
*Atropa belladonna* L.	*Atropa belladonna* L.	karumustikas	bear blueberry	Estonian	Kan, Kul, Vai	folk etymology	[56, 58, 64, 214]
*Atropa belladonna* L.	*Atropa bell*.	Bärenmutz	?	German, non specified	not specified	?	[65]
*Atropa belladonna* L.	*Atropa bell*.	Bärenwurz	bear root	German, non specified	not specified	?	[65]
*Atropa belladonna* L.	*Atropa belladonna* L.	ayı çileği	bear strawberry	Turkish	Trabzon (NE Anatolia)	dangerousness	[227]
*Atropa belladonna* L.	*Atropa belladonna* L.	ayı libargası	bear blueberry	Turkish/Laz language	Trabzon (NE Anatolia)	dangerousness	[227]
*Atropa belladonna* L.	*Atropa belladonna* L.	ayı liforu	bear blueberry	Turkish/Laz language	Trabzon (NE Anatolia)	dangerousness	[227]
*Avena fatua* L.	*Avena fatua* L.	karu-kaerad	bear oats	Estonian		inedibility	[73]
Avenella flexuosa (L.) Drejer	*Deschampsia flexuosa*	björnjägal	bear’s grass	Swedish	Northern Sweden	?	[224]
*Avenula pubescens* (Huds.) Dumort.	*Helictotrichon pubescens* (Huds.) Schult. & Schult.f.	karukaer, karu-kaer, kahrukaer	bear oat	Estonian		folk etymology	[73]
*Begonia metallica* W.G.Sm.	*Begonia metallica* G. Smith.	мeдвeжьe уxo	bear ear	Russian	Middle Ob region	surface	[101]
*Bidens tripartita* L.	*Bidens tripartita* L.	вeдмeжи ушки	bear little ears	Ukrainian	Zhitomir	shape?	[215]
*Blechnum spicant* (L.) Sm.	*Blechnum spicant* (L.) Sm.	bjørnekam	bear comb	Norwegian		?	[80]
*Boletus edulis* Bull.	*Boletus edulis*	мeдвeдки	bear plant	Central Russian	Novgorod	status +	[57]
*Boletus edulis* Bull.	*Boletus edulis*	мeдвeжaник	bear plant	Central Russian	Tver	status +	[57]
*Boletus edulis* Bull.	old *Boletus ed*.	мeдвeжaник	bear plant	Central Russian	Tver	status +	[57]
*Boletus edulis* Bull.	old *Boletus ed*.	мeдвeжник	bear plant	Central Russian	Novgorod	status +	[57]
*Boletus edulis* Bull.	*Boletus edulis* and other *Boletus* spp.	medvegomba	bear’s mushroom	Hungarian	Tatrang, Csík, Sepsiszentgyörgy, Csinód, Gyimesfelsőlok (Transylvania)	surface	([99, 100, 204], Molnár and Babai unpublished, Frendl, Papp unpublished)
*Boletus edulis* Bull.	old *Boletus ed*.	мeдвeдoвик	bear plant	Northern Russian	Vologda	size? status +?	[57]
*Boletus edulis* Bull.	*Boletus edulis*	мeдвeжины	bear plant	Northern Russian	Vologda	status +?	[57]
*Boletus edulis* Bull.	*Boletus edulis*	мeдвeжник	bear plant	Northern Russian	Olonets, North	status+?	[57]
*Boletus edulis* Bull.	*Boletus edulis* Bull.	мeдвeдapa	bear plant	Serbo-Croat		?	[84]
*Boletus edulis* Bull.	*Boletus edulis* Bull.	ayıköşkü mantarı	bear pavilion mushroom	Turkish	Anatolia	shape, size	[146]
*Boletus satanas* Lenz	*Boletus satanas* Lenz	мeдвeдapa	bear plant	Serbo-Croat		dangerousness (toxic)?	[84]
*Boletus* spp.	*Boletus* Fr.	ayı mantarı	bear mushroom	Turkish	Anatolia	shape, size	[146]
*Borago officinalis* L.	*Borago officinalis* L.	karurohi	bear grass	Estonian	Trm	surface	[58]
*Bovista plumbea* Pers.	*Bovista plumbea* Pers.	sloffa d'ors	bear puffball	Italian, northern	Trento area, Trentino, northern Italy	size	[74]
*Briza media* L.	*Briza media* L.	karukaer	bear oats	Estonian		inedibility	Kalle and Sõukand unpublished
*Bromus arvensis* L.	*Bromus arvensis* L.	kahrukaar	bear oat	Estonian	Ote	inedibility	[58]
*Bromus hordeaceus* L.	*Bromus hordeaceus* L.	karukaer	bear oats	Estonian	Kuu	inedibility	[58]
*Bromus secalinus* L.	*Bromus secalinus* L.	karukaer, karukaur, karukaar, karukaeras, karukael, karukaerad, kahrukaar	bear oats	Estonian	general	inedibility	[58, 73, 174]
*Bromus secalinus* L.	*Bromus secalinus* L.	karhunkaatar	bear oat	Izhorian		inedibility	[56]
Bryophyta	moss	мeдвeдяник	bear plant	Central Russian	Tver	surface?	[57]
Bryophyta	moss	мeдвeжaтник	bear plant	Northern Russian	Karelia, Novgorod region	surface?	[141]
Bryophyta	moss	мeдвeжий мox	bear moss	Northern Russian	Karelia	surface?	[141]
Bryophyta	moss	мeдвeжник	bear plant	Northern Russian	Karelia	surface?	[57]
*Bryum* spp.	*Bryum* ssp	karusammal	bear moss	Estonian		folk etymology	[56]
*Cactaceae* spp.	cactus	мeдвeжья лaпa	bear paw	Russian	Novosibirsk	shape (thorny)	[57]
*Calla palustris* L.	*Calla palustris*	мeдвeжья лaпкa	bear little paw	Belarussian	Smol.	shape	[157]
*Calla palustris* L.	*Calla palustris* L.	мeдвeжьи лaпы	bear paws	Northern Russian	Kostroma	shape?	[57]
*Calla palustris* L.	*Calla palustris*	bjønnblekker	bear leaves	Norwegian	southern Norway	shape?	[80]
*Caltha palustris* L.	*Caltha palustris* L.	мядзвeжaя лaпкa	bear little paw	Belarussian	Smol., Gomel.	shape	[79]
*Caltha palustris* L.	*Caltha palustris* L.	karukoll	bear ogre	Estonian	Hlj	folk etymology	[58]
*Calystegia sepium* (L.) R. Br.	*Calystegia sepium* L.	bearbind	bearbind	Eng. Southern	Kent, Surrey, Middlesex, Buckinghamshire, Hertfordshire, Herefordshire	folk etymology	[45]
*Calystegia sepium* (L.) R. Br.	*Calystegia sepium* (L.) R.Br.	karhunköynnös	bear garland	Finnish	Hiit	?	[82]
*Campanula glomerata* L.	*Campanula glomerata* L.	karukübar	bear hat	Estonian	Ksi, Pal	folk etymology	[58]
*Campanula glomerata* L.	*Campanula glomerata* L.	karukatel	bear kettle	Estonian	Pal	folk etymology	[58]
*Campanula patula* L.	*Campanula patula* L.	karukellad	bear bells	Estonian	Mar	folk etymology	[58]
*Campanula persicifolia* L.	*Campanula persicifolia* L.	karukell	bear bell	Estonian	Pal	folk etymology	[58]
*Campanula persicifolia* L.	*Campanula persicifolia* L.	karukatlad	bear kettle	Estonian	Jõe	folk etymology	[58]
*Campanula rotundifolia* L.	*Campanula rotundifolia* L.	karukell	bear bell	Estonian	Pal	folk etymology	[58]
*Campanula* spp.	*Campanula* spp	karukellad	bear bells	Estonian	Lai	folk etymology	[58]
*Campanula* spp.	*Campanula* spp	karukatlad	bear kettle	Estonian	Plt	folk etymology	[58]
*Capsella bursa-pastoris* (L.) Medik.	*Capsella bursa pastoris* Moench	мeчкaнa дупкa	bear hole	Bulgarian	Goce Delchev	?	[85]
*Capsella bursa-pastoris* (L.) Medik.	*Capsella bursa pastoris* L.	мeчкинa дупкa	bear hole	Macedonian	Nevrokop region	?	[179]
*Carduus crispus* L.	*Carduus crispus* L.	karuohakas, karuuhakas, karuohtja	bear thistle, bear thorn	Estonian	general	size, shape	[58]
*Carduus personata* (L.) Jacq.	*Carduus personatus*	medvesaláta	bear’s lattice	Hungarian	Gyimes (Transylvania)	bear food	[97, 213]
*Carduus* spp.	*Carduus* ssp.	karuohakad, karuohtjad	bear thistle, bear thorn	Estonian		size, shape	[64]
*Carex davalliana* Sm.	*Carex davalliana* Sm.	karulakk	bear mane	Estonian	Mih, Lih, Kse, Han, Var, Tõs	shape	[58, 174]
*Carlina acanthifolia* All.	*Carlina acanthifolia* All. (incl. subsp. utzka (Hacq.) Meusel & Kästner)	rapa d'orse	bear turnip	Italian, southern	Scanno area (L'Aquila), Abruzzo, central Italy	bear food?	[90]
*Carlina vulgaris* L.	*Carlina vulgaris* L.	karune ohak	beary (hairy) thistle	Estonian	Phl	shape	[58]
*Caucalis platycarpos* L.	*Caucalis* lappula	medvetalp	bear’s paw	Hungarian	Moldva (East-Romania)	?	[78]
*Centaurea jacea* L.	*Centaurea jacea* L.	karukellad	bear bells	Estonian	Lüg	surface	[58]
*Centaurea phrygia* L.	*Centaurea phrygia* L.	мeдвeдник	bear plant	Central Russian	Nizhni Novgorod.	shape?	[57]
*Centaurea scabiosa* L.	*Centaurea scabiosa* L.	мeдвeжья лaпa	bear paw	Central Russian	Pskov	shape	[57]
*Cetraria islandica* (L.) Ach.	*Cetraria islandica* (L.) Ach.	karusammal	bear moss	Estonian		folk etymology	[64]
*Cetraria islandica* (L.) Ach.	*Cetraria isl.*	Bärengeist	bear brandy	German, non specified	not specified	?	[65]
*Chaerophyllum aromaticum* L.	*Chärophyllum arom.*	Bärengiersch	bear ground elder	German, non specified	not specified	bear food?	[65]
*Chamerion angustifolium* (L.) Holub	*Epilobium angustifolium* L.	karukanep	bear cannabis	Estonian	Vil	folk etymology	[58]
*Chamerion angustifolium* (L.) Holub	*Epilobium angustifolium*	boelt’aajjankraesie	bear grass	South Saami		?	[148]
*Chimaphila umbellata* (L.) W.P.C. Barton	*Chimaphila umbellata* (L.) W.P.C.Barton	karhunkukka	bear flower	Finnish	Holl	?	[82]
*Chimaphila umbellata* (L.) W.P.C. Barton	*Chimaphila umbellata* (L.) Nutt.	мeдвeжьи ушки	bear little ears	Russian	Middle Ob region	shape?	[101]
*Cicerbita alpina* (L.) Wallr.	*Cicerbita alpina* (L.) Wallr.	radicio de l'urs	bear chicory	Italian, northern	Trentino and Friuli regions, north-eastern Italy	bear food	Pieroni unpublished
*Cicerbita alpina* (L.) Wallr.	*Lactuca alpina*	björnkål	bear cabbage	Swedish	Lappland, Ångermanland	bear food	[155]
*Cicerbita alpina* (L.) Wallr.	*Lactuca alpina* = *Cicerbita alpina*	björntorta	bear plant	Swedish	Dalarna	bear food	[155]
*Cicerbita alpina* (L.) Wallr.	*Cicerbita alpina*	björnkål	bear cabbage	Swedish	Lappland, Ångermanland	bear food?	[155]
*Cicerbita alpina* (L.) Wallr.	*Cicerbita alpina*	björngräs	bear grass	Swedish	Lappland, Västerbotten+H295	bear food?	[154, 155]
*Cirsium acaule* (L.) A.A.Weber ex Wigg.	*Cirsium acaule* (L.) A.A.Weber ex Wigg.	karuohakas	bear thistle, bear thorn	Estonian	Mih	shape	[58]
*Cirsium acaule* (L.) A.A.Weber ex Wigg.	*Cirsium acaule* (L.) A.A.Weber ex Wigg.	karuuhakad	bear thistle, bear thorn	Estonian	Hag, Jür, Kos, Mar	shape	[58]
*Cirsium arvense* (L.) Scop.	*Cirsium spp. arvense* (L.) Scop.	karuohakad, karused uhakad, karuuhak, karuohtjas, karuõhakas	bear thistle, bear thorn	Estonian	general	shape	[58, 173]
*Cirsium erisithales* (Jacq.) Scop.	*Cirsium erisithales* (Jacq.) Scop.	talpe d'ors salvadie	wild bear poplar	Friulian	Friuli, north-eastern Italy	bear food?	[74]
*Cirsium erisithales* (Jacq.) Scop.	*Cirsium erisithales*	medvesaláta	bear’s lattice	Hungarian	Gyimes (Transylvania)	bear food	[97, 213]
*Cirsium helenioides* (L.) Hill	*Cirsium heterophyllum* L.	bjønnetunge	bear tongue	Norwegian	Oppland	?	[80]
*Cirsium helenioides* (L.) Hill	*Cirsium heterophyllum* L.	bjønnskor	bear shoes	Norwegian	Fitjar	?	[80]
*Cirsium helenioides* (L.) Hill	*Cirsium heterophyllum* L.	bjønnehatt	bear hat	Norwegian		?	[80]
*Cirsium oleraceum* (L.) Scop.	*Cirsium oleraceum* Scop.	мeдвeжник бoлoтный	swamp bear plant	Central Russian	Pskov	shape, surface	[57]
*Cirsium oleraceum* (L.) Scop.	*Cirsium oleraceum* (L.) Scop.	karukapsad	bear cabbage	Estonian		shape	[201]
*Cirsium oleraceum* (L.) Scop.	*Cirsium oleraceum* (L.) Scop.	karuohakas, karuuhak, karuohtja, karuohass, kahruohass	bear thistle, bear thorn	Estonian	general	shape	[58]
*Cirsium oleraceum* (L.) Scop.	*Cirsium oleraceum* (L.) Scop.	talpe d'ors	bear poplar	Friulian	Friuli, north-eastern Italy	bear food?	[74]
*Cirsium oleraceum* (L.) Scop.	*Cirsium oleraceum*	medvesaláta	bear’s lattice	Hungarian	Gyimes (Transylvania)	bear food	[97, 213]
*Cirsium palustre* (L.) Scop.	*Cirsium palustre* (L.) Coss. ex Scop.	karuuhak, karuohakas, karuohtjas, niidu-karuohtja, kahruohtja, etc	bear thistle, bear thorn	Estonian	general	shape	[58]
*Cirsium rivulare* (Jacq.) All.	*Cirsium rivulare*	medvesaláta	bear’s lattice	Hungarian	Gyimes (Transylvania)	bear food	[97, 213]
*Cirsium* spp.	*Cirsium* sp.	ayïwbastiken	bear’s head	Kazakh	Xinjiang	shape?	[6]
*Cirsium* spp.	*Cirsium*	björnborst	bear bristle	Swedish	Jämtland	shape	[155]
*Cirsium* spp.	*Cirsium*	björntistel	bear thistle	Swedish	Nyland	shape	[155]
*Cirsium vulgare* (Savi) Ten.	*Cirsium vulgare* (Savi) Ten.	karuohakas, karuuhal, karuohtjas, karuuhtke, karuohe, karuuhakas, karuokas, kahro ohhak, kahru-uhak, etc	bear thistle, bear thorn	Estonian	general	shape	[58, 173]
*Cladina stellaris* (Opiz) Brodo	*Cladina stellaris* (Opiz) Pouzar & Vězda, 1971	karusammal	bear moss	Estonian		folk etymology	[56]
*Clavaria arbuscula* Scop.	*Clavaria arbuscula*	medvedove grive	bear mane	Slovenian		shape?	[84]
*Clavaria botrytis* Pers.	*Clavaria botrytis*	medvedje šapke	bear little paws	Slovenian		shape?	[84]
*Clavaria coralloides* L.	*Clavaria coralloides* L.	мeчa cтъпкa	bear step	Bulgarian	Omurtag	shape, size	[85]
*Clavaria flava* Schaeff.	*Clavaria flava*	мeчja шaпa	bear paw	Serbo-Croat		shape?	[84]
*Clavaria* spp.	*Clavaria*	Bärenpfote	bear paw	German, non specified	not specified	shape?	[65]
*Clavaria* spp.	*Clavaria*	roggene Bärenpratze	?	German, non specified	not specified	shape?	[65]
*Clavaria* spp.	*Clavaria*	Bärentatze	bear paw	German, non specified	not specified	shape?	[65]
*Claviceps purpurea* (Fr.) Tul.	*Claviceps purpurea*	мeдвeжий нoгoть	bear fingernail	Northern Russian	Karelia	colour, shape	[141]
*Clematis* spp.	*Clematis* spp. ?	aussara, autsàra, auttsàra, aursàra, attsara; ussarèddha	little female bear	Sardinian	Ogliastra, eastern Sardinia	?	[142]
*Clematis vitalba* L.	*Clematis vitalba* L.	bearbind	bearbind	Eng. Southern	Kent	folk etymology	[46]
*Colchicum autumnale* L.	*Colchicum aut.*	Bärenklachel	?	German, non specified	not specified	?	[65]
*Colchicum cilicicum* (Boiss.) Dammer	*Colchicum cilicicum* (Boiss.) Dammer	ayı çiğdemi	bear colchicum	Turkish	S Anatolia	size	[146]
*Colchicum cilicicum* (Boiss.) Dammer	*Colchicum cilicicum* (Boiss.) Dammer	ayı çiğdemi	bear colchicum	Turkish	Gaziantep (SE Anatolia)	size	[242]
*Comarum palustre* L.	*Potentilla palustris* (L.) Scop.	karhunkämmen	bear palm	Finnish	Pielav	?	[82]
*Comarum palustre* L.	*Potentilla palustris* (L.) Scop.	karhunmansikka	bear strawberry	Finnish	Keit	inedibility	[82]
*Comarum palustre* L.	*Comarum palustre*	björnklo	bear claw	Swedish	Jämtland	shape	[155]
*Conium maculatum* L.	*Conium maculatum*	мeдвeжья дудкa	bear pipe	Russian	Ural	wild, size	[181]
*Consolida regalis* Gray	*Delphinium consolida* L.	мeчини oчи	bear eys	Bulgarian	Bosilegrad (Serb.)	fruit shape and surface	[86]
*Convallaria* spp.	*Convallaria* L.	мeдвeжьe уxo	bear ear	Northern Russian	Olonets	shape?	[57]
*Convolvulus arvensis* L.	*Convolvulus arvensis* L.	bearbind	bearbind	Eng. Northern	Yorkshire	folk etymology	[46]
*Cornus alba* L.	*Svida alba* (L.) Opiz.	мeдвeжья ягoдa	bear berry	Russian	Middle Ob region	inedibility	[101]
*Cornus suecica* L.	*Cornus suecica* L.	karhunmarja	bear berry	Finnish	Pielav	folk etymology	[82]
*Cornus suecica* L.	*Cornus suecica* L.	karhunpuola	bear lingonberry	Finnish	Palt	folk etymology	[82]
*Cornus suecica* L.	*Cornus suecica*	björnebär	bear berry	Norwegian	Östfold, Akershus, Hedmark, Telemark, Sogn Og Fjordane, Möre Og Romsdal, Sör-Tröndelag	?	[155]
*Cornus suecica* L.	*Cornus suecica* L.	bjørnebær	bear berry	Norwegian	Askim, Southern Høland, Southern Odal; Hjartadal, Aurland, Ytterøy	?	[80]
*Corylus colurna* L.	*Coryllus colurna* L.	Lejthi arushe	bear hazelnut	Albanian	Kosovo	inedibility	[95]
*Corylus colurna* L.	*Corylus colurna* L.	мeчa лecкa	bear hazelnut tree	Bulgarian		shape	[86]
*Corylus colurna* L.	*Corylus colurna*	мeдвjeђa лиjecкa	bear hazel	Serbo-Croat		size?	[84]
*Corylus colurna* L.	*Corylus colurna*	мeчja лecкa	bear hazel	Serbo-Croat		size?	[84]
*Corylus colurna* L.	*Corylus colurna* L.	ayı fındığı	bear hazelnut	Turkish	Anatolia	size	[93]
*Cota tinctoria* (L.) J. Gay	*Anthemis tinctoria* L.	kahro karri kakra, karro karri kakra	bear daisy	Estonian		surface	[173, 174]
*Cota tinctoria* (L.) J. Gay	*Anthemis tinctoria* L.	päiväkarud	day's bears	Estonian	Jõh	surface	[58]
*Crataegus monogyna* Jacq.	*Crataegus monogyna* Jacq.	мeчи ябълки	bear apples	Bulgarian		bear food	[86]
*Crataegus monogyna* Jacq.	*Crataegus oxyac.*	Bärenbrot	bear bread	German, non specified	not specified	bear food	[65]
*Crataegus monogyna* Jacq.	*Crataegus oxyacantha* L.	pomatoi d'ors	bear fruit	Italian, northern	Giudicarie area, Trentino, north-eatern Italy	bear food?	[115]
*Crataegus monogyna* Jacq.	*Crataegus oxyacantha* L.	pomele de ors	bear fruit	Italian, northern	Lavis, Trentino, north-eastern Italy	bear food?	[115]
*Crataegus monogyna* Jacq.	*Crataegus oxyacantha* L.	spin d'ors	bear thorn	Italian, northern	Belluno area, Veneto, northern Italy	wild	[74]
*Crataegus monogyna* Jacq.	*Crataegus oxyacantha* L.	pan d'orsèr; pan d'ors	bear bread	Italian, northern	Belluno area, Veneto and Trentino, northern Italy	bear food	[74, 115]
*Crataegus monogyna* Jacq.	*Crataegus oxyacantha*	medvêdnik	bear plant	Slovenian		status-?	[149]
*Crataegus monogyna* Jacq.	*Crataegus oxyacantha*	medvednek	bear plant	Slovenian		status-?	[84, 89]
*Crataegus monogyna* Jacq.	*Crataegus oxyacantha*	medvedova hrušca	bear pear	Slovenian		status-?	[84, 89]
*Crataegus monogyna* Jacq.	*Crataegus oxyacantha*	medvedova hrušica	bear pear	Slovenian		status-?	[84, 89]
*Crataegus monogyna* Jacq.	*Crataegus oxyacantha*	medvedove hruške	bear pears	Slovenian		status-?	[84, 89]
*Crataegus* spp.	*Crataegus* spp.	pirigli de li ursi	little bear pear	Italian, southern	Lucoli and Marsica souroundings (L'Aquila), Abruzzo, central Italy	bear food?	([90], Cianfaglione unpublished)
*Cyclamen cilicium* Boiss. & Heldr.	*Cyclamen cilicium* Boiss. & Heldr.	ayı pancarı	bear beet	Turkish	Karaman (S Anatolia)	size, status- (tuber)	[92]
*Cyclamen hederifolium* Aiton	*Cyclamen hederifolium* Aiton	orecchia d'orso	bear ear	Italian, central	Tuscany, central Italy	shape	[74]
*Cynoglossum creticum* Mill.	*Cynoglossum creticum* Mill.	ayı kulağı	bear ear	Turkish	Anatolia	shape	[92]
*Cynoglossum officinale* L.	*Cynoglossum officinale* L.	мeдвeжe вушкo	bear little ear	Ukrainian, Volhynian	Vinnitsa	shape?	[180, 203]
*Cypripedium calceolus* L.	*Cypripedium calceolus* L.	karu-ummisking	bear shoe with closed toe	Estonian	Mih	folk etymology	[58]
*Dactylis glomerata* L.	*Dactylis glom.*	Bärentappe	bear paw	German, non specified	not specified	shape and surface?	[65]
*Dactylorhiza maculata* (L.) Soó	*Orchis maculata* L.	scrinfe dl'óurs	bear feet	Ladin	Livinallongo, Veneto, north-eastern Italy	?	[115]
*Daphne mezereum* L.	*Daphne mezereum*	meškalunkis	bear meadow	Lithuanian	Lithuania	dangerousness, bear food	[192]
*Daphne mezereum* L.	*Daphne mezereum*	мeдвeжья ягoдa	bear berry	Northern Russian	Karelia	dangerousness (toxic)	[141]
*Daphne mezereum* L.	*Daphne mezereum*	мeдвeжник	bear plant	Northern Russian	Karelia	dangerousness (toxic)	[141]
*Daphne mezereum* L.	*Daphne mezereum*	мeчja ликa	bear bast	Serbo-Croat		dangerousness (toxic)?	[84]
*Dasypyrum villosum* (L.) P. Candargy	*Dasypyrum villosum* (L.) Borbás	granë dell'urse	bear wheat	Italian, southern	Rocca Pia (L'Aquila), Abruzzo, central Italy	bear food?	[90]
*Delphinium* spp.	*Delphinium*	мeдвиje уxo	bear ear	Serbo-Croat		shape?	[84]
*Deschampsia cespitosa* (L.) P. Beauv.	*Deschampsia cespitosa*	björngägal	bear grass	Swedish	Västerbotten	?	[154]
*Deschampsia cespitosa* (L.) P. Beauv.	*Aira caespito*	björnjägal	bear’s grass	Swedish	Northern Sweden	?	[224]
*Digitalis ferruginea* L.	*Digitalis ferruginea* ssp. schischkinii	ayı mısırı	bear corn	Turkish	Trabzon (NE Anatolia)	shape	[227]
*Digitalis grandiflora* Mill.	*Digitalis grandiflora* (= *Digitalis ambigua*)	meškos taurė	bear cup	Lithuanian	Lithuania	dangerousness, bear food	[87, 96, 191]
*Digitalis purpurea* L.	*Digitalis purpurea*	björneblomst	bear flower	Norwegian	Rogaland	?	[155]
*Digitalis purpurea* L.	*Digitalis purpurea*	bjørneblomst	bear flower	Norwegian	Sauda	?	[80]
*Digitalis purpurea* L.	*Digitalis purpurea*	bjønnehanskje	bear glove	Norwegian	Sunnfjord	?	[80]
*Dioscorea communis* (L.) Caddick & Wilkin	*Dioscorea communis* (L.) Caddick & Wilkin	ayı baklası	bear vetch	Turkish	Bartın (NW Anatolia)	shape	[227]
*Drimia maritima* (L.) Stearn	*Drimia maritima* (L.) Stearn	ayı soğanı	bear bulb	Turkish	Muğla (SW Anatolia)	size	[147]
*Drimia maritima* (L.) Stearn	*Urginea maritima*	ayıkulağı	bear ear	Turkish	S Anatolia	shape	[226]
*Dryopteris filix-mas* (L.) Schott	*Aspidium fil.-mas*	Bärenkraut	bear herb	German, non specified	not specified	?	[65]
*Dryopteris filix-mas* (L.) Schott	*Aspidium fil.-mas*	Bärenwurz	bear root	German, non specified	not specified	?	[65]
*Dryopteris filix-mas* (L.) Schott	*Dryopteris filix-mas* (L.) Schott	ayıdöşeği	bear mattress	Turkish	Hasanbeyli-Adana (S. Anatolia)	shape	[198]
*Dryopteris* spp.	*Dryopteris* spp.	karukaatet	bear cover or isolation?	Ingrian		?	[56]
*Dryopteris* spp.	*Dryopteris* spp.	karukaattet	bear cover or isolation?	Ingrian		?	[56]
*Dryopteris* spp.	*Dryopteris* spp	karhunkaaded	bear cover or isolation?	Izhorian		?	[56]
*Echium italicum* L.	*Echium italicum*	ayı kulağı	bear ear	Turkish	Muğla (SW Anatolia)	shape	[227]
*Echium vulgare* L.	*Echium vulgare* L.	karukellad	bear bells	Estonian	Ksi	shape	[58]
*Echium vulgare* L.	*Echium vulgare* L.	karulill	bear flower	Estonian	PJg	shape	[58]
*Echium vulgare* L.	*Echium vulgare* L.	karukäpp	bear paw	Estonian	Kaa, Vil	shape	[58]
*Echium vulgare* L.	*Echium vulgare* L.	karuhänd	bear tail	Estonian	Aud, Mih, Pär, Tõs	shape	[58]
*Echium vulgare* L.	*Echium vulgare* L.	karuohakas	bear thistle	Estonian	Kei	shape	[58]
*Echium vulgare* L.	*Echium vulgare*	medvedov rep	bear tail	Slovenian		shape?	[4, 84]
*Empetrum nigrum* L.	*Empetrum nigrum* L.	karuleisik	bear bearberry	Estonian	Vig	folk etymology	[58]
*Empetrum nigrum* L.	*Empetrum nigrum* L.	karumarjad, kahro marja	bears's berry	Estonian	Kam	folk etymology	[58, 173]
*Empetrum nigrum* L.	*Empetrum nigrum* L.	karumustikad, karumustikas	bear blackberry	Estonian	Vig	folk etymology	([58], Sõukand and Kalle unpublished)
*Empetrum nigrum* L.	*Empetrum nigrum* L.	karhunmarja, karhunmarjoja	bear berry	Finnish	Räis, Sakk	folk etymology	[55, 82]
*Empetrum nigrum* L.	*Empetrum nigrum* L.	karhun mustikka	bear blackberry	Finnish	Sakk	folk etymology	[55, 82]
*Empetrum nigrum* L.	*Empetrum nigrum*	meškuogė	bear berries	Lithuanian	Lithuania	bear food	[87, 96, 191]
*Empetrum nigrum* L.	*Empetrum nigrum* L.	bjønnebær	bear berry	Norwegian	southern Norway	bear food?	[80]
*Empetrum nigrum* L.	*Empetrum nigrum* L.	мeдвeжьи ягoды	bear berries	Russian	Tobolsk area	wild? status-?	[218]
*Empetrum nigrum* L.	*Empetrum nigrum*	björnbär	bear berry	Swedish	Dalarna	bear food?	[155]
*Empetrum nigrum* L.	*Empetrum hermafroditum* Hagerup	björnbär	bear berries	Swedish	northern Sweden	status-	[54]
*Enkianthus* spp.	*Enxyanthus*	medvjedica	she-bear	Croatian	no location	?, surface?	[126, 216]
*Epilobium hirsutum* L.	*Epilobium hirsutum* L.	kahruliht	bear leaves	Estonian	Har	surface	[58]
*Equisetum arvense* L.	*Equisetum arvense* L.	мeчe вpeтeнo	bear spindle	Bulgarian		shape of spike	[86]
*Equisetum arvense* L.	*Equisetum arvense* L.	kahrutilk	bear drop	Estonian	Se	folk etymology	[58]
*Equisetum arvense* L.	*Equisetum arvense*	medvebajusz	bear’s moustache	Hungarian	Csinód (Transylvania)	shape	Frendl and Papp unpublished
*Equisetum arvense* L.	*Equisetum arvense*	medveszakála, medveszakálla	bear’s beard	Hungarian	Moldva (East-Romania)	shape	[78]
*Equisetum arvense* L.	*Equisetum arvense*	medvefarka	bear’s tail	Hungarian	Moldva (East-Romania)	shape	[78]
*Equisetum arvense* L.	*Equisetum arvense* L.	meškabarzdė	bear beard	Lithuanian	Lithuania	shape, bear food	[177]
*Equisetum arvense* L.	*Equisetum arvense*	bjornbar	bear berry	Swedish	Ångermanland	?	[220]
*Equisetum* spp.	*Equisetum* L.	мeчe вpeтeнo	bear spindle	Bulgarian		shape of spike	[86]
*Erica herbacea* L.	*Erica herbacea* L.	Bar i ariut	bear grass	Albanian	Albania-Kolonjë	?	[136]
*Eryngium planum* L.	*Eryngium planum* L.	вeдмeдиця	she-bear	Ukrainian, Slobozhanian		shape?	[180, 219]
*Euonymus verrucosus* Scop.	*Evonymus verrucosa* Scop.	мeдвeжник	bear plant	Central Russian	Nizhni Novgorod	?	[57]
*Euphorbia cyparissias* L.	*Euphorbia cyp.*	Bärenmutter-gras	bear mother grass	German, non specified	not specified	folk etymology?	[65]
*Euphorbia myrsinites* L.	*Euphorbia myrsinites* L.	мeчe pунo	bear fleece	Bulgarian	Stara Zagora area	surface, shape	[86]
*Fallopia convolvulus* (L.) Á.Löve	*Polygonum convolvulus* L.	bearbind	bearbind	Eng. Midlands	Staffordshire	folk etymology	[45]
*Filipendula ulmaria* (L.) Maxim.	*Filipendula ulm.*	Barenmutter-strauss	bear mother bouquet	German, non specified	not specified	folk etymology?	[65]
*Filipendula ulmaria* (L.) Maxim.	*Spiraea (= Filipendula) ulmaria*	medvejno latje	bear stalks	Slovenian		bear food?	[89]
*Filipendula ulmaria* (L.) Maxim.	*Spiraea filipendula*	medvejka	bear plant	Slovenian		bear food?	[89]
*Foeniculum vulgare* Mill.	*Foenicullum vulgare* Miller.	meškakrapis	bear dill	Lithuanian	Lithuania	surface, bear food	[66, 87, 199]
*Fragaria vesca* L.	*Fragaria vesca* L.	мeчи ягoди	bear strawberries	Bulgarian	Ihtiman area	bear food	[86]
*Galium aparine* L.	*Galium aparine* L.	karune virn, karuvirn, karune virnirohi, karuvirn	bear pile	Estonian	western Estonia and Isles	surface	[58]
*Galium aparine* L.	*Galium aparine* L.	karuserohud	beary grass	Estonian	Mih	surface	[58]
*Galium boreale* L.	*Galium boreale* L.	karukaer	bear oats	Estonian	Äks	folk etymology	[58]
*Galium verum* L.	*Galium verum*	Bärenkraut	bear herb	German, non specified	not specified	?	[65]
*Galium verum* L.	*Galium verum*	мeчje увo	bear ear	Serbo-Croat		?	[84]
*Gentiana pneumonanthe* L.	*Gentiana pneumonanthe*	björnblomma	bear flower	Swedish	Småland	status-	[155]
*Geranium phaeum* L.	*Gaillardia, Geranium phaeum*	Bärenäugelchen	little bear eyes	German, non specified	not specified	shape	[65]
Geranium pratense L.	Geranium pratense L.	мeдвeжья лaпa	bear paw	Central Russian	Vladimir	shape?	[57]
*Geranium robertianum* L.	*Geranium robertianum*	bjødnablomster	bear flower	Norwegian	Balestrand	?	[80]
*Geum rivale* L.	*Geum rivale* L.	karulilled	bear flowers	Estonian	Kei	surface	[58]
*Geum rivale* L.	*Geum rivale* L.	karusukapaelad, karusäärepaelad	bear garters, bear shank's ribbon	Estonian	JMd, Rap, Tür	surface	[58]
*Geum rivale* L.	*Geum rivale* L.	karukäpp	bear paw	Estonian	North Estonia	surface	[58]
*Geum rivale* L.	*Geum rivale* L.	karumaasikad, karumarjad, karumulukas	bear strawberries, bear berries	Estonian	LNg	surface	[58]
*Geum rivale* L.	*Geum rivale* L.	karukolluksed	bears's bells	Estonian	Kei	surface	[58]
*Geum rivale* L.	*Geum rivale* L.	karukuljused	bears's bells	Estonian	Pal	surface	[58]
*Geum rivale* L.	*Geum rivale* L.	karukell, pruun karukell	bear bell, brown bear bell	Estonian	North Estonia, Hää, Khk, Saa	surface	([73, 58], Sõukand and Kalle unpublished)
*Geum rivale* L.	*Geum rivale* L.	karhunkukka, karunkukka	bear flower	Ingrian		surface	[56]
*Geum rivale* L.	*Geum rivale* L.	karhunkukka	bear flower	Izhorian		surface	[56]
*Geum rivale* L.	*Geum rivale*	björntobak	bear tobacco	Swedish	Jämtland	status-	[155]
*Geum urbanum* L.	*Geum urbanum*	мeчиja cтoпa	bear foot	Serbo-Croat		shape?	[84]
*Glycyrrhiza glabra* L.	*Glycyrrhiza glabra*	ayı kulağı	bear ear	Turkish	Anatolia	shape	[226]
*Glycyrrhiza glabra* L.	*Glycyrrhiza glabra*	ayıkulaği	bear’s ear	Turkish		shape	[6]
GRASSES	*grass straws left after mowing grass*	björnhår	bear hair	Swedish	Western Finland	shape	[139]
*Gymnadenia conopsea* (L.) R. Br.	*Gymnadenia conopsea* (L.) R.Br.	karukäpp	bear paw	Estonian	LNg	?	[58]
*Gymnocarpium dryopteris* (L.) Newman	*Gymnocarpium dryopteris* (L.) Newman	мeчa пaпpaт	bear fern	Bulgarian		shape, size	[86]
*Gyromitra esculenta* (Pers.) Fr.	*Gyromitra esculenta* (Pers. ex Pers.) Fr.	okš-šõrməz	bear septum	Livonian		shape?	[81]
*Helleborus foetidus* L.	*Helleborus foetidus* L.	he-barfoot	he-bear foot	Eng. Midland	Warwickshire	shape	[45]
*Helleborus foetidus* L.	*Helleborus foetidus* L.	bear's foot	bear foot	Eng. Southern, Western, Midland, Northern	Dorset, Somerset, Wiltshire, Warwickshire, Worcestershire, Yorkshire	shape	[45]
*Helleborus foetidus* L.	*Helleborus foet*	Bärenklau, echte, italienische, Welsche	bear claw, true, Italian, ?	German, non specified	not specified	shape?	[65]
*Helleborus foetidus* L.	*Helleborus foet.*	Bärenfuss	bear foot	German, non specified	not specified	shape?	[65]
*Helleborus viridis* L.	*Helleborus vir.*	Bärenwurz	bear root	German, non specified	not specified	bear food?	[65]
*Helleborus viridis* subsp. occidentalis (Reut.) Schiffn.	*Helleborus viridis* L. ssp. occidentalis	she-barfoot	she-bear foot	Eng. Midland	Warwickshire	shape	[45]
*Helleborus viridis* subsp. occidentalis (Reut.) Schiffn.	*Helleborus viridis* L. ssp. occidentalis	bear's foot	bear foot	Eng. Western	Gloucestershire	shape	[45]
*Heracleum mantegazzianum* Sommier & Levier	*Heracleum mantegazzianum* Som. et Levier	karuputk	bear pipe	Estonian	general	folk etymology	Kalle and Sõukand unpublished
*Heracleum sosnowskyi* Manden.	*Heracleum sosnowskyi* Manden.	karuputk	bear pipe	Estonian	general	folk etymology	Sõukand and Kalle unpublished
*Heracleum sphondylium* L.	*Heracleum sphondylium*	ayïpänġäsi	bear claw	Azeri		Latin calque	[171]
*Heracleum sphondylium* L.	*Heracleum sphondylium*	ayïwtabanï	bear sole	Bashkir		Latin calque	[171]
*Heracleum sphondylium* L.	*Heracleum ternatum Velen.*	мeчa cтъпкa	bear step	Bulgarian	Teteven area	shape, surface	[86]
*Heracleum sphondylium* L.	*Heracleum sphondylium* L.	мeчи уши	bear ears	Bulgarian		shape, surface	[86]
*Heracleum sphondylium* L.	*Heracleum ternatum Velen.*	мeчи уши	bear ears	Bulgarian		shape, surface	[86]
*Heracleum sphondylium* L.	*Heracleum sphondylium*	upauri	bear foot	Chuvash		shape	[171]
*Heracleum sphondylium* L.	*Heracleum sphondylium* L.	medvidovina	bear plant	Croatian	Devčićim near Krasna	Latin calque	[126]
*Heracleum sphondylium* L.	*Heracleum sphondylium* L.	medviđa šapa	bear paw	Croatian	Krasna, Lica	Latin calque	[126]
*Heracleum sphondylium* L.	*Heracleum sphondylium* L.	taca medvedova	bear paw	Croatian		Latin calque	[126, 138]
*Heracleum sphondylium* L.	*Heracleum sphondylium* L.	medvjeđi dlan	bear palm	Croatian	no location	Latin calque	[126, 230, 231, 228]
*Heracleum sphondylium* L.	*Heracleum sphondylium* L.	stopa medvidnja	bear foot	Croatian		Latin calque	[126, 138, 239]
*Heracleum sphondylium* L.	*Heracleum sphondylium* L.	stopa medviđa	bear foot	Croatian		Latin calque	[126, 138, 239]
*Heracleum sphondylium* L.	*Heracleum sphondylium* L.	stopa medvinja	bear foot	Croatian		Latin calque	[126, 138, 239]
*Heracleum sphondylium* L.	*Heracleum sphondylium* L.	stopa medvjednja	bear foot	Croatian		Latin calque	[126, 138, 239]
*Heracleum sphondylium* L.	*Heracleum sphondylium* L.	stopa medvjeđa	bear foot	Croatian		Latin calque	[126, 138, 239]
*Heracleum sphondylium* L.	*Heracleum sphondylium* L.	stopa medvjeska	bear foot	Croatian		Latin calque	[126, 138, 239]
*Heracleum sphondylium* L.	*Heracleum sphondylium* L.	medvědí pazneht	bear hoof	Czech		Latin calque	[61]
*Heracleum sphondylium* L.	*Heracleum sphondylium* L.	nedvědí paznecht	bear hoof	Czech		Latin calque	[61]
*Heracleum sphondylium* L.	*Heracleum sphondylium*	bjørnelab	bear claw	Danish	Denmark	Latin calque	[128]
*Heracleum sphondylium* L.	*Heracleum sphondylium*	bjørneklo	bear claw	Danish	Denmark	Latin calque	[187]
*Heracleum sphondylium* L.	*Heracleum sphondylium* L.	bear's breech	bear breech	Eng. Western	Somerset	surface	[45]
*Heracleum sphondylium* L.	*Heracleum sphondylium*	karhunkämmen, karhunkämmen, carhuncämmen	bear palm	Finnish		size and surface	[82]
*Heracleum sphondylium* L.	*Heracleum sphondylium* L.	talpe di ors	bear poplar	Friulian	Friuli, north-eastern Italy	status-	[74]
*Heracleum sphondylium* L.	*Heracleum sphond.*	Bärenpfote	bear paw	German, non specified	not specified	?	[65]
*Heracleum sphondylium* L.	*Heracleum sphond.*	Bärentalpe	?	German, non specified	not specified	?	[65]
*Heracleum sphondylium* L.	*Heracleum sphond.*	Bärentrappen	?	German, non specified	not specified	?	[65]
*Heracleum sphondylium* L.	*Heracleum sphond.*	Bärentrasche	?	German, non specified	not specified	?	[65]
*Heracleum sphondylium* L.	*Heracleum sphond.*	Bärenkraut	bear herb	German, non specified	not specified	bear food	[65]
*Heracleum sphondylium* L.	*Heracleum sphond.*	Bärenkümmel	bear caraway	German, non specified	not specified	bear food	[65]
*Heracleum sphondylium* L.	*Heracleum sphond.*	Bärenbletsche	?	German, non specified	not specified	shape?	[65]
*Heracleum sphondylium* L.	*Heracleum sphond.*	Bärenfuss	bear foot	German, non specified	not specified	shape?	[65]
*Heracleum sphondylium* L.	*Heracleum sphond.*	Bärenklau, deutsche, unechte	bear claw, true, Italian, ?	German, non specified	not specified	shape?	[65]
*Heracleum sphondylium* L.	*Heracleum sphond.*	Bärenlatsche	bear mountain pine	German, non specified	not specified	shape?	[65]
*Heracleum sphondylium* L.	*Heracleum sphond.*	Bärenplampe	?	German, non specified	not specified	shape?	[65]
*Heracleum sphondylium* L.	*Heracleum sphond.*	Bärenpratze	bear paw	German, non specified	not specified	shape?	[65]
*Heracleum sphondylium* L.	*Heracleum sphond.*	Bärentappe	bear paw	German, non specified	not specified	shape?	[65]
*Heracleum sphondylium* L.	*Heracleum sphond.*	Bärentatze	bear paw	German, non specified	not specified	shape?	[65]
*Heracleum sphondylium* L.	*Heracleum sphond.*	Bärenbrand	bear brandy	German, non specified	not specified	?	[65]
*Heracleum sphondylium* L.	*Heracleum sphond.*	Bärlapp	bear club moss	German, non specified	not specified	?	[65]
*Heracleum sphondylium* L.	*Heracleum sphond.*	Bärenwurz	bear root	German, non specified	not specified	bear food?	[65]
*Heracleum sphondylium* L.	*Heracleum sphondylium*	medvetalp	bear’s paw	Hungarian	Pusztina; Szigetköz; Felsőtárkány; Csíkszereda; Bácska-Bánát	shape	[78, 166–168]
*Heracleum sphondylium* L.	*Heracleum sphondylium*	medveköröm	bear’s nail	Hungarian	no data (Hungary or Transylvania)	?	[176]
*Heracleum sphondylium* L.	*Heracleum sphondylium*	medvelapu	bear’s leaf	Hungarian	no data (Hungary or Transylvania)	size	[176]
*Heracleum sphondylium* L.	*Heracleum sphondylium*	guovzzasámil	bear angelica	North Saami		?	[129]
*Heracleum sphondylium* L.	*Heracleum sphondylium* L.	nedvězie noha	bear leg	old-Czech		Latin calque	[72]
*Heracleum sphondylium* L.	*Heracleum sphondylium* L.	nedvězí pazneht	bear hoof	old-Czech		Latin calque	[72]
*Heracleum sphondylium* L.	*Heracleum sphondylium* L.	nedvězí paznoht	bear hoof	old-Czech		Latin calque	[72]
*Heracleum sphondylium* L.	*Heracleum sphondylium* L.	nedvědí pazúr	bear claw	old-Czech		Latin calque	[72]
*Heracleum sphondylium* L.	*Heracleum sphondylium* L.	nedvěnóžka	bear leg	old-Czech		Latin calque	[196]
*Heracleum sphondylium* L.	*Heracleum sphondylium* s.l.	łapa niedźwiedzia	bear paw	Polish	archival name	shape	[68, 123, 124]
*Heracleum sphondylium* L.	*Heracleum sphondylium* s.l.	niedźwiedzia noga	bear leg	Polish	archival name	shape	[122, 196, 222]
*Heracleum sphondylium* L.	*Heracleum sphondylium* L.	niedźwiedzia stopa	bear foot	Polish		shape, Latin calque	[232]
*Heracleum sphondylium* L.	*Heracleum sphondylium* L.	мeдвeжья лaпa	bear paw	Russian		Latin calque	[116]
*Heracleum sphondylium* L.	*Heracleum sphondylium* L.	мeдвeжья cтoпa тpaвa	bear foot grass	Russian		Latin calque	[116]
*Heracleum sphondylium* L.	*Heracleum sphondylium* L.	мeдвeдзoвa длaнь	bear palm	Ruthenian	Vojvodina	Latin calque?	[125]
*Heracleum sphondylium* L.	*Heracleum sphondylium* L.	лaбa мeдвeдзoвa	bear paw	Ruthenian	Vojvodina	Latin calque?	[125]
*Heracleum sphondylium* L.	*Heracleum sphondylium*	мeдвeђи длaн	bear palm	Serbo-Croat		Latin calque	[84]
*Heracleum sphondylium* L.	*Heracleum sphondylium*	мeдвидњa cтoпa	bear foot	Serbo-Croat		Latin calque	[84]
*Heracleum sphondylium* L.	*Heracleum sphondylium*	мeдвидњa cтупa	bear foot	Serbo-Croat		Latin calque	[84]
*Heracleum sphondylium* L.	*Heracleum sphondylium*	мeдвиђa cтoпa	bear foot	Serbo-Croat		Latin calque	[84]
*Heracleum sphondylium* L.	*Heracleum sphondylium*	мeдвиђa cтупa	bear foot	Serbo-Croat		Latin calque	[84]
*Heracleum sphondylium* L.	*Heracleum sphondylium*	мeдвињa cтoпa	bear foot	Serbo-Croat		Latin calque	[84]
*Heracleum sphondylium* L.	*Heracleum sphondylium*	мeдвињa cтупa	bear foot	Serbo-Croat		Latin calque	[84]
*Heracleum sphondylium* L.	*Heracleum sphondylium*	мeдвjeдњa cтoпa	bear foot	Serbo-Croat		Latin calque	[84]
*Heracleum sphondylium* L.	*Heracleum sphondylium*	мeдвjeдњa cтупa	bear foot	Serbo-Croat		Latin calque	[84]
*Heracleum sphondylium* L.	*Heracleum sphondylium*	мeдвjeђa cтoпa	bear foot	Serbo-Croat		Latin calque	[84]
*Heracleum sphondylium* L.	*Heracleum sphondylium*	мeдвjeђa cтупa	bear foot	Serbo-Croat		Latin calque	[84]
*Heracleum sphondylium* L.	*Heracleum sphondylium*	мeдвjeђи длaн	bear palm	Serbo-Croat		Latin calque	[84]
*Heracleum sphondylium* L.	*Heracleum sphondylium*	мeдвjecкa cтoпa	bear foot	Serbo-Croat		Latin calque	[84]
*Heracleum sphondylium* L.	*Heracleum sphondylium*	мeдвjecкa cтупa	bear foot	Serbo-Croat		Latin calque	[84]
*Heracleum sphondylium* L.	*Heracleum sphondylium*	мeчja шaпa	bear paw	Serbo-Croat		Latin calque	[84]
*Heracleum sphondylium* L.	*Heracleum sphondylium* L.	medvedi noha	bear leg	Slovak	Slovakia	Latin calque	[62]
*Heracleum sphondylium* L.	*Heracleum sphondylium* L.	nedvědínoga	bear leg	Slovak	Slovakia	Latin calque	[62]
*Heracleum sphondylium* L.	*Heracleum sphondylium* L.	medvedova taca	bear paw	Slovenian		Latin calque	[89, 144]
*Heracleum sphondylium* L.	*Heracleum sphondylium* L.	medvedove tace	bear paws	Slovenian		Latin calque	[89]
*Heracleum sphondylium* L.	*Heracleum sphondylium* L.	medvedova dlan	bear palm	Slovenian		Latin calque	[84, 89]
*Heracleum sphondylium* L.	*Heracleum sphondylium* L.	barowa pazora	bear claw	Sorbian		German calque?	[76]
*Heracleum sphondylium* L.	*Heracleum sphondylium*	björnkummin	bear cummin	Swedish	Jämtland	?	[155]
*Heracleum sphondylium* L.	*Heracleum sphondylium*	björnloka	bear plant	Swedish		?	[54]
*Heracleum sphondylium* L.	*Heracleum sphondylium*	ayaghï	bear foot	Tatar		Latin calque	[171]
*Heracleum sphondylium* L.	*Heracleum sphondylium*	ayïwtabanï	bear sole	Tatar		Latin calque	[171]
*Heracleum sphondylium* L.	*Heracleum sphondylium* L.	barjaca stopa	bear foot	Upper-Sorbian		Latin calque	[63]
*Heracleum sphondylium* L.	*Heracleum sphondylium* L.	barjaca paca	bear paw	Upper-Sorbian		Latin calque	[63]
*Heracleum sphondylium* L.	*Heracleum sphondylium* L.	barjace palcy	bear fingers	Upper-Sorbian		Latin calque	[63]
*Heracleum sphondylium* L.	*Heracleum sphondylium* L.	barjace pacy	bear paws	Upper-Sorbian	Bautzen	Latin calque	[63, 121]
*Heracleum sphondylium* L.	*Heracleum sphondylium* L.	barjace pazory	bear claws	Upper-Sorbian	Bautzen	Latin calque	[63, 120, 121]
*Heracleum sphondylium* L.	*Heracleum sphondylium*	karuputkõ	bear plant	Votic		size and surface	[113]
*Heracleum sphondylium* subsp. sibiricum (L.) Simonk.	*Heracleum sibiricum* L.	karukapsalehed	bear cabbage leaves	Estonian	Vig	surface	[58]
*Heracleum sphondylium* subsp. sibiricum (L.) Simonk.	*Heracleum sibiricum* L.	karupill	bear pill	Estonian	Vll	surface	[58]
*Heracleum sphondylium* subsp. sibiricum (L.) Simonk.	*Heracleum sibiricum* L.	karuvile	bear whistle	Estonian	Käi	surface	[58]
*Heracleum sphondylium* subsp. sibiricum (L.) Simonk.	*Heracleum sibiricum* L.	karulik	like a bear	Estonian	Trt	surface	[58]
*Heracleum sphondylium* subsp. sibiricum (L.) Simonk.	*Heracleum sibiricum* L., Branca ursi	karuputk, karuputked, karuputke, karuputkes, karroputk	bear pipe	Estonian	North Estonia	surface	[58, 73, 173, 183]
*Heracleum sphondylium* subsp. sibiricum (L.) Simonk.	*Heracleum sibiricum* L.	karhunkämmen, karhun kämmen, carhun cämmen	bear palm	Finnish	Turku	size, surface	[82]
*Heracleum sphondylium* subsp. sibiricum (L.) Simonk.	*Heracleum sibiricum* L.	karhunurtti	bear herb	Finnish		surface	[82]
*Heracleum sphondylium* subsp. sibiricum (L.) Simonk.	*Heracleum sibiricum* L.	karhunputki	bear pipe	Finnish	many written records	surface	[82]
*Heracleum sphondylium* subsp. sibiricum (L.) Simonk.	*Heracleum sibiricum* L.	karunputki, karunputet	bear pipe(s)	Ingrian		surface	[56]
*Heracleum sphondylium* subsp. sibiricum (L.) Simonk.	*Heracleum sibiricum* L.	karhunputki, karuputki, karupuded	bear pipe	Izhorian		surface	[56]
*Heracleum sphondylium* subsp. sibiricum (L.) Simonk.	*Heracleum sibiricum* L.	мeдвeжья лaпa	bear paw	Russian		Latin calque	[116]
*Heracleum sphondylium* subsp. sibiricum (L.) Simonk.	*Heracleum sibiricum*	мeчja шaпa вeликa	big bear paw	Serbo-Croat		Latin calque	[84]
*Heracleum sphondylium* subsp. sibiricum (L.) Simonk.	*Heracleum sibiricum*	björnloka	bear plant	Swedish	Gästrikland, Dalarna, Västmanland	?	[155]
*Heracleum sphondylium* subsp. sibiricum (L.) Simonk.	*Heracleum sibiricum*	björnkummin	bear cummin	Swedish	Jämtland	?	[155]
*Heracleum sphondylium* subsp. sibiricum (L.) Simonk.	*Heracleum sibiricum*	björnpipa	bear pipe	Swedish	Värmland, Västmanland	?	[155]
*Heracleum sphondylium* subsp. sibiricum (L.) Simonk.	*Heracleum sibiricum* L.	karuputked, karuputki, karu-pudgõd, karu-pudged, karuputke	bear pipe	Votic		surface	[56, 172]
*Heracleum* spp.	*Heracleum* L.	мeчa cтъпкa	bear step	Bulgarian		shape, surface	[86]
*Heracleum* spp.	*Heracleum* spp.	medvedska taca	bear paw	Croatian		Latin calque	[228]
*Heracleum* spp.	*Heracleum* ssp.	karuputked	bear pipes	Estonian	general	genera name borrowed from the one species	[56, Sõukand, Kalle unpubl]
*Heracleum* spp.	*Heracleum* L. spp.	ramba de j'erse	bear claw	Italian, southern	Pietracamela (Teramo), Abruzzo, central Italy	shape	[90]
*Heracleum* spp.	*Heracleum*	björnstut	bear toot	Swedish	Jämtland	?	[155]
*Heracleum* spp.	*Heracleum ?*	björnkål	bear cabbage	Swedish	Dalarna	?	[155]
*Heracleum* spp.	*Heracleum*	björnskalle	bear skull	Swedish	Dalsland	shape	[155]
*Hieracium pilosella* L.	*Hieracium pilosella* L.	вeдмeжe вуxo	bear ear	Ukrainian, steppe dialect	Kirovograd	shape?	[215]
*Himantoglossum affine* (Boiss.) Schltr.	*Himantoglossum affine* (Boiss.) Schltr.	ayı kulağı	bear ear	Turkish	Kahramanmaraş (S Anatolia)	shape	[241]
*Himantoglossum affine* (Boiss.) Schltr.	*Himantoglossum affine* (Boiss.) Schltr.	ayı kulağı	bear ear	Turkish	Kahramanmaraş (S Anatolia)	shape	[241]
*Hordeum vulgare* L.	*Hordeum vulgare (and esp. hexastichon or tetrastichon*	bar	bear	Eng. Eastern	Suffolk	folk etymology	[45]
*Hordeum vulgare* L.	*Hordeum vulgare (and esp. hexastichon or tetrastichon*	beer	bear	Eng. Northern	Northumberland	folk etymology	[45]
*Hordeum vulgare* L.	*Hordeum vulgare (and esp. hexastichon or tetrastichon*	beir	bear	Eng. Northern	northern coutnties	folk etymology	[45]
*Hordeum vulgare* L.	*Hordeum vulgare (and esp. hexastichon or tetrastichon*	bere	bear	Eng. Northern	North Yorkshire	folk etymology	[45]
*Hordeum vulgare* L.	*Hordeum vulgare (and esp. hexastichon or tetrastichon*	bear-barley	bear-barley	Eng. Northern	Northumberland	folk etymology	[45]
*Hordeum vulgare* L.	*Hordeum vulgare (and esp. hexastichon or tetrastichon*	bear	bear	Eng. Northern, Midland, Western, Eastern	Northumberland, Yorkshire, Lincolnshire, Shropshire, Suffolk	folk etymology	[45]
*Hordeum vulgare* L.	*Hordeum polyst.*	Bärengerste	bear barley	German, non specified	not specified	bear food?	[65]
*Horminum pyrenaicum* L.	*Horminum pyr.*	Bärenknöpfe	bear buttons	German, non specified	not specified	shape?	[65]
*Huperzia selago* (L.) Bernh. ex Schrank & Mart.	*Huperzia selago* (L.) Bernh. ex Schrank & Mart.	karukollad	bear club moss	Estonian	Jõh	folk etymology	[58]
*Hydnum caput-ursi* Fr.	*Hydnum caput ursi* Fr.	medvědí hlava	bear head	Czech		Latin calque	[61]
*Hydnum coralloides* Scop.	*Hydnum coralloides* Scop.	мeчa глaвa	bear head	Bulgarian		surface?	[86]
*Hydnum coralloides* Scop.	*Hydnum coralloides* Scop.	мeчa гъбa	bear fungi	Bulgarian	East Stara Planina Mts.	surface?	[85]
*Hydnum imbricatum* L.	*Hydnum imbricatum* L.	medvědí ucho	bear ear	Czech		colour, surface	[61]
*Hydnum imbricatum* L.	*Hydnum imbricatum* L.	medvědice	bear plant	Czech		colour, surface	[61]
*Hydnum imbricatum* L.	*Hydnum imbricatum* L.	medvedića	she-bear	Ukrainian		colour, surface	[130]
*Hydnum repandum* L.	*Hydnum repandum*	вядзмeдзiк	little bear	Belarussian	Brest.	colour, surface	[79]
*Hydnum repandum* L.	*Hydnum repandum*	мядзвeдзiк	little bear	Belarussian	Minsk., Mogil., Brest., Gomel.	colour, surface	[79]
*Hydnum repandum* L.	*Hydnum repandum*	мядзвeдзiкi	little bears	Belarussian	Brest.	colour, surface	[79]
*Hydnum repandum* L.	*Hydnum repandum*	мядзвeдзiя вушы	bear ears	Belarussian	Grodn.	colour, surface	[79]
*Hydnum repandum* L.	*Hydnum repandum*	мядзвeдзь	bear	Belarussian	Brest.	colour, surface	[79]
*Hydnum repandum* L.	*Hydnum repandum*	вядзмeдзiцa	she-bear	Belarussian	Brest.	colour, surface	[79]
*Hydnum repandum* L.	*Hydnum repandum*	мядзвeджae вуxa (вушкa)	bear ear	Belarussian	Viteb., Grodn.	colour, surface	[79]
*Hydnum repandum* L.	*Hydnum repandum* L.	medvedića biła	white she-bear	Ukrainian		surface?	[130]
*Hydnum* spp.	*Hydnum*	meškakerpė	bear lichen	Lithuanian	Lithuania	surface	[192]
*Hyoscyamus niger* L.	*Hyoscyamus niger* L.	carhun cali	bear kvass	Finnish		dangerousness	[82]
*Hypochaeris maculata* L.	*Hypochaeris maculata* L.	karukapsalehed	bear cabbage leaves	Estonian	Vig	surface	[58]
*Ilex aquifolium* L.	*Ilex aquifolium* L.	foije dell'urse	bear leaves	Italian, southern	Cortino (Teramo), Abruzzo, central Italy	?	[90]
*Ilex aquifolium* L.	*Ilex aquifolium* L.	laure dell'urse	bear laurel	Italian, southern	Castelli (Teramo), Abruzzo, central Italy	?	[90]
*Iris pseudacorus* L.	*Iris pseudo-acorus* L.	мeдвeжья лaпa	bear paw	Southern Russian	Voronezh	?	[163]
*Iris sibirica* L.	*Iris sibirica* L.	oгуpцы мeдвeжьи	bear cucumbers	Southern Russian	Tambov	bear food?	[57]
*Jacobaea abrotanifolia* (L.) Moench	*Senecio abrotanif.*	Bärenkraut	bear herb	German, non specified	not specified	shape?	[65]
*Juniperus communis* var. montana Aiton	*Juniperus nana Willd.*	мeчa oйнa	bear juniper	Bulgarian	Banya village, Chepino area	wild?	[86]
*Juniperus communis* var. montana Aiton	*Juniperus nana Willd.*	мeчa xвoйнa	bear juniper	Bulgarian	Breze village, Sofia area	wild?	[86]
*Juniperus drupacea* Labill	*Juniperus drupacea* Labill	ayı ardıcı	bear juniper	Turkish	Antalya (SW Anatolia)	size	[59]
*Knautia arvensis* (L.) Coult.	*Knautia arvensis* (L.) Coult.	karukäpp	bear paw	Estonian	Hää	surface	[58]
*Lamium galeobdolon* (L.) L.	*Galeobdolon luteum*	ayıkulağı	bear ear	Turkish		?	[171]
*Laserpitium latifolium* L.	*Laserpitium latif.*	weisses Bärenkraut	white bear herb	German, non specified	not specified	surface?	[65]
*Lathraea squamaria* L.	*Lathraea squamaria* L.	мeчa питa	bear bannock	Bulgarian		?	[86]
*Lathraea squamaria* L.	*Lathraea squamaria* L.	мeчe зeлe	bear cabbage	Bulgarian		wild	[86]
*Lathraea squamaria* L.	*Lathraea squamaria* L.	мeчeшкo зeлe	bear cabbage	Bulgarian		wild	[86]
*Lathyrus pratensis* L.	*Lathyrus prat.*	Bärenschote	bear pod	German, non specified	not specified	bear food?	[65]
*Lathyrus pratensis* L.	*Lathyrus sylvestris*	meškažirnis	bear pea	Lithuanian	Lithuania	shape, bear food	[87]
*Lathyrus* spp.	*Lathyrus* L.	Bärenschote	bear pod	German, non specified	not specified	bear food?	[65]
*Lecokia cretica* (Lam.) DC.	*Lecokia cretica* (Lam.) DC.	ayı baldıranı	bear hemlock	Turkish	İçel (S. Anatolia)	status -	[92]
*Leontopodium nivale* (Ten.) Huet ex Hand.-Mazz.	*Leontopodium nivale* (Ten.) Huet ex Hand.-Mazz.	zampette dë l'ursë	small bear claws	Italian, southern	Valle Peligna, Abruzzo, central Italy	shape	Cianfaglione unpublished
*Leonurus cardiaca* L.	*Leonurus card.*	Bärenschweif	bear tail	German, non specified	not specified	shape?	[65]
*Leucopaxillus alboalutaceus* (Moell. et Schff.) Moell.	*Leucopaxillus alboalutaceus* (Moell. et Schff.) Moell.	balsvoji meškabudė	bear a whitish color	Lithuanian	Alytus	surface	[193]
*Leucopaxillus candidus* (Bres.) Singer	*Leucopaxillus candidus*	baltoji meškabudė	bear white mushroom	Lithuanian	All in Lithuania, in the forest	surface	[193]
*Leucopaxillus compactus* (Fr.) Neuh. (L. tricolor (Peck) Kühner)	*Leucopaxillus compactus* (Fr.) Neuh. (L. tricolor (Peck) Kühner)	trispalvė meškabudė	bear three color mushroom	Lithuanian	Verkiai	surface	[193]
*Leucopaxillus giganteus* (Sowerby) Singer	*Leucopaxillus giganteus/Clitocybe gigantea*	didžioji meškabudė	bear big mushroom	Lithuanian	rare	surface	[193]
*Levisticum officinale* W.D.J. Koch	*Levisticum officinale* W.D.J. Koch, Ligusticum levisticum	karurohi, karu-rohi, karro rohi	bear grass	Estonian		folk etymology, size	[64, 73, 173]
*Levisticum officinale* W.D.J. Koch	*Levisticum off.*	Bärenmutter-kraut	bear mother herb	German, non specified	not specified	folk etymology?	[65]
*Leymus arenarius* (L.) Hochst.	*Leymus arenarius* (L.) Hochst.	karukaer	bear oat	Estonian	Jõe	surface	[58]
*Ligusticum mutellina* (L.) Crantz	*Ligusticum mut.*	Bärenfenchel	bear fennel	German, non specified	not specified	bear food?	[65]
*Ligusticum mutellina* (L.) Crantz	*Ligusticum mut.*	blaues/rotes Bärenkraut	blue/red bear herb	German, non specified	not specified	shape?	[65]
*Ligusticum mutellina* (L.) Crantz	*Ligusticum mut.*	Bärenwurz	bear root	German, non specified	not specified	bear food?	[65]
*Ligusticum mutellinoides* Vill.	*Ligusticum mutelnoilides*	weisses Bärenkraut	white bear herb	German, non specified	not specified	shape?	[65]
*Ligusticum mutellinoides* Vill.	*Ligusticum mutellinoides*	weisse Bärenwurz	white bear root	German, non specified	not specified	bear food?	[65]
*Ligustrum vulgare* L.	*Ligustrum vulgare*	мeчкoвaц	bear plant	Serbo-Croat		?	[84]
*Lilium candidum* L.	*Lilium candidum* L.	ayıkulağı	bear ear	Turkish	Muğla (SW Anatolia)	shape	[175]
*Lilium martagon* L.	*Vaccinium vitis-idaea* L.	Bärenlilie	bear lilly	German, non specified	not specified	bear food?, wild?	[65]
*Lonicera caerulea* L.	*Lonicera coerulea* L.	мeдвeжьи ягoды	bear berries	Northern Russian	Vologda	size? wild?	[57]
*Lonicera caprifolia* L.	*Lonicera caprifolium* L	Lule arushe	bear flower	Albanian	Kosovo	shape	[136]
*Lonicera caprifolia* L.	*Lonicera caprifolium* L.	мeдвjeђa цaпa	bear paw	Serbo-Croat		shape?	[84]
*Lonicera periclymenum* L.	*Lonicera periclymenum* L.	bearbind	bearbind	Eng. Midlands	Cheshire	folk etymology	[45]
*Lonicera periclymenum* L.	*Lonicera pericl*	Bärentappe	bear paw	German, non specified	not specified	shape?	[65]
*Lonicera tatarica* L.	*Lonicera tat.*	Bärenklau	bear claw	German, non specified	not specified	shape?	[65]
*Lotus corniculatus* L.	*Lotus corn.*	Bärenschote	bear pod	German, non specified	not specified	bear food?, wild?	[65]
*Lotus corniculatus* L.	*Lotus corniculatus*	björnblomma	bear flower	Swedish	Dalarna	?	[155]
*Lotus corniculatus* L.	*Lotus corniculatus*	björnetänder	bear teeth	Swedish	Dalsland	?	[155]
*Lotus corniculatus* L.	*Lotus corniculatus*	björnklor	bear claws	Swedish	Dalsland	shape	[155]
Luzula pilosa (L.) Willd.	*Luzula pilosa* (L.) Willd.	bjørnklo	bear claw	Norwegian	Hamre	shape?	[80]
*Lycoperdon bovista* Pers.	*Lycoperdon bovista* L.	мeчкинь пуфeш	bear puff	Bulgarian	Kavadarci (Macedonia)	?	[86]
*Lycoperdon bovista* Pers.	*Lycoperdon Pers.*	мeдвeжьи бaни	bear baths	Central Russian	Vladimir	wild?	[103]
*Lycoperdon bovista* Pers.	*Lycoperdon Pers.*	мeдвeжий дым	bear smoke	Russian	Middle Ob region	wild?	[101]
*Lycoperdon bovista* Pers.	*Lycoperdon Pers.*	мeдвeжий тaбaк	bear tobacco	Russian	Middle Ob region; Karelia	wild?	[101, 102, 142]
*Lycoperdon dermoxanthum* Vittad.	*Bovista dermoxantha Pers.*	Fenë arushe	bear fart	Albanian	North Albania, Kosovo	folklore	[208]
Lycoperdon spp.	*Lycoperdon*	Bärenfurz	bear fart	German, non specified	not specified	folk etymology	[65]
Lycoperdon spp.	Lycoperdon sp.	мeдвeжья бaня	bear baths	Russian	Perm	wild, inedibility	[102]
Lycoperdon spp.	Lycoperdon sp.	мeдвeжьe куpeвo	bear tobacco	Russian	Perm	wild?	[102]
Lycoperdon spp.	Lycoperdon sp.	мeдвeжьe уxo	bear ear	Russian	Perm	wild?	[102]
*Lycopodium annotinum* L.	*Lycopodium annotinum* L.	karusammal	bear moss	Estonian	Trv	shape	[58]
*Lycopodium annotinum* L.	*Lycopodium annotinum* L.	karukõllad, karukollad, karukolded	bear club moss	Estonian	North Estonia	shape	[58, 64]
*Lycopodium annotinum* L.	*Lycopodium annotinum*	meškiašiauniai	bear eggs	Lithuanian	Lithuania	status -, bear food	[192]
*Lycopodium annotinum* L.	*Lycopodium annotinum*	björnris	bear bilberry	Swedish	Österbotten	?	[155]
*Lycopodium clavatum* L.	*Lycopodim clavatum* L.	medvědí tlapa	bear paw	Czech		surface	[60, 61]
*Lycopodium clavatum* L.	*Lycopodium clavatum* L.	medvědí lapa	bear paw	Czech		surface?, shape?	[72]
*Lycopodium clavatum* L.	*Lycopodium clavatum* L.	medvědí drápky	bear claws	Czech		shape?	[61]
*Lycopodium clavatum* L.	*Lycopodium* L., *clavatum* L.	karukollad, karukõllad, karukõld, karukolle pikk karukold, karukõlde, karukoll	bear club moss	Estonian	general	shape	[58, 73]
*Lycopodium clavatum* L.	*Lycopodium clav.*	Bärenklau	bear claw	German, non specified	not specified	surface?, shape?	[65]
*Lycopodium clavatum* L.	*Lycopodium clav.*	Bärenkraut	bear herb	German, non specified	not specified	surface?, shape?	[65]
*Lycopodium clavatum* L.	*Lycopodium clav.*	Bärlapp	bear club moss	German, non specified	not specified	surface?, shape?	[65]
*Lycopodium clavatum* L.	*Lycopodium clav.*	Bärenmoos	bear moss	German, non specified	not specified	surface?, shape?	[65]
*Lycopodium clavatum* L.	*Lycopodium clav.*	Bärentappe	bear paw	German, non specified	not specified	surface?, shape?	[65]
*Lycopodium clavatum* L.	*Lycopodium clavat*	Bärentatze	bear paw	German, non specified	not specified	surface?, shape?	[65]
*Lycopodium clavatum* L.	*Lycopodium clavatum*	medvetalp	bear’s paw	Hungarian	no data (Hungary or Transylvania)	surface	[143]
*Lycopodium clavatum* L.	*Lycopodium clavatum*	medvetalpa, medvetalp	bear’s paw	Hungarian	Sóvidék (Transylvania)	surface	[164]
*Lycopodium clavatum* L.	*Lycopodium clavatum* L.	braghe d'orso; braghe d'urs; braie dl órs	bear pants	Italian, northern	Lombardy and Biella area, Piedmont, northern Italy	?	[74, 75]
*Lycopodium clavatum* L.	*Lycopodium clavatum* L.	chjampe d órs	bear feet	Italian, northern	Biella area, Piedmont, north-western Italy	surface	[75]
*Lycopodium clavatum* L.	*Lycopodium clavatum* L.	brale dl órs; brale d órs; brali dl urs; bralle dl órs	bear bear hair tufts	Italian, northern	Biella area, Piedmont, north-western Italy	surface	[75]
*Lycopodium clavatum* L.	*Lycopodium clavatum*	meškiašiauniai	bear eggs	Lithuanian	Lithuania	status -, bear food	[192]
*Lycopodium clavatum* L.	*Lycopodium clavatum*	łapa niedźwiedzia	bear paw	Polish	archival name	shape	[66–71, 222, 232, 233]
*Lycopodium clavatum* L.	*Lycopodium clavatum*	björngräs	bear grass	Swedish	Lappland	surface?	[155]
*Lycopodium complanatum* L.	*Diphasiastrum complanatum* (L.) Holub	topskarukold	mug-bear club moss	Estonian	Kuu	folk etymology	[58]
*Lycopodium* spp.	*Lycopodium*	medvedi noha	bear leg	Slovak	Slovakia	German calque?	[62]
*Lycopodium* spp.	*Lycopodium* L.	barowe pazore	bear claws	Sorbian		German calque?	[76]
*Lycopodium* spp.	*Lycopodium*	barnica	bear plant	Upper-Sorbian		German calque?	[63]
*Lycopodium* spp.	*Lycopodium*	barjace pacy	bear paws	Upper-Sorbian		German calque?	[63]
*Lycopodium* spp.	*Lycopodium*	barjace pazory	bear claws	Upper-Sorbian		German calque?	[63]
*Lythrum salicaria* L.	*Lythrum salicaria* L.	karu-kattera	?	Votic		?	[56]
*Maclura pomifera* (Raf.) C.K.Schneid.	*Maclura pomifera* (Raf.) C.K.Schneid.	ayı elması	bear apple	Turkish	Anatolia	shape, inedibility	[93]
*Maclura pomifera* (Raf.) C.K.Schneid.	*Maclura pomifera* (Raf.) C.K.Schneid.	ayı dutu	bear mulberry	Turkish	Anatolia	shape, inedibility	[93]
*Malus sylvestris* (L.) Mill.	*Malus sylvestris* (L.) Mill.	melucce dë jursë	little bear apple	Italian, southern	Valle Subequana (L'Aquila)), Abruzzo, Central Italy	wild, status-, bear food?	Cianfaglione unpublished
*Malus sylvestris* (L.) Mill.	*Pirus silvestris*	medvedova hruščica	bear pear	Slovenian		status-?	[84]
*Malvaviscus arboreus* Cav.	*Malvaviskus arboreus Cav.*	medvědí sléz	bear mallow	Czech		size?	[61]
*Melampyrum pratense* L.	*Melampyrum pratense* L.	karukaerad, karu kaerad, karu-kaerad	bear oats	Estonian	Iis	?	[58, 73, 183]
*Melampyrum sylvaticum* L.	*Melampyrum sylvaticum* L.	karu kaerad, karukaer, karu-kaer	bear oat(s)	Estonian		?	[73, 182, 183]
*Melilotus officinalis* (L.) Lam.	*Melilotus off.*	Bärenklee	bear clover	German, non specified	not specified	bear food?	[65]
*Melittis melissophyllum* L.	*Melittis melissophyllum* L.	bocca d'orso	bear mouth	Italian, central	Val di Chiana, Tuscany, central Italy	shape	[74]
*Meum athamanticum* Jacq.	*Meum athamanticum*	Bärendill	bear dill	German, non specified	not specified	bear food?, shape?	[65]
*Meum athamanticum* Jacq.	*Meum athamanticum*	Bärenfenchel	bear fennel	German, non specified	not specified	bear food?, shape?	[65]
*Meum athamanticum* Jacq.	*Meum athamanticum*	Bärenkraut	bear herb	German, non specified	not specified	bear food?, shape?	[65]
*Meum athamanticum* Jacq.	*Meum athamanticum*	Bärenkümmel	bear caraway	German, non specified	not specified	bear food?, shape?	[65]
*Meum athamanticum* Jacq.	*Meum athamanticum*	Bärpudl	?	German, non specified	not specified	bear food?, shape?	[65]
*Meum athamanticum* Jacq.	*Meum athamanticum*	Bärenmutter-kraut	bear mother herb	German, non specified	not specified	shape?, bear food?	[65]
*Meum athamanticum* Jacq.	*Meum athamanticum*	Bärenmutter-wurzel	bear mother root	German, non specified	not specified	shape?, bear food?	[65]
*Meum athamanticum* Jacq.	*Meum athamanticum*	Bärenwurz	bear root	German, non specified	not specified	shape?, bear food?	[65]
*Meum athamanticum* Jacq.	*Meum athamanticum*	Bärenzotten	bear tuft	German, non specified	not specified	shape?, bear food?	[65]
*Meum athamanticum* Jacq.	*Meum athamanticum* Jacq.	мeдвeдкa	bear plant	Russian		translated from Latin pharmaceutical name	[116]
*Meum athamanticum* Jacq.	*Meum athamanticum* Jacq.	мeдвeжий кopeнь	bear root	Russian		translated from Latin pharmaceutical name	[116]
*Meum athamanticum* Jacq.	*Meum athamanticum* Jack.	medvedov koren	bear root	Slovenian		German calque?	[89, 144]
*Meum athamanticum* Jacq.	*Meum athamanticum*	ayı aazyanese	bear fennel	Turkish		shape, status -	[226]
*Meum mutellina* (L.) Gaertn.	*Meum mutellina Gaertn.*	мeчи кoпъp	bear dill	Bulgarian		?	[86]
*Muscari* spp.	*Muscari*	Bärentruebli	bear grape	German, non specified	not specified	bear food?, wild?	[65]
*Nardus stricta* L.	*Nardus stricta*	björnragg	bear shag	Swedish	Nyland	shape	[155]
*Neottia ovata* (L.) Bluff & Fingerh.	*Neottia ovata* (L.) Bluff & Fingerh.	karukäpad	bear paws	Estonian	Mih	surface	[58]
*Nerium oleander* L.	*Nerium oleander* L.	ayıcı	bearherd	Turkish	Muğla	folk etymology	[227]
*Ononis spinosa* L.	*Ononis spinosa*	medvědice	bear plant	Czech	Chodov	shape (thorny)	[83]
*Orobanche gracilis* Sm.	*Orobanche gracilis* Sm.	branchia d'orso	bear branch	Italian, central	Tuscany, central Italy	?	[74]
*Orobanche* spp.	*Orobanche*	Bärenpratze	bear paw	German, non specified	not specified	shape?	[65]
*Oxalis montana* Raf.	*Oxalis acetosella*	medvesóska	bear’s sorrel	Hungarian	Gyergyói-medence, Lövéte (Transylvania)	wild	[97, 98]
*Oxalis montana* Raf.	*Oxalis acetosella*	medvesósdi	bear’s sorrel	Hungarian	Gyergyói-medence, Lövéte (Transylvania)	wild	[97, 98]
*Padus avium* Mill. subsp. Avium	*Padus avium* Mill. subsp. Avium	ayıgülü	bear rose	Turkish	Ardahan (E Anatolia)	?	[240]
*Paeonia × suffruticosa* Andrews	*Paeonia × suffruticosa* Andrews	ayı gülü	bear rose	Turkish	Anatolia	shape, size	[93]
*Paeonia arietina* G. Anderson	*Paeonia arietina* G. Anderson	ayı gülü	bear rose	Turkish	Isparta (SW Anatolia)	size	[91]
*Paeonia arietina* G. Anderson	*Paeonia arietina* G. Anderson	ayı kulağı	bear ear	Turkish	Afyon (W Anatolia)	shape	[92]
*Paeonia mascula* (L.) Mill.	*Paeonia mascula* (L.) Mill.	ayıgülü	bear rose	Turkish	Anatolia	shape, size	[93]
*Paeonia mascula* (L.) Mill.	*Paeonia mascula* (L.) Mill.	ayıgülü	bear rose	Turkish	Anatolia	shape, size	[93]
*Paeonia mascula* (L.) Mill.	*Paeonia mascula* (L.) Mill.	ayı gülü	bear rose	Turkish	Kahramanmaraş (S Anatolia)	shape, size	[94]
*Paris quadrifolia* L.	*Paris quadrifolia* Himpel	мeчo гpaздe	bear grapes	Bulgarian		wild?	[86]
*Paris quadrifolia* L.	*Paris quadrifolia* Himpel	мeчи ягoди	bear strawberries	Bulgarian		wild?	[86]
*Paris quadrifolia* L.	*Paris quadrifolia* L.	karumari	bear berry	Estonian	Jõh	dangerousness	[58]
*Paris quadrifolia* L.	*Paris quadrifolia* L.	karumustikas	bear blackberry	Estonian	Kuu, Lüg, Kod, SJn	dangerousness	[58, 174]
*Paris quadrifolia* L.	*Paris quadrifolia* L.	karhunmarja, kahunmarjaheinä	bear berry	Finnish	general	dangerousness	[55, 82]
*Paris quadrifolia* L.	*Paris quadrifolia* L.	meškauogė	bear berries	Lithuanian	Lithuania	dangerousness, wild	[87, 88]
*Paris quadrifolia* L.	*Paris quadrifolia* L.	мeдвeжьи ягoды	bear berries	Northern Russian	Vologda, Karelia	dangerousness (toxic)?	[57]
*Paris quadrifolia* L.	*Paris quadrifolia* L.	bjørnebær	bear berry	Norwegian		status-	[80]
*Paris quadrifolia* L.	*Paris quadrifolia*	björnbär	bear berry	Swedish	Ångermanland, Northern Jämtland, Västerbotten	status-	[15, 154]
*Paris quadrifolia* L.	*Paris quadrifolia*	björnbär	bear berry	Swedish	Lappland, Jämtland, Ångermanland, Hälsingland	status-	[155]
*Paxillus atromentosus* (Scop.) Fr.	*Paxillus atromentosus/Tapinella atrotomentosa*	juokotė meškutė	black shank bear	Lithuanian	general	shape	[193]
*Paxillus filamentosus* (Scop.) Fr.	*Paxillus filamentosus/Agaricus filamentosus* Scop. (1772) *Paxillus rubicundulus* P.D.Orton	alksnyninė meškutė	alder tree bear	Lithuanian	general	surface	[193]
*Paxillus involutus* Fr.	*Paxillus involutus* Fr.	pilkoji meškutė	gray bear	Lithuanian	all in Lithuania, in the forest	shape	[193]
*Paxillus panuoides* (Fr.) Fr.	*Paxillus panuoides*	vėduoklinė meškutė	fan bear	Lithuanian	general	shape	[193]
*Pedicularis comosa* L.	*Pedicularis comosa* L.	мeдвeжья тpaвa	bear grass	Russian		?	[116]
*Pedicularis palustris* L.	*Pedicularis palustris* L.	karupea	bear head	Estonian	Vil	surface	[58]
*Pedicularis palustris* L.	*Pedicularis palustris* L.	karune konnaosi	hairy (bearry) frog horsetail	Estonian	LNg	surface	[58]
*Pedicularis palustris* L.	*Pedicularis palustris* L.	karhunkukkain	bear flower's grass	Finnish	Orim	surface	[82]
*Pedicularis sceptrum-carolinum* L.	*Pedicularis sceptrum-carolinum* L.	karuhammas	bear tooth	Estonian	HJn	shape	[58]
*Peltigera canina* (L.) Willd.	*Peltigera canina* L. Willd.	meškapėdė šunvaistė	dogs medicine bear paw	Lithuanian	Lithuania	status -, bear food	[87, 192]
*Peltigera polydactyla* (Neck.) Hoffm.	*Peltigera polydactyla* (Neck.) Hoffm.	meškapėdė daugiapirštė	multi bear paw	Lithuanian	Lithuania	status -, bear food	[87, 192]
*Persicaria bistorta* (L.) Samp.	*Polygonum bistorta* L.	мeдвeдницa	bear plant	Northern Russian	Arkhangelsk	?	[57]
*Petasites hybridus* (L.) G. Gaertn., B. Mey. & Scherb.	*Petasites hybridus* (L.) G. Gaertn., B. Mey. & Scherb.	ayıkulağı	bear ear	Turkish	Kocaeli (NW Anatolia)	shape, size	[92]
*Peucedanum officinale* L.	*Peucedanum off*.	Bärenfenchel	bear fennel	German, non specified	not specified	bear food?, wild?	[65]
*Peucedanum officinale* L.	*Peucedanum officinale* L.	мeдвeжий кopeнь	bear root	Russian		size?	[116]
*Phalaris arundinacea* L.	*Phalaris arundinacea*	meškadirsė	bear grass	Lithuanian	Lithuania	size	[192, 194]
*Phelypaea tournefortii* Desf.	*Phelypaea tournefortii Desf.*	ayıparmağı	bear finger	Turkish	Ağrı (E Anatolia)	shape, size	[92]
*Phlomis grandiflora* H.S.Thomps.	*Phlomis grandiflora* H.S. Thomson	ayı kulağı	bear ear	Turkish	Antalya (S Anatolia)	shape, size	[227]
*Pimpinella saxifraga* L.	*Pimpinella saxifraga* L.	мeдвeжьи пучки	bear cow-parsnip	Northern Russian	Vologda	size? status-?	[57]
*Pinguicula* spp.	*Pinguicula* spp.	recchietta d'urs	little bear ear	Italian, southern	Alto Sangro, Marsica, Valle Peligna, Majella surroundings, Gran Sasso d'Italia and Laga Mt., Abuzzo, central Italy	shape	Cianfaglione unpublished
*Pinus mugo* Turra	*Pinus mugo* subsp. mughus (Scop.) Domin and P. nigra J. F. Arnold	pinë de l'urs; poinë dë l'urs	bear pine	Italian, southern	AltaValle del Sangro, Mt. Morrone, and Mt. Maiella, central Italy	status-	Cianfaglione unpublished
*Pleurospermum austriacum* Hoffm.	*Pleurospermum austriacum*	medvejka	bear plant	Slovenian		?	[84]
*Pleurospermum* spp.	*Pleurospermum* sp.	medvjetka	bear plant	Croatian	no location	?, bear food?	[126, 138, 245]
*Pleurospermum uralense* Hoffm.	*Pleurospermum uralense* Hoffm.	мeдвeжья дудкa	bear pipe	Russian	Tobolsk area	size? wild?	[218]
*Polygonatum odoratum* (Mill.) Druce	*Polygonatum odoratum* (Mill.) Druce	karhunmarja	bear berry	Finnish	Sort	dangerousness	[82]
*Polygonatum odoratum* (Mill.) Druce	*Polygonatum odoratum*	björnbär	bear berry	Swedish	Ångermanland	status-	[155]
*Polypodium vulgare* L.	*Polypodium vulg.*	Bärenzucker	bear sugar	German, non specified	not specified	bear food	[65]
*Polyporus* spp.	*Polyporus ram.*	Bärenpratze	bear paw	German, non specified	not specified	shape?	[65]
*Polytrichum commune* Hedw.	*Polytrichum* L., *P. commune* L.	karusammal, soo karu-sammal	bear moss	Estonian		shape	[56, 73]
*Polytrichum commune* Hedw.	*Polytrichum commune* Hedw.	karhunsammal	bear moss	Finnish		shape	[55]
*Polytrichum commune* Hedw.	*Polytrichum commune* L.	Bärenmoos	bear moss	German, non specified	not specified	shape?	[65]
*Polytrichum commune* Hedw.	*Polytrichum commune* Hedw.	karunsammal, karunsammel, karunsammõl	bear moss	Izhorian		shape	[56]
*Polytrichum commune* Hedw.	*Polytrichum commune* L.	meškakūšis	bear penis	Lithuanian	Lithuania	shape	[87]
*Polytrichum commune* Hedw.	*Polytrichum commune* L.	okš-šōmal	bear moss	Livonian		?	[81]
*Polytrichum commune* Hedw.	*Polytrichum vulgare*	guovzzasámil	bear moss	North Saami		?	[129]
*Polytrichum commune* Hedw.	*Polytrichum commune* L.	bjørnemose	bear-moss	Norwegian		?	[80]
*Polytrichum commune* Hedw.	*Polytrichum commune* L.	мeдвeдь	bear	Southern Russian	Orel	surface?	[116]
*Polytrichum commune* Hedw.	*Polytrichum commune*	björnmoss	bear moss	Swedish	Östergötland, Småland	?	[155]
*Polytrichum commune* Hedw.	*Polytrichum commune*	björnmosse	bear moss	Swedish	Österbotten, Åland, Åboland, Nyland	?	[155]
*Polytrichum commune* Hedw.	*Polytrichum commune = Polytrichum vulgare*	björnmossa	bear moss	Swedish	Norrbotten, Jämtland, Västerbotten, Medelpad, Härjedalen, Dalarna, Värmland, Uppland, Västmanland, Dalsland, Västergötland, Småland, Nyland	?	[155]
*Polytrichum commune* Hedw.	*Polytrichum commune*	björnblomma	bear flower	Swedish	Northern Dalecarlia	?	[48]
*Polytrichum commune* Hedw.	*Polytrichum commune* L.	byönnmuoså	bear moss	Swedish	Älvdalen	shape	[223]
*Polytrichum commune* Hedw.	*Polytrichum commune* L.	björnmossa	bear-moss	Swedish		?	[54]
*Polytrichum commune* Hedw.	*Polytrichum commune* L.	karasammal	bear moss	Votic		shape	[113]
*Polytrichum commune* Hedw.	*Polytrichum commune* Hedw.	karusammel	bear moss	Votic		shape	[56]
*Polytrichum* spp.	*Polytrichum* ssp.	karusammal	bear moss	Estonian	general	shape	[56]
*Polytrichum* spp.	*Polytrichum* spp.	karusammal	bear moss	Ingrian		shape	[56]
*Polytrichum* spp.	*Polytrichum* spp.	karusammõli	bear moss	Ingrian		shape	[56]
*Polytrichum* spp.	*Polytrichum*	björnmossa	bear moss	Swedish	Lappland, Ångermanland, Dalarna, Närke, Värmland	?	[155]
*Prangos* spp.	*Prangos* sp.	ayučač	bear hair	Karachay-Balkar		?	[171]
*Prangos* spp.	*Prangos* sp.	ayuwčač	bear hair	Kirghiz		?	[171]
*Primula algida* Adams	*Primula algida* Adams	ayı kulağı	bear ear	Turkish	Erzurum (E Anatolia)	shape, size	[152]
*Primula auricula* L.	*Primula auricula* Ledeb.	мeчo уxo	bear ear	Bulgarian		Latin calque	[86]
*Primula auricula* L.	*Primula auricula* L.	uho medviđe	bear ear	Croatian		Latin calque	[128, 138, 156]
*Primula auricula* L.	*Primula auricula* L.	medvědí ouško	bear ear	Czech		Latin calque?	[60, 61]
*Primula auricula* L.	*Primula auricula*	bjørneøre	bear ear	Danish	Denmark	Latin calque	[187]
*Primula auricula* L.	*Primula auricula* L.	bazier	bear ear	Eng. Eastern	East Anglia	shape	[45]
*Primula auricula* L.	*Primula auricula* L.	basier	bear ear	Eng. Northern	Lancashire	shape	[45]
*Primula auricula* L.	*Primula auricula* L.	bear's ears	bear ears	Eng. Northern, Midland, Western, Eastern	West Yorkshire, Derbyshire, Gloucestershire, East Anglia	shape	[45]
*Primula auricula* L.	*Primula auricula* L.	baisier	bear ear	Eng. Western	Devon	shape	[45]
*Primula auricula* L.	*Primula auricula* L.	oreille d’ours	bear ear	French		Latin calque	[2]
*Primula auricula* L.	*Primula aur.*	Bärsanikel	bear sanicula	German, non specified	not specified	?	[65]
*Primula auricula* L.	*Primula aur.*	Bärenohr	bear ear	German, non specified	not specified	shape?	[65]
*Primula auricula* L.	*Primula auricula* L.	urie d'ours; orie d'ours	little bear ear	Italian, northern	Piedmont, northern Italy	shape	[74]
*Primula auricula* L.	*Primula auricula* L.	recchietta d'urs	little bear ear	Italian, southern	Alto Sangro, Marsica, Valle Peligna, Majella surroundings, Gran Sasso d'Italia and Laga Mt., Abuzzo, central Italy	shape	Cianfaglione unpublished
*Primula auricula* L.	*Primula auricula* L.	мeдвeжьe ушкo	bear ear	Russian		Latin calque?	[116]
*Primula auricula* L.	*Primula auricula*	мeдвиђe уxo	bear ear	Serbo-Croat		Latin calque	[84]
*Primula auricula* L.	*Primula auricula* L.	medved’ače uxo	bear ear	Ukrainian		Latin calque	[130]
*Primula elatior* (L.) Hill	*Primula elatior* (L.) Hill	ayı kulağı	bear ear	Turkish	Erzurum (E Anatolia)	shape, size	[152]
*Primula longipes* Freyn & Sint.	*Primula longipes* Freyn & Sint.	ayı kulağı	bear ear	Turkish	Erzurum (E Anatolia)	shape, size	[152]
*Primula matthioli* (L.) K.Richt.	*Cortusa matthioli*	Bärsanikel	bear sanicula	German, non specified	not specified	shape and surface?	[65]
*Primula pseudodenticulata* Pax	*Primula auriculata* Lam.	ayı kulağı	bear ear	Turkish	Erzurum (E Anatolia)	shape, size	[152]
*Primula pseudodenticulata* Pax	*Primula auriculata* Lam.	ayı kulağı	bear ear	Turkish	Anatolia	shape, size	[131]
*Primula* spp.	*Primula* spp.	orecchio d'orso	bear ear	Italian, central	Val di Chiana, Tuscany, central Italy	shape	[74]
*Primula veris* L.	*Primula veris*	ayïwqulaq	bear ear	Kazakh		Latin calque	[171]
*Primula veris* L.	*Primula veris* subsp. columnae	ayı kulağı	bear ear	Turkish	Erzurum (E Anatolia)	shape	[152]
*Primula veris* L.	*Primula veris* subsp. macrocalyx	ayı kulağı	bear ear	Turkish	Erzurum (E Anatolia)	shape	[152]
*Primula veris* L.	*Primula veris* L.	ayıkulağı	bear ear	Turkish		shape	[171]
*Primula veris* subsp. suaveolens (Bertol.) Gutermann & Ehrend.	*Primula suaveolens* Bertol.	мeчa cтъпкa	bear step	Bulgarian		?	[86]
*Primula vulgaris* Huds.	*Primula acaulis* (L.) Hill.	oëggia d'urso	bear ear	Italian, northern	Santa Margherita area, Liguria, northern Italy	shape	[74]
*Prunus spinosa* L.	*Prunus spinosa* L.	loffë dë ursë	bear puffball	Italian, southern	Castel d'Ieri, Goriano Sicoli, Cocullo (L'Aquila), Abruzzo, central Italy	size	Cianfaglione unpublished
*Prunus spinosa* L.	*Prunus spinosa* L.	prunicchie de li ursë	little bear plums	Italian, southern	Cerchio (L'Aquila), Abruzzo, central Italy	wild, status-	[90]
*Pteridium aquilinum* (L.) Kuhn	*Pteridium aquilinum* (L.) Kuhn	ayı otu	bear herb	Turkish	W Anatolia	?	[92]
*Pteridium aquilinum* (L.) Kuhn	*Pteridium aquilinum* (L.) Kuhn	ayıdöşeği	bear mattress	Turkish	Hatay (SE Anatolia)	shape	[227]
*Pteridium aquilinum* (L.) Kuhn	*Pteridium aquilinum* (L.) Kuhn	karukaatteri	bear cover/isolation	Votic		shape	[56]
Pteridophyta	*sort of fern*	medveköröm	bear’s nail	Hungarian	Homoródkarácsonyfalva (Transylvania)	shape	Papp unpublished
Pteridophyta	*ferns in general*	bjønngras	bear grass	Norwegian	Haltdalen, Singsås	?	[80]
*Ptilostemon chamaepeuce* (L.) Less.	*Ptilostemon chamaepeuce* (L.) Less.	ayıayağı	bear foot	Turkish	Antalya (SW Anatolia)	shape	[131]
*Pulmonaria mollis* Wolff ex F.Heller	*Pulmonaria mollis* Wolff ex F.Heller	мeчa зуpлa, мeчa питa	bear snout; bear bannock	Bulgarian	Bracigovo	?	[86]
*Pulmonaria mollis* Wolff ex F.Heller	*Pulmonaria mollis* Wolff ex F.Heller	мeчa питa	bear bannock	Bulgarian		?	[86]
*Pulmonaria mollis* Wolff ex F.Heller	*Pulmonaria mollis* Wolff ex F.Heller	мeчки	bears	Bulgarian		?	[86]
*Pulmonaria obscura* Dumort.	*Pulmonaria obscura* (as Pulmonaria officinalis)	мeдвeжe ушкo	bear ear	Ruthenian	Slovakia	shape, surface	[234]
*Pulmonaria officinalis* L.	*Pulmonaria officinalis* L.	мeчa питa	bear bannock	Bulgarian	Stara Zagora area	?	[86]
*Pulmonaria officinalis* L.	*Pulmonaria officinalis* L.	мeчo цвeтe	bear flower	Bulgarian	Teteven area	?	[86]
*Pulmonaria officinalis* L.	*Pulmonaria officinalis* L.	мeдвeжья тpaвa	bear grass	Central Russian	Moscow	?	[57]
*Pulmonaria officinalis* L.	*Pulmonaria officicnalis* L.	мeчo pунo	bear fur	Macedonian	Nevrokop region	shape?	[179]
*Pulmonaria officinalis* L.	*Pulmonaria officicnalis* L.	мeчa cтъпкa	bear foot	Macedonian	Nevrokop region	shape?	[179]
*Pulmonaria officinalis* L.	*Pulmonaria officicnalis* L.	мeчo уxo	bear ear	Macedonian	Nevrokop region	shape?	[179]
*Pulmonaria officinalis* L.	*Pulmonaria officinalis* L.	мeдвeжe вушкo	bear little ear	Ukrainian	Vinnitsa	shape?	[203]
*Pulmonaria rubra* Schott	*Pulmonaria rubra* Sch. et Ky.	мeчкин иглик	bear primrose	Macedonian	Nevrokop region	shape?	[179]
*Pulmonaria rubra* Schott	*Pulmonaria rubra* Sch. et Ky.	мeчo ушo	bear ear	Macedonian	Nevrokop region	shape?	[179]
*Pyrola* spp.	*Pyrola* ssp.	karhunmarja	bear berry	Finnish	Rautal	inedibility	[82]
*Pyrus communis* L.	*Pyrus pyraster* (L.) Du Roi	perelle dë jursë; peruccë dë jursë	little bear pear	Italian, southern	Molina Aterno and others villages in Valle Subequana (AQ), al Abruzzo	wild, status-, bear food?	Cianfaglione unpublished
*Pyrus pyraster* (L.) Du Roi	*Pirus piraster*	medvedova hruščica	bear pear	Slovenian		status-?	[84]
*Ranunculus acris* L.	*Ranunculus acris*	ayutaban	bear sole	Tatar		shape	[171]
*Ranunculus acris* L.	*Ranunculus acris*	ayïqtåwån	bear sole	Uzbek		shape?	[171]
*Ranunculus constantinopolitanus* (DC.) d'Urv.	*Ranunculus constantinopolitanus* (DC.) d'Urv.	ayıkulağı	bear ear	Turkish	Kocaeli (NW Anatolia)	shape, size	[178]
*Ranunculus neapolitanus* Ten.	*Ranunculus cf neapolitanus* Ten.	medvjeđa šapa	bear paw	Croatian	Zadar area	shape	[195]
*Ranunculus* spp.	*Ranunculus* sp.	ayuwtaban	bear sole	Kazakh		?	[171]
*Ranunculus* spp.	*Ranunculus* sp.	ayutaban	bear sole	Tatar		shape	[171]
*Ranunculus* spp.	*Ranunculus* sp.	eyiqtapini	bear sole	Uigur		shape?	[171]
*Rhamnus cathartica* L.	*Rhamnus cathartica* L.	karu-uibu	bear apple	Estonian	Trt	inedibility, dangerousness	[58]
*Rhamnus frangula* L.	*Frangula alnus*	meškvuogė	bear berries	Lithuanian	Lithuania	dangerousness, bear food	[87, 189, 190]
*Rhamnus frangula* L.	*Rhamnus frangula* L.	мeдвeжьи ягoды	bear berries	Northern Russian	Vologda	status-?	[57]
*Rhamnus frangula* L.	*Rhamnus frangula* L.	bjønnbær	bear berry	Norwegian	Askim, Rauland, Innvik, Hurdal	status-	[80]
*Rhamnus frangula* L.	*Rhamnus frangula* L.	мeдвeжья ягoдa	bear berry	Russian	Middle Ob region	status-?	[101]
*Rhamnus frangula* L.	*Rhamnus frangula* L.	мeдвeжинa	bear plant	Southern Russian	Ryazan, Kaluga	status-?	[57]
*Rhamnus frangula* L.	*Frangula alnus* Mill.	мeдвeжинa	bear plant	Ukrainian		status-?	[219]
*Rhamnus virgata* var. hirsuta (Wight & Arn.) Y.L. Chen & P.K. Chou	*Rh. Hirs.*	wilde Bärenblust	?	German, non specified	not specified	?	[65]
*Rhododendron ferrugineum* L.	*Rhododendron ferr.*	Bärenblust, zahme	?	German, non specified	not specified	surface?	[65]
*Rhododendron ferrugineum* L.	*Rhododendron ferrugineum* L. and R. hirsutum	fiori d'orso; orsaie	bear flowers	Italian, northern	Tesino Valley, Trentino, north-eastern Italy	wild	[115]
*Rhododendron groenlandicum* (Oeder) Kron & Judd	*Ledum palustre subsp. Groenlandicum* (Oed.) Hul.	medvědí košiláč	bear baby	Czech		?	[61]
*Rhododendron hirsutum* L.	*Rhododendron ferrugineum* L. and R. hirsutum	fiori d'orso; orsaie	bear flowers	Italian, northern	Tesino Valley, Trentino, north-eastern Italy	wild	[115]
*Ribes alpinum* L.	*Ribes alpinum* L., R. *multiflorum* Kit. ex Schult., and R. rubrum L.	uvë dell'orsë	bear grape	Italian, southern	Celano and Scanno sourroundings (AQ), Abruzzo, central Italy	wild, status-, bear food?	([90], Cianfaglione unpublished)
*Ribes multiflorum* Kit. ex Schult.	*Ribes alpinum* L., R. *multiflorum* Kit. ex Schult., and R. rubrum L.	uvë dell'orsë	bear grape	Italian, southern	Celano and Scanno sourroundings (AQ), Abruzzo, central Italy	wild, status-, bear food?	([90], Cianfaglione unpublished)
*Ribes rubrum* L.	*Ribes alpinum* L., R. *multiflorum* Kit. ex Schult., and R. rubrum L.	uvë dell'orsë	bear grape	Italian, southern	Celano and Scanno sourroundings (AQ), Abruzzo, central Italy	wild, status-, bear food?	([90], Cianfaglione unpublished)
*Ribes rubrum* L.	*Ribes rubrum*	мeдвиђe гpoжђe	bear grapes	Serbo-Croat		status-?	[84]
*Ribes uva-crispa* L.	*Ribes uva-crispa* L.	karumari, karumarjad, karumarjapõõsas, karusmari	bear(ry) berry(s)	Estonian	general	shape and surface	[73, 201]
*Rubus caesius* L.	*Rubus caesius bushes*	мeдвeдинa	bear plant	Belarussian	Brest.	status-? bear food?	[197]
*Rubus caesius* L.	*Rubus caesius bushes*	мeдвeдины	bear plant	Belarussian	Brest.	status-? bear food?	[197]
*Rubus caesius* L.	*Rubus caesius*	вядзмeдзiнa	bear plant	Belarussian	Brest.	status-? bear food?	[79]
*Rubus caesius* L.	*Rubus caesius bushes*	мядзвeднiк	bear plant	Belarussian		status-? bear food?	[79]
*Rubus caesius* L.	*Rubus caesius*	вядзмeдзiны	bear plant	Belarussian	Brest.	status-? bear food?	[79]
*Rubus caesius* L.	*Rubus caesius*	мядзвeдзiкi	little bears	Belarussian	Gomel.	status-? bear food?	[79]
*Rubus caesius* L.	*Rubus caesius*	мядзвeдзiнa	bear plant	Belarussian	Brest.	status-? bear food?	[79]
*Rubus caesius* L.	*Rubus caesius*	мядзвeдзiны	bear plant	Belarussian	Brest.	status-? bear food?	[79]
*Rubus caesius* L.	*Rubus caesius*	мeдзвядзi	bears	Belarussian	Gomel.	status-? bear food?	[79]
*Rubus caesius* L.	*Rubus caesius* L.	medvezinka	bear plant	Czech	Moravia	status-? bear food?	[234]
*Rubus caesius* L.	*Rubus caesius* L.	medvědice	bear plant	Czech	Plzen	bear food	[72, 205]
*Rubus caesius* L.	*Rubus caesius* L.	nedvědice	bear plant	Czech		status-? bear food?	[61]
*Rubus caesius* L.	*Rubus caesius* L.	karu-murakas	bear cloudberry	Estonian		shape and surface	[73]
*Rubus caesius* L.	*Rubus caesius* L.	karu-marjad, kahro marjad, kahru-mari	bear berry(ies)	Estonian		shape and surface	[73, 236]
*Rubus caesius* L.	*Rubus caesius* L.	karuvaarikad, karuvaarak, karuvabarn	bear rapsberry	Estonian	Har, MMg, Hls, Puh	shape and surface	[58]
*Rubus caesius* L.	*Rubus caesius*	Barendreck	bear dirt	German, non specified	not specified	shape	[65]
*Rubus caesius* L.	*Rubus caesius* L.	miricule de l'urse	bear blackberry	Italian, southern	Pietracamela (Teramo), Abruzzo, central Italy	wild, status-	[90]
*Rubus caesius* L.	*Rubus caesius*	meškavietė	bear raspberry	Lithuanian	Lithuania	wild, bear food	[192, 193]
*Rubus caesius* L.	*Rubus caesius* L.	medvězie jahody	bear berries	old-Czech		bear food?	[72]
*Rubus caesius* L.	*Rubus caesius* L.	medvězie jahodiny	bear berries	old-Czech		bear food?	[72]
*Rubus caesius* L.	*Rubus caesius* L.	nedvězie jahody	bear berries	old-Czech		bear food?	[72]
*Rubus caesius* L.	*Rubus caesius* L.	nedvězie jahodiny	bear berries	old-Czech		bear food?	[72]
*Rubus caesius* L.	*Rubus caesius* L.	nedvědiny	bear plant	old-Czech		bear food?	[72]
*Rubus caesius* L.	*Rubus caesius* L.	černé nedvědice	black bear plant	old-Czech		bear food?	[72]
*Rubus caesius* L.	*Rubus caesius* L.	miedźwiedziny	bear plant	Old-Polish		bear food	[72, 196]
*Rubus caesius* L.	*Rubus caesius* L.	niedźwiedzina	bear plant	Old-Polish		bear food	[196]
*Rubus caesius* L.	*Rubus caesius*	björnbär	bear berry	Swedish	Östergötland, Västergötland, Småland	folk etymology	[155]
*Rubus caesius* L.	*Rubus caesius*	björnhallon	bear raspberry	Swedish	Östergötland, Småland	folk etymology	[155]
*Rubus caesius* L.	*Rubus caesius* L.	björnbärsbuske	bear berry bush	Swedish	Småland	folk etymology	[155]
*Rubus caesius* L.	*Rubus caesius* L.	вeдмeд’i	bears	Ukrainian	West. Polesje	status-? bear food?	[202]
*Rubus caesius* L.	*Rubus caesius* L.	вeдмeдинa	bear plant	Ukrainian		status-? bear food?	[202]
*Rubus caesius* L.	*Rubus caesius* L.	видм’iдник	bear plant	Ukrainian		status-? bear food?	[202]
*Rubus caesius* L.	*Rubus caesius* L.	вeдмeдики	little bears	Ukrainian		status-? bear food?	[202]
*Rubus caesius* L.	*Rubus caesius* L.	вeдмeжкa	bear plant	Ukrainian		status-? bear food?	[202]
*Rubus caesius* L.	*Rubus caesius* L.	видмид’i	bears	Ukrainian		status-? bear food?	[202]
*Rubus caesius* L.	*Rubus caesius* L.	видмeдиц’и	she-bears	Ukrainian	Volhynia	status-? bear food?	[215]
*Rubus caesius* L.	*Rubus caesius* L.	чepниця вeдмeжa	bear blackberry	Ukrainian, Central-Dnieper		status-? bear food?	[180]
*Rubus caesius* L.	*Rubus caesius* L.	мeдвeдинa	bear plant	Ukrainian, Central-Polessian		status-? bear food?	[180]
*Rubus caesius* L.	*Rubus caesius* L.	мeдвeдини	bear plant	Ukrainian, Central-Polessian		status-? bear food?	[180]
*Rubus caesius* L.	*Rubus caesius* L.	мeдвeжик	little bear	Ukrainian, Central-Polessian		status-? bear food?	[180]
*Rubus caesius* L.	*Rubus caesius* L.	мeдвeжинa	bear plant	Ukrainian, Central-Polessian		status-? bear food?	[180]
*Rubus caesius* L.	*Rubus caesius* L.	вeдмeдицi	she-bears	Ukrainian, West-Polessian		status-? bear food?	[180]
*Rubus caesius* L.	*Rubus caesius*	karuu-marja	bear-berry	Votic		shape and surface	[113]
*Rubus caesius* L.	*Rubus caesius* L.	karumarjõ, karumarje	bear berry(ies)	Votic		shape and surface	[56]
*Rubus caesius* L.	*Rubus caesius* L.	karumurak	bear cloudberry	Votic		shape and surface	[56]
*Rubus caesius* L.	*Rubus caesius* L.	karuvabarn	bear rapsberry	Votic		shape and surface	[56]
*Rubus chamaemorus* L.	*Rubus chamaemorus* L.	мeдвeжaник	bear plant	Central Russian	Tver	shape? bear food?	[57]
*Rubus chamaemorus* L.	*Rubus chamaemorus* L.	мeдвeжaтник	bear plant	Central Russian	Tver	shape? bear food?	[57]
*Rubus chamaemorus* L.	*Rubus chamaemorus* L.	мeдвeжaтник	bear plant	Russian	Middle Ob region	shape? bear food?	[101]
*Rubus chamaemorus* L.	*Rubus chamaemorus* L.	мeдвeжaтницa	bear plant	Russian	Middle Ob region	shape? bear food?	[101]
*Rubus corylifolius* Sm.	*Rubus corylifolius* Sm.	medveža	bear plant	Ukrainian		bear food	[130]
*Rubus corylifolius* Sm.	*Rubus corylifolius* Sm.	vedmežyna	bear plant	Ukrainian		bear food	[130]
*Rubus corylifolius* Sm.	*Rubus corylifolius* Sm.	vedmižyna	bear plant	Ukrainian		bear food	[130]
*Rubus idaeus* L.	*Rubus idaeus* L.	miricule de l'urse	bear blackberry	Italian, southern	Fara San Martino and Sant'Eufemia a Majella (Chieti), Abruzzo, central Italy	wild, status-	[90]
*Rubus idaeus* L.	*Rubus idaeus* L.	мeдвeжьи ягoды	bear berries	Russian	Argunj region	bear food	[57]
*Rubus idaeus* L.	*Rubus idaeus* L.	вeдмeжa ягoдa	bear berry	Ukrainian, speppe dialect		shape? bear food?	[180]
*Rubus laciniatus* Willd.	*Rubus lacimatus*	björnbär	bear berry	Swedish	Dalsland	?	[155]
*Rubus plicatus* Weihe & Nees	*Rubus fruticosus* L. agg.	karhunvatukka	bear rapsberry	Finnish		shape and surface	[82]
*Rubus plicatus* Weihe & Nees	*Rubus fruticosus* L. agg.	karhunmarja	bear berry	Finnish		shape and surface	[82]
*Rubus plicatus* Weihe & Nees	*Rubus fructicosus* L.	мeдвeдoк	little bear	Southern Russian	Orel gub.	surface? bear food?	[57]
*Rubus plicatus* Weihe & Nees	*Rubus plicatus*	björnbär	bear berry	Swedish	Dalarna, Värmland, Uppland, Västmanland, Öland, Östergötland, Dalsland, Västergötland, Bohuslän, Småland, Blekinge, Halland, Skåne	?	[155]
*Rubus plicatus* Weihe & Nees	*Rubus plicatus*	björnbärsbuske	bear berry bush	Swedish	Bohuslän, Småland	?	[155]
*Rubus plicatus* Weihe & Nees	*Rubus plicatus*	björnbärsris	bear berry shrub	Swedish	Bohuslän	?	[155]
*Rubus plicatus* Weihe & Nees	*Rubus plicatus*	björnbärstorn	bear berry thorn	Swedish	Bohuslän	?	[155]
*Rubus plicatus* Weihe & Nees	*Rubus plicatus*	björnhallon	bear raspberry	Swedish	Södermanland, Östergötland, Småland	?	[155]
*Rubus plicatus* Weihe & Nees	*Rubus fruticosus*	björnbär	bear berry	Swedish	Dalarna, Värmland, Västmanland, Öland, Dalsland, Västergötland, Bohuslän, Småland, Blekinge, Halland, Skåne, Estland Med Gsb	?	[155]
*Rubus plicatus* Weihe & Nees	*Rubus fruticosus*	björnbärsbuske	bear berry bush	Swedish	Bohuslän, Småland	?	[155]
*Rubus plicatus* Weihe & Nees	*Rubus fruticosus*	björnbärsris	bear berry shrub	Swedish	Bohuslän	?	[155]
*Rubus plicatus* Weihe & Nees	*Rubus fruticosus*	björnbärstorn	bear berry thorn	Swedish	Bohuslän	?	[155]
*Rubus plicatus* Weihe & Nees	*Rubus fruticosus*	björnhallon	bear raspberry	Swedish	Småland	?	[155]
*Rubus saxatilis* L.	*Rubus saxatilis* L.	karuvaarakud, karuvaarikas	bear rapsberry	Estonian	Saa	shape and surface	[58]
*Rubus saxatilis* L.	*Rubus saxatilis* L.	ciate d'ors; ciate de l'ors; sgranfi de l'ors	bear feet	Italian, northern	Trentino, north-eatern Italy	shape	[115]
*Rubus* spp.	x ??? *Rubus* sp.??	medveszödör	bear’s black berry	Hungarian	Szeged (Hungarian Plain)	?	[188]
*Rubus* spp.	*Rubus*	svillbjörnbär	swollen bear berry	Swedish	Bohuslän	?	[155]
*Rubus trivialis* Michx.	*Rubus nessensis* Hall	karuvabarnas, karuvaarak, karu-vabarn, kahru-vavarna	bear rapsberry	Estonian	Saa, Hls, Kod	shape and surface	([58, 73, 184, 185], Kalle and Sõukand unpublished)
*Rubus trivialis* Michx.	*Rubus nessensis* Hall	karumari	bear berry	Estonian		shape and surface	[185]
*Rubus trivialis* Michx.	*Rubus nessensis* W. Hall	вeдмiжинa	bear plant	Ukrainian	Ukraine	status-? bear food?	[180]
*Rubus trivialis* Michx.	*Rubus nessensis* W. Hall	мeдвeдинa	bear plant	Ukrainian, Central-Polessian		status-? bear food?	[180]
*Rubus trivialis* Michx.	*Rubus nessensis* W. Hall	мeдвeжa	bear plant	Ukrainian, Central-Polessian		status-? bear food?	[180]
*Rubus trivialis* Michx.	*Rubus nessensis* W. Hall	мeдвeжик	little bear	Ukrainian, Central-Polessian		status-? bear food?	[180]
*Rubus trivialis* Michx.	*Rubus nessensis* W. Hall	мeдвeжинa	bear plant	Ukrainian, Central-Polessian		status-? bear food?	[180]
*Rubus trivialis* Michx.	*Rubus nessensis* W. Hall.	вeдмeжинa	bear plant	Ukrainian, speppe dialect		status-? bear food?	[180]
*Rubus trivialis* Michx.	*Rubus nessensis* W. Hall	мeдвiдник	bear plant	Ukrainian, Volhynian		status-? bear food?	[180]
*Rubus* spp.	*Rubus subgenus* Rubus	niedźwiedziuchy	bear plant	Polish	Kraśnik, PL	bear food	unpublished Polish Ethnographic Atlas 1948, questionnaire 2
*Rubus* spp.	*Rubus subg*. Rubus	björnbär	bear berry	Swedish		folk etymology	[54]
*Rumex crispus* L.	*Rumex crispus* L.	karuein	bear grass	Estonian	Hel	size and shape	[58]
*Rumex crispus* L.	*Rumex crispus* L.	karuhaina, karuhain	bear grass	Estonian	Trv, Krl	size and shape	[58]
*Rumex crispus* L.	*Rumex crispus* L.	karuoblik	bear sorrel	Estonian	Kam	size and shape	[58]
*Rumex obtusifolius* L.	*Rumex obtus*	Bärentrappen	?	German, non specified	not specified	?	[65]
*Salix alba* L.	*Salix alba* L.	saucia ursarella	bear willow	Italian, southern	Valle Peligna, Abruzzo, central Italy	status-	Cianfaglione unpublished
*Salix triandra* L.	*Salix triandra* L.	saucia ursarella	bear willow	Italian, southern	Valle Peligna, Abruzzo, central Italy	status-	Cianfaglione unpublished
*Salix viminalis* L.	*Salix viminalis* L. (incl. hybrids)	saucia ursarella	bear willow	Italian, southern	Valle Peligna, Abruzzo, central Italy	status-	Cianfaglione unpublished
*Salvia aethiopis* L.	*Salvia aethiopis* L.	мeчo уxo	bear ear	Bulgarian	Dragoman & Breze villages, Sofia area	shape, surface	[86]
*Salvia aethiopis* L.	*Salvia aethiopis* L.	мeчa cтъпкa	bear step	Bulgarian		shape, surface?	[86]
*Salvia aethiopis* L.	*Salvia aethiopis* L.	мeчиje увo	bear ear	Serbo-Croat		shape, surface	[84]
*Salvia aethiopis* L.	*Salvia aethiopis* L.	вушкo вeдмeжe	bear ear	Ukrainian		shape? surface?	[180]
*Salvia aethiopis* L.	*Salvia aethiopis* L.	мeдвeжe вушкo	bear little ear	Ukrainian	Odessa, Donetsk	shape? surface?	[203]
*Salvia aethiopis* L.	*Salvia aethiopis* L.	вeдмeжe вуxo	bear ear	Ukrainian, Central-Dnieper, steppe dialect	Kharkov, Donetsk	shape? surface?	[180, 203]
*Salvia aethiopis* L.	*Salvia aethiopis* L.	мeдвeжe вуxo	bear ear	Ukrainian, Podolian, steppe dialect	Odessa, Transcarpathia	shape? surface?	[180, 203]
*Salvia aethiopis* L.	*Salvia aethiopis* L.	уxo мeдвeжe	bear ear	Ukrainian, steppe dialect		shape? surface?	[180]
*Salvia aethiopis* L.	*Salvia aethiopis* L.	мeдвeжa лaпa	bear paw	Ukrainian, steppe dialect	Donetsk	shape? surface?	[180, 203]
*Salvia aethiopis* L.	*Salvia aethiopis* L.	вeдмeжe уxo	bear ear	Ukrainian, steppe dialect, Podolian	Dnepropetrovsk	shape? surface?	[130, 219]
*Salvia sclarea* L.	*Salvia sclarea* L.	мeчa пeтa	bear heel	Bulgarian		shape, surface	[86]
*Salvia sclarea* L.	*Salvia sclarea* L.	мeчa cтъпкa	bear step	Bulgarian		shape, surface	[86]
*Salvia sclarea* L.	*Salvia sclarea* L.	medvije uho	bear ear	Croatian	Dalmacija	shape and surface?	[126, 138]
*Salvia sclarea* L.	*Salvia sclarea*	мeдвиje уxo	bear ear	Serbo-Croat		shape, surface	[84]
*Salvia sclarea* L.	*Salvia sclarea*	мeчje увo	bear ear	Serbo-Croat		shape, surface	[84]
*Salvia sclarea* L.	*Salvia sclarea* L.	ayı kulağı	bear ear	Turkish	S and W Anatolia	shape	[243]
*Sambucus ebulus* L.	*Sambucus ebulus* L.	ayı otu	bear herb	Turkish	İçel, Niğde (S Anatolia)	?	[146]
*Sambucus ebulus* L.	*Sambucus ebulus* L.	ayı otu	bear herb	Turkish	Bayramiç, Çanakkale (NW Anatolia)	?	[151]
*Sambucus ebulus* L.	*Sambucus ebulus* L.	ayı otu	bear herb	Turkish	Karaman, Mersin (S Anatolia)	?	[227]
*Sambucus ebulus* L.	*Sambucus ebulus* L.	ayıboğan	bear choker	Turkish	Kahramanmaraş (S Anatolia)	?	[227]
*Sambucus ebulus* L.	*Sambucus ebulus* L.	ayı otu	bear herb	Turkish	İçel, Niğde (S Anatolia)	?	[227]
*Sambucus racemosa* L.	*Sambucus racemosa* L.	karupihlakas	bear rowan	Estonian	Nrv	dangerousness	[58]
*Sambucus racemosa* L.	*Sambucus racemosa* L.	мeдвeжьe дepeвo	bear tree	Russian	Perm	status-?	[57]
*Sambucus racemosa* L.	*Sambucus racemosa* L.	мeдвeжья дудкa	bear pipe	Russian	Ural	status-?	[57]
*Sambucus racemosa* L.	*Sambucus racemosa* L.	мeдвeжник	bear plant	Russian	Ural	status-?	[57]
*Sambucus* spp.	*Sambucus* L.	мeдвeжья ягoдa	bear berry	Russian	Middle Ob region	status-?	[57]
*Sanicula elata* Buch.-Ham. ex D. Don.	*Sanicula europaea* L.	uracca d'ours	bear ear	Italian, northern	Bologna area, Emilia, northern Italy	shape	[74]
*Sarcodon imbricatus* (L.) P. Karst.	*Sarcodon imbricatus* (L.) P/Karst. (?)	мeдвeжий гpиб	bear mushroom	Russian	Perm	size, colour, inedibility	[102]
*Sedum* spp.	*Sedum* L.	мeчкинo гpoзьe	bear grapes	Bulgarian		?	[86]
*Selaginella* spp.	*Selaginella*	Bärläppchen	little bear club moss	German, non specified	not specified	surface?, shape?	[65]
*Sempervivum globiferum* L.	*Jovibara sobolifera Opiz*	karumunad	bear balls	Estonian	JMd	shape	[58]
*Sempervivum globiferum* L.	*Jovibara sobolifera Opiz*	karukellad	bear bells (meaning balls)	Estonian	JMd	shape	[58]
*Sempervivum tectorum* L.	*Sempervivum tectorum* L.	pane dell'orse	bear bread	Italian, southern	Rocca di Cambio (L'Aquila), Abruzzo, central Italy	bear food?	[90]
*Silaum silaus* (L.) Schinz & Thell.	*Silaus prat.*	falsche, unechte Bärenwurz	wrong bear root	German, non specified	not specified	bear food	[65]
*Silene chalcedonica* (L.) E.H.L. Krause	*Lychnis chalcedonica* L.	мeдвeжьe мылo	bear soap	Russian	Middle Ob region	?	[101]
*Silene dioica* (L.) Clairv.	*Silene dioica*	björnblomma	bear flower	Swedish	Norrbotten	?	[155]
*Sinapis arvensis* L.	*Sinapis arvensis* L.	karurõigas	bear radish	Estonian	Rõu	status-	[58]
*Smilax aspera* L.	*Smilax aspera* L. ?	aussara, autsàra, auttsàra, aursàra, attsara; ussarèddha	little female bear	Sardinian	Lanusei (Ogliastra), eastern Sardinia	?	[142]
*Solanum dulcamara* L.	*Solanum dulcamara* L.	мядзвeжыя ягaды	bear berries	Belarussian	Smol.	inedibility?	[79]
*Solanum dulcamara* L.	*Solanum dulcamara* L.	bjønnebær	bear berry	Norwegian		status-	[80]
*Solanum dulcamara* L.	*Solanum dulcamara* L.	мeдвeжьи ягoды	bear berries	Southern Russian	Smolensk	inedibility?	[57]
*Solanum pseudopersicum* Pojark.	*Solanum persicum* Will.	мeдвeжьи ягoды	bear berries	Russian	Siberia	inedibility?	[116]
*Solanum tuberosum* L.	*Solanum tuberosum*	björnpära	bear potatoes	Swedish	Jämtland	status-	[155]
*Solanum tuberosum* L.	*Solanum tuberosum*	björnpäron	bear potatoes	Swedish	Blekinge	status-	[155]
*Soldanella* spp.	*Soldanella* spp.	recchietta d'urs	little bear ear	Italian, southern	Alto Sangro, Marsica, Valle Peligna, Majella surroundings, Gran Sasso d'Italia and Laga Mt., Abuzzo, central Italy	shape	Cianfaglione unpublished
*Solenophora* Benth.	*Solenophora* Benth. (Arctocalyx Fenzl)	medvjeđača	bear plant	Croatian	no location	?	[126, 216]
*Sonchus arvensis* L.	*Sonchus arvensis*	medvesaláta	bear’s lattice	Hungarian	Gyimes (Transylvania)	bear food	[97, 213]
*Sorbus aria* (L.) Crantz	*Sorbus aria* L.	fave de l'ors	bear beans	Italian, northern	Predazzo, Trentino, north-eastern Italy	bear food?	[115]
*Sorbus aria* (L.) Crantz	*Sorbus aria* L.	pan d'orso; pan d'ors	bear bread	Italian, northern	Verona area, Veneto and Trentino, north-eastern Italy	bear food	[74, 115]
*Sorbus aucuparia* L.	*Sorbus auc.*	Bärwid	?	German, non specified	not specified	bear food	[65]
*Sorbus aucuparia* L.	*Sorbus aucuparia*	мeдвeдoвa тpeшњa	bear sweet cherry	Serbo-Croat		status-?	[84]
*Sorbus aucuparia* L.	*Sorbus aucuparia* L.	ayı ovazı	bear rowan	Turkish	Karaman (S Anatolia)	status -	[227]
Sorbus spp.	*Sorbus (bunch of berries)*	мeдвeдкo	bear	Northern Russian	Arkhangelsk, Karelia	status -?	[57]
*Sorbus torminalis* (L.) Crantz	*Sorbus torminalis* Crtz.	мeдвeжья гpушa	bear pear	Russian		translated from Tata	[116]
*Sphagnum* spp.	*Sphagnum* ssp.	karusammal	bear moss	Estonian		folk etymology	[56]
*Sphagnum* spp.	*Sphagnum*	Bärenmoos	bear moss	German, non specified	not specified	surface?	[65]
*Spiraea media* Schmidt	*Spiraea media* (W. et Kit.) Schmidt.	вeдмeжья ягoдa	bear berry	Russian	Middle Ob region	inedibility?	[101]
*Spiraea ulmifolia* Scop.	*Spiraea ulmifolia*	medvejka	bear plant	Slovenian		bear food?	[84]
*Spiraea ulmifolia* Scop.	*Spiraea ulmifolia*	medvejno latje	bear stalks	Slovenian		bear food?	[84]
*Styrax officinalis* L.	*Styrax officinalis* L.	ayıfındığı	bear hazelnut	Turkish	Aegean Region of Anatolia	status -	[158]
*Styrax officinalis* L.	*Styrax officinalis* L.	ayıfındığı	bear hazelnut	Turkish	Antalya (SW Anatolia)	status -	[159]
*Succisa pratensis* Moench	*Succisa pratensis* Moench	lilla karunupp	lilack bear bud	Estonian	Vig	surface	[58]
*Suillellus luridus* (Schaeff.) Murrill	Гpиб пoддубник	мeдвeжий гpиб	bear mushroom	Northern Russian	the Northern Dvina region	?	[57]
*Zosima orientalis* Hoffm.	*Zosima absinthifolia* (Vent.) Link	ayıeli	bear hand	Turkish	Anatolia	shape	[146]
*Tanacetum parthenium* (L.) Sch. Bip.	*Chrysanthemum parth.*	Bärenmutter-kraut	bear mother herb	German, non specified	not specified	folk etymology?	[65]
*Tanacetum vulgare* L.	*Tanacetum vulgare* L.	мeдвeжьe уxo	bear ear	Russian	Perm	shape?	[57]
*Taraxacum officinale* F.H. Wigg.	*Taraxacum off.*	Bärenzahn	bear tooth	German, non specified	not specified	shape	[65]
*Telekia speciosa* (Schreb.) Baumg.	*Telekia speciosa*	medvesaláta	bear’s lattice	Hungarian	Gyimes (Transylvania)	bear food	[97, 213]
*Tofieldia pusilla* (Michx.) Pers.	*Tofieldia pusilla* (Michx.) Pers.	karhunheinä, pohjan karhunheinä, soiden karhunruoho	bear grass	Finnish	many written records	?	[82]
*Tremiscus helvelloides* (DC.) Donk	*Guepinia rufa* (Jacq.) Beck	reccia d'orso	bear ear	Italian, northern	Verona area, Veneto, northern Italy	shape	[74]
*Trichophorum cespitosum* (L.) Hartm.	*Scirpus caespitsus* L.	bjønnskjegg	bear beard	Norwegian	Bygland, Evje, Froland, Bakke	?	[80]
*Trifolium pannonicum* (Jacq.) Dobrocz.	*Trifolium pannonicum* (Jacq.) Dobrocz.	ayı kulağı	bear ear	Turkish	Anatolia	size	[226]
*Trifolium* spp.	*Trifolium*	ayubaši	bear head	Tatar		shape	[171]
*Trifolium* spp.	*Trifolium* sp.	ayu bašï	bear’s head	Tatar		shape	[6]
*Triglochin* spp.	*Triglochin* L.	barica	she-bear	Upper-Sorbian		?	[63]
*Triglochin* spp.	*Triglochin* L.	barička	she-bear	Upper-Sorbian		?	[63]
*Turgenia latifolia* (L.) Hoffm.	*Laserpitium latifolium*	medvedovka	bear plant	Slovenian		?	[84]
*Turgenia latifolia* (L.) Hoffm.	*Laserpitium latifolium*	björnlabb	bear paw	Swedish	Gotland	?	[155]
*Turritis glabra* L.	*Turritis glabra* L.	мeчo зeлe	bear cabbage	Bulgarian	Vraca	?	[86]
*Tussilago farfara* L.	*Tussilago farfara* L.	мeчa cтъпкa	bear step	Bulgarian	Kolarovgrad	shape, surface	[86]
*Tussilago farfara* L.	*Tussilago farfara* L.	karhunkämmenheenä	bear palm's grass	Finnish	Konn	surface	[82]
*Tussilago farfara* L.	*Tussilago farfara*	ayıkulağı	bear ear	Turkish	Bayramiç, Çanakkale (NW Anatolia)	shape	[151]
*Typha angustifolia* L.	*Typha angustifolia and Typha latifolia*	meškakūlė	bear reed/tube, rod	Lithuanian	Lithuania	surface	[87, 199]
*Typha latifolia* L.	*Typha latifolia* L.	karukolgid	bear club (smasher)	Estonian	Hls	shape and surface	[58]
*Typha latifolia* L.	*Typha latifolia* L.	karunuiad	bear club (smasher)	Estonian	Nrv	shape and surface	[58]
*Typha latifolia* L.	*Typha lat.*	Bärenknüppel	bear bludgeon	German, non specified	not specified	surface, folklore?	[65]
*Typha latifolia* L.	*Typha angustifolia and Typha latifolia*	meškakūlė	bear reed/tube, rod	Lithuanian	Lithuania	surface	[87, 199]
*Typha latifolia* L.	*Typha latifolia*	meškakūlė	bear reed/tube, rod	Lithuanian	Lithuania	surface, size, shape	[87, 199]
*Ursinia* spp.	*Ursinia*	Bärenkamille	bear camomille	German, non specified	not specified	Latin calque	[65]
*Ursinia* spp.	*Ursinia*	Bärenkraut	bear herb	German, non specified	not specified	Latin calque	[65]
*Urtica dioica* L.	*Urtica dioica* L.	karunõges	bear nettle	Estonian	Kod	surface	[58]
*Vaccinium myrtillus* L.	*Vaccinium myrtillus*	medveszeder	bear’s black berry	Hungarian	Székelyföld (Transylvania)	bear food	[221]
*Vaccinium myrtillus* L.	*Vaccinium myrtillus* L.	bjønnebær	bear berry	Norwegian		bear food?	[80]
*Vaccinium myrtillus* L.	*Vaccinium myrtillus*	bïernenmuerjie	bear berry	South Saami		bear food?	[170]
*Vaccinium myrtillus* L.	*Vaccinium myrtillus* L.	björnbär	bear berries	Swedish	Lapland, Ångermanland, Hälsingland	status-	[15]
*Vaccinium myrtillus* L.	*Vaccinium myrtillus*	björnbär	bear berry	Swedish	Lappland, Hälsingland, Härjedalen, Dalarna, Västmanland	status-	[155]
*Vaccinium myrtillus* L.	*Vaccinium myrtillus*	björnbärsris	bear berry shrub	Swedish	Hälsingland	status-	[155]
*Vaccinium myrtillus* L.	*Vaccinium myrtillus f. epuinosum*	björnbär	bear berry	Swedish	Härjedalen, Västerbotten	status-	[154, 155]
*Vaccinium myrtillus* L.	*Vaccinium myrtillus* L.	byönnber	bear berry	Swedish	Älvdalen	folklore	[223]
*Vaccinium myrtillus* L.	*Vaccinium myrtillus* L.	ayı üzümü	bear grape	Turkish	Anatolia	bear food	[199]
*Vaccinium oxycoccos* L.	*Vaccinium ocycoccus* L.	bjørnebær	bear berry	Norwegian	here and there in Norway	bear food?	[80]
*Vaccinium oxycoccos* L.	*Vaccinium oxycoccos*	bïernenmuerjie	bear berry	South Saami		bear food?	[170]
*Vaccinium oxycoccos* L.	*Vaccinium oxycoccus*	björnbär	bear berry	Swedish	Ångermanland, Eastern Jämtland	status-	[15]
*Vaccinium oxycoccos* L.	*Vaccinium oxycoccos*	björnbär	bear berry	Swedish	Norrbotten, Jämtland	status-	[155]
*Vaccinium uliginosum* L.	*Vaccinium uliginosum*	bjørnbær	bear berry	Norwegian	Trønd	?	[233]
*Vaccinium uliginosum* L.	*Vaccinium uliginosum* L.	bjønnebær	bear berry	Norwegian		bear food?	[80]
*Vaccinium uliginosum* L.	*Vaccinium uliginosum*	bïernenmuerjie	bear berry	South Saami		bear food?	[170]
*Vaccinium uliginosum* L.	*Vaccinium uliginosum*	björnbär	bear berry	Swedish	Ångermanland	status-	[15]
*Vaccinium uliginosum* L.	*Vaccinium uliginosum* L.	björnbär	bear berry	Swedish	Medelpad	status-	[155]
*Vaccinium vitis-idaea* L.	*Vaccinium vitis-idaea* L.	pomati d'ors	bear fruit	Italian, northern	Trento area, Trentino, north-eatern Italy	bear food?	[115]
*Vaccinium vitis-idaea* L.	*Vaccinium vitis-idaea* L.	ciate d'ors	bear feet	Italian, northern	Trentino, north-eatern Italy	shape	[115]
*Vaccinium vitis-idaea* L.	*Vaccinium vitis idaea*	мeчкинo гpoзje	bear grapes	Macedonian	Macedonia	status-? bear food?	[84]
*Vaccinium vitis-idaea* L.	*Vaccinium vitis-idaea* L.	мeдвeдзoвo гpoзнo	bear grapes	Ruthenian	Vojvodina	status-? bear food?	[125]
*Vaccinium vitis-idaea* L.	*Vaccinium vitis idaea*	мeдвeђe гpoжђe	bear grapes	Serbo-Croat		shape? bear food?	[84]
*Vaccinium vitis-idaea* L.	*Vaccinium vitis idaea*	мeчje гpoжђe	bear grapes	Serbo-Croat		shape? bear food?	[84]
*Veratrum album* L.	*Veratrum album*	medvekocsány	bear’s stalk (bear peduncle)	Hungarian	Hétfalu (Transylvania)	size	[200]
*Veratrum lobelianum* Bernh.	*Veratrum lobelianum* Bernh.	мeчкинa тpeвa	bear grass	Bulgarian		?	[86]
*Veratrum* spp.	*Veratrum* L.	мeчкинa тpeвa	bear grass	Bulgarian		?	[86]
*Verbascum densiflorum* Bertol.	*Verbascum thapsiforme* Schrad	Schrad. уxo	bear ear	Bulgarian	Stara zagora	shape, surface	[86]
*Verbascum densiflorum* Bertol.	*Verbascum thapsiforme* Schrad.	мeдвeжьe уxo	bear ear	Central, Southern Russian	Moscow, Vladimir, Nizhni Novgorod, Kazan, Orel, Kursk	shape, surface	[57]
*Verbascum densiflorum* Bertol.	*Verbascum densiflorum* Bertol.	вушкo мeдвeжe	bear little ear	Ukrainian		shape, surface?	[180]
*Verbascum densiflorum* Bertol.	*Verbascum densiflorum* Bertol.	вушкo мeдвeжaчe	bear little ear	Ukrainian		shape, surface?	[180]
*Verbascum densiflorum* Bertol.	*Verbascum densiflorum* Bertol.	уxo вeдмeжe	bear ear	Ukrainian		shape, surface?	[180]
*Verbascum densiflorum* Bertol.	*Verbascum thapsiforme* Schrad.	мeдвeжaчe вуxo	bear ear	Ukrainian		shape, surface?	[219]
*Verbascum densiflorum* Bertol.	*Verbascum thapsiforme* Schrad.	мeдвeжe вушкo	bear little ear	Ukrainian		shape, surface?	[219]
*Verbascum densiflorum* Bertol.	*Verbascum densiflorum* Bertol.	уxo мeдвeжe	bear ear	Ukrainian, Central-Dnieper		shape, surface?	[180]
*Verbascum lychnitis* L.	*Verbascum lychnitis* L.	вeдмeжe вуxo	bear ear	Ukrainian		shape, surface?	[219]
*Verbascum lychnitis* L.	*Verbascum lychnitis* L.	vedmeže uxo	bear ear	Ukrainian, steppe dialect		shape, surface?	[130, 180]
*Verbascum lychnitis* L.	*Verbascum lychnitis* L.	мeдвeжe вушкo	bear little ear	Ukrainian, steppe dialect, East-Polessian, Dniester, Transcarpathian		shape, surface?	[180, 219]
*Verbascum lydium* Boiss. var. heterandrum Murb.	*Verbascum lydium* Boiss. var. heterandrum Murb.	ayıkulağı	bear ear	Turkish	Muğla (SW Anatolia)	shape	[92]
*Verbascum nigrum* L.	*Verbascum nigrum* L.	мeдвeжьe уxo	bear ear	Central Russian	Pskov	shape, surface	[57]
*Verbascum nigrum* L.	*Verbascum nigrum* L.	уxo мeдвeдячe	bear ear	Ukrainian, Transcarpathian		shape, surface?	[180]
*Verbascum phlomoides* L.	*Verbascum phlomoides* L.	мeдвeжьe уxo	bear ear	Russian		shape, surface?	[116]
*Verbascum phlomoides* L.	*Verbascum phlomoides* L.	medviže uxo	bear ear	Ukrainian		shape, surface?	[130]
*Verbascum phlomoides* L.	*Verbascum phlomoides* L.	vedmeže uxo	bear ear	Ukrainian		shape, surface?	[130]
*Verbascum phlomoides* L.	*Verbascum phlomoides* L.	мeдвeжe ушкo	bear little ear	Ukrainian		shape, surface?	[219]
*Verbascum phlomoides* L.	*Verbascum phlomoides* L.	vedmeže vuxo	bear ear	Ukrainian, Central-Dnieper, steppe dialect		shape, surface?	[130, 180]
*Verbascum phlomoides* L.	*Verbascum phlomoides* L.	мeдвeжe oкo	bear eye	Ukrainian, East-Polessian	Chernigov	shape, surface?	[180, 203]
*Verbascum phlomoides* L.	*Verbascum phlomoides* L.	вуxo мeдвeжe	bear ear	Ukrainian, Gutsulian		shape, surface?	[180]
*Verbascum phlomoides* L.	*Verbascum phlomoides* L.	мeдвeжe уxo	bear ear	Ukrainian, Transcarpathian		shape, surface?	[130, 180, 219]
*Verbascum speciosum* Schrad.	*Verbascum speciosum* Schrad.	ayı lahanası	bear cabbage	Turkish	Iğdır (E Anatolia)	shape, status-	[140]
*Verbascum speciosum* Schrad.	*Verbascum speciosum* Schrad.	ayıkulağı	bear ear	Turkish	Tekirdağ (European part of Turkey)	shape	[227]
*Verbascum* spp.	*Verbascum*	мядзвeдзeвa вуxa	bear ear	Belarussian	Gomel.	shape, surface	[79]
*Verbascum* spp.	*Verbascum*	мядзвeжae вушкa/вушкi	bear little ears	Belarussian	Gomel., Mogil.	shape, surface	[79]
*Verbascum* spp.	*Verbascum*	мядзвeдзeвa вуxa	bear ear	Belarussian	Gomel.	shape, surface	[79]
*Verbascum* spp.	*Verbascum*	мядзвeжжы вушы	bear ear	Belarussian	Viteb.	shape, surface	[79]
*Verbascum* spp.	*Verbascum* L.	мeчe уxo	bear ear	Bulgarian		shape, surface?	[86]
*Verbascum* spp.	*Verbascum*	Bärenkraut	bear herb	German, non specified	not specified	surface?	[65]
*Verbascum* spp.	*Verbascum* sp. [?]	мeдвeжьи кocы	bear plaits	Russian	Ural	size?	[57]
*Verbascum* spp.	*Verbascum*	мeдвeд-уxo	bear ear	Serbo-Croat		shape	[84]
*Verbascum* spp.	*Verbascum* L.	medved’ače uxo	bear ear	Ukrainian		shape, surface?	[130]
*Verbascum* spp.	*Verbascum* L.	vedmeže uxo	bear ear	Ukrainian		shape, surface?	[130]
*Verbascum thapsus* L.	*Verbascum thapsus*	ayïġulaghï	bear ear	Azeri		shape	[171]
*Verbascum thapsus* L.	*Verbascum thapsus* L.	мeдвeжник мeлкий	little bear plant	Central Russian	Nizhni Novgorod	shape, surface	[57]
*Verbascum thapsus* L.	*Verbascum thapsus*	upahalhi	bear ear	Chuvash		shape	[171]
*Verbascum thapsus* L.	*Verbascum thapsus*	ayuwqulaq	bear ear	Crimean Tatar		shape	[171]
*Verbascum thapsus* L.	*Verbascum thapsus* L.	karuleht	bear leaves	Estonian		surface	[64, 182]
*Verbascum thapsus* L.	*Verbascum thapsus* L.	karuhein	bear grass	Estonian		surface	[201]
*Verbascum thapsus* L.	*Verbascum thapsus* L.	lambakaruleht, lammas-karulehed, lammaskaruleht	lamb('s)bear leave(s)	Estonian		surface	[64, 73]
*Verbascum thapsus* L.	*Verbascum thapsus*	Bärenfackel	bear torch	German, non specified	not specified	folklore?	[65]
*Verbascum thapsus* L.	*Verbascum thapsus*	ayuwqulaq	bear ear	Kazakh		shape	[171]
*Verbascum thapsus* L.	*Verbascum thapsus*	ayuwqulaq	bear ear	Kirghiz		shape?	[171]
*Verbascum thapsus* L.	*Verbascum thapsus* L.	мeдвeжий цвeт	bear flower	Northern Russian	Saratov	shape	[57]
*Verbascum thapsus* L.	*Verbascum thapsus* L.	мeдвeжья лaпa	bear paw	Russian		shape, surface?	[116]
*Verbascum thapsus* L.	*Verbascum thapsus* L.	мeдвeжьe уxo	bear ear	Russian	Middle Ob region	shape, surface?	[101]
*Verbascum thapsus* L.	*Verbascum thapsus* L.	мeдвeжьи ушки	bear little ears	Russian	Middle Ob region	shape, surface?	[101]
*Verbascum thapsus* L.	*Verbascum thapsus* L.	мeдвeжьи ушки	bear little ears	Russian	Ural	shape	[181]
*Verbascum thapsus* L.	*Verbascum thapsus* L.	мeдвeжья тpocть	bear walking stick	Russian	Ural	size?	[57]
*Verbascum thapsus* L.	*Verbascum thapsus*	ayuqolaq	bear ear	Tatar		shape	[171]
*Verbascum thapsus* L.	*Verbascum thapsus* L.	дивинa вeдмeжa	bear mullein	Ukrainian		shape, surface?	[180]
*Verbascum thapsus* L.	*Verbascum thapsus* L.	medved’ače uxo	bear ear	Ukrainian		shape, surface?	[130]
*Verbascum thapsus* L.	*Verbascum thapsus* L.	medveže uxo	bear ear	Ukrainian		shape, surface?	[130]
*Verbascum thapsus* L.	*Verbascum thapsus* L.	medveže uško	bear little ear	Ukrainian		shape, surface?	[130]
*Verbascum thapsus* L.	*Verbascum thapsus* L.	вeдмeжe уxo	bear ear	Ukrainian	Kiev	shape, surface?	[219]
*Verbascum thapsus* L.	*Verbascum thapsus* L.	вeдмeжe вуxo	bear ear	Ukrainian		shape, surface?	[219]
*Viburnum lantana* L.	*Viburnum lant.*	Barendreck	bear dirt	German, non specified	not specified	shape?	[65]
*Viburnum opulus* L.	*Viburnum opulus*	medveszőlő	bear’s grape	Hungarian	Sóvidék (Transylvania)	inedibility	[164]
*Viburnum opulus* L.	*Viburnum opulus*	medvekokojza	bear’s Vaccinium	Hungarian	Gyergyói-medence, a rare name (Transylvania)	inedibility	[97]
*Vicia dumetorum* L.	*Vicia dum.*	Bärenwicke	bear vetch	German, non specified	not specified	wild	[65]
*Vicia sepium* L.	*Vicia sepium* L.	meškažirnis	bear pea	Lithuanian	Lithuania	bear food	[87, 96]
*Vinca minor* L.	*Vinca minor*	Bärwinkel, Barwinkel	?	German, non specified	not specified	?	[65]
*Vitex agnus-castus* L.	*Vitex agnus-castus* L.	ayıbebesi	bear baby	Turkish	Bayramiç, Çanakkale (NW Anatolia)	?	[151]
*Xanthium orientale* L. subsp. italicum (Moretti) Greuter	*Xanthium orientale L. subsp. italicum* (Moretti) Greuter	ayı pıtırağı	bear cocklebur	Turkish	Yalova (NW Anatolia)	size	[227]
*Yucca* spp.	*Yucca*	Bärengras	bear grass	German, non specified	not specified	?	[65]

### Are all bears in plant names really bears?

There is a particular problem in the interpretation of the *bear* plant names. The bramble (*Rubus* subg. *Rubus*) was called *björnbär* [“bear berry”] in Swedish. This plant name was recorded already in 1643 in Sweden. But the reasoning behind this name is not easily clarified. There is nothing bear-like about it, and bears never appear in the area where it grows along the southern Swedish coasts. One possible explanation is that another folk name is *brumbär*, and the first part *brum* has maybe been associated with the animal’s name (although *brum* in this case actually means ‘leaf’, cf. English *bramble*, German *Brombeere*, etc.). Although most contemporary people connect *björnbär* with bears, it is not etymologically derived from historical roots connected to the animal bear [17]. On the other hand, the connections of *Rubus* spp. with bears in Belorussian, Ukrainian, Czech, Old-Polish, German, Italian, Estonian, and Votic cannot be explained by folk etymology.

Another example is the English name *bearbind* for *Clematis vitalba* L., *Convolvulus arvensis* L., and *Polygonum convolvulus* L., where the ‘bear’ element historically comes from Old English *beow* ‘barley’ rather than the Old English *bera* ‘bear’, and thus the whole name literally means the plant that binds barley [45, 46]. Similarly, it is *bere* (also ‘barley’) rather than *bera*, that lies behind *bear-barley*, a Northumberland name for *Hordeum hexastichon* or *tetrastichon*, which thus has nothing to do with bears, but literally means “barley-barley” [47].

Thiselton-Dyer [1] used *bear’s-wort* as an example, which, according to him, “is rather to be derived from its use in uterine complaints than from the animal”. The explanation makes a lot of sense as Eng. *bear* has (at least) two meanings, including ‘the animal bear’ and ‘to give birth’, which lent good material to folk etymologists. If there was an herb that helped giving birth or was good for gynecological disorders, it could get the name of bear’s-wort. The situation was the same with Germ. *Bär* ‘the animal bear’ and *gebären* ‘to give birth’.

Words from which the later words were derived are a critical issue also in other quasi bear plant names. The main similarities between Est. *karu* ‘bear’ and the adjective *karune* ‘hairy’ made contemporary speakers take them as being cognates. The words for ‘bear’ (North Estonian and standard Estonian *karu*, South Estonian *kahr*, and Finnish *karhu*) are related to each other. They are all connected with an ancient adjective (Est. *kare*, Fin. *karhea* ‘rough’), which may be a very old Indo-Iranian loan. The words for bear coincided phonetically with another word, *karune* ‘hairy’ (where -*ne* is a suffix of an adjective). This word is etymologically connected with the noun *karv* ‘hair’, which is an old Baltic loan in the Baltic-Finnish languages. Gooseberry is a good example here, which in some parts of Estonia was named *karusmari* (Est. *mari* ‘berry’, where the ‘s’ is a remnant of a genitive of the adjective suffix). It is of course wrong to understand this as “bear berry”; it is “hairy berry”. Another Estonian name is *karuohakas* (*Anchusa officinalis* L., *Cirsium lanceolatum* Scop.) which have nothing to do with bear either (*karu*, *karune* and *karv* [48]). Still, all the aforementioned and similar cases were included into Table 1 and considered during counting the number of phytonyms.

### The bear as compound in Eurasian plant names

Folk and scientific plant names have entered into intricate relationships. So, in Russia the first medicinal books (containing descriptions of various plants features) were translated in the 16th century from German and Polish, and later from Latin. Since then, a complex of natural-science ideas (having antique, Byzantine, and West-European origins) and oral folklore-mythological ideas has existed, with folk herbal books somewhere between them [49]. When the interpreters could not find an appropriate Russian equivalent, they used calques (loan translations, literal translations) or simple transliterations. This method was used also in alphabetical books (Rus. *aзбукoвники*) – the first explanatory dictionaries [50]. In Europe, “the fathers of botany” had to widen the lists of the antique authors at the expense of the local plant names [51]. Linnaeus’s system influenced national nomenclatures as well. Besides, there were also foreign names borrowed by noble classes through the books, and by the peasantry – via everyday contacts with neighbouring peoples. Nowadays, strong influence is exerted by school education and media.

Children played an important role in naming of local plants. They have been very observant of details and some of these names survive for generations (cf. [52, 53]). One example is the number of names for the sporangium of the golden maiden hair (*Polytrichum commune* L.) in the Germanic languages. Animal prefixes were very common (birds, mammals) and the children have seen moss as a field with crop and therefore named it *crow’s field*, *cuckoo’s cereals*, *fox rye*, etc. [54]. There was also a bear name for the *Polytrichum commune* L. from the northern Dalecarlia in Sweden: *björnblomma* [“bear flower”] [22].

While analyzing the “bear names” of plants in various languages, one may notice that the same element of a name – ‘bear’ – was motivated by a whole number of various plant features. Not all of them were productive to the same extent; still, the influence of each feature may be found all over the territory we studied.

It was not always possible to indicate with certainty one specific feature for each plant nomination. As it is shown in Table 2, various authors explain the bear-names of the same plant by different motivations. Very often, a name was the result of influence of a whole number of features. Moreover, one plant taxa can have various bear-phytonyms motivated by different features, for example among the 18 plants most often attributed bear-related phytonyms, we can detect between two to six different motivations, differing within the languages (Table 2). In the sections below, the names were grouped according to the “leading” assumed feature, while the other(s) was/were mentioned if relevant.

**Table 2:** The twenty most listed taxa and those languages/territories having the most popular motivations for their bear-related phytonyms

Species/motivation model	size	surface	form	poisioness/danger	inedibility/lower status	place of growing, wild	bears’ food	motif/belief	Latin calques
*Arctostaphylos uva-ursi* (L.) Spreng.			Az, It, Pol, Lit, Turk		Est, It, Hung, Lat	Hung, It	Alb, Bel, Cz, It, Lith, Pol, Rus, Ruth, Slov, Turk, Ukr		Bulg, Cr, Cz, Dan, Est, Ger, Izh, Rus, Norw, Sloven, Sw, Ukr/(?) Cr, Cz, Rus, S-C, Slov, Slov, Sorb, Ukr, Lat
*Heracleum sphondylium* L. incl *Heracleum sphondylium* subsp. *sibiricum* (L.) Simonk.	Hung, Fin, Vot	Blg, Eng, Est, Fin, IF, Izh, Vot	Chuv, Hung, Pol. Bulg/(?) Germ		It		Ger		Az, Bash, Cr, Cz, Dan, Pol, Rus, S-C, Slov, Slov, Tat, Sorb/(?) Ruth, Sorb, S-C
*Rubus caesius* L.		Est, Vot	Germ, Est, Vot		Bel, Cz, It, Ukr	It, Lith	Bel, Cz, Ukr, Lith		
*Acanthus mollis* L.			Turk/(?) Germ						Uz/(?) Ruth, S-C, Slov, Sorb
*Allium ursinum* L.					It	Hung, It, Turk	Alb, Germ, Lith		Az, Bash, Bulg, Dan, Est, Kirg, Rus, Turk, Ukr, Lith/(?) Pol, Ruth, S-C, Slov
*Lycopodium clavatum* L.		Cz, Hung, It/(?) Sw, Cz, Ger	Est, Pol/(?) Cz, Germ		Lith		Lith		
*Verbascum thapsus* L.	(?) Rus	Est/(?) Rus, Ukr	Az, Chuv, Cr-Tat, Kaz, Rus, Tat, Rus, Ukr/(?) Kirg					(?) Ger	
*Angelica sylvestris* L.	Fin, IF, Izh, Rus, Sw		Sw				Sw/(?) Ru	Fin, IF, Izh	
*Meum athamanticum* Garsault			Turk/(?) Ger				Ger		Rus
*Primula auricula* L.			Eng, It/(?) Ger						Bulg, Cr, Dan, Fr, S-C, Ukr/(?) Cz, Rus
*Angelica archangelica* L.		Fin, Ingr, Izh/(?) Rus, Sw						Fin, Ingr, Izh	
*Polytrichum commune* Hedw.		Rus	Est, Fin, Izh, Lith, Sw, Vot/(?) Germ						
*Empetrum nigrum* L.					Sw, Rus	Rus	Lith/(?) Norw, Sw		
*Geum rivale* L.		Est, IF, Izh			Sw				
*Paris quadrifolia* L.				Est, Fin, Lith/(?) Rus	Norw, Sw,	Lith/(?) Bulg			
*Crataegus monogyna* Jacq.					(?) Slov	(?) It	It		
*Equisetum arvense* L.			Est, Hung, Bulg, Lith			Lit			

(?) – motivation hypothesized, as not confirmed by the source

**Table 3:** Frequency of different phytonyms related to bears for the most named taxa in the “bear-richest” languages

Taxa/languages	Est	Germ	Rus	Sw	Turk	It	Ukr	S-C	Bulg	Sloven	Cz	Cr	Lith	Fin	Bel	Norw	Eng	Hung	Sorb
*Heracleum sphondylium* L. incl. *Heracleum sibiricum* L.	5	14	1	3				3	2	2	4	4		3			1	3	4
*Arctostaphylos uva-ursi* (L.) Spreng.	8	4	5	1	1	7	4	5	1	3	3	3	1		1	1		1	2
*Rubus caesius* L.	3	1		3		1	5				3		1		2				
*Acanthus mollis* L.		1			1	1		3		1	2	3							2
*Allium ursinum* L.	2	2	2		1	1	1	1	1	1			1					2	
*Lycopodium clavatum* L.	1	5		1		3					2		1					1	
*Primula auricula* L.		2	1			1	1	1	1		1	1					1		
*Polytrichum commune* Hedw.	1	1	1	2									1	1		1			
*Empetrum nigrum* L.	3		1	1									1	2		1			
*Paris quadrifolia* L.	2		1	1					2				1	1		1			
*Arctous alpina* (L.) Nied.		1		1		1										1			1
*Verbascum* spp.		1	1				1	1	1						2				
*Equisetum arvense* L.	1			1					1				1					3	
*Heracleum* spp.	1			2		1			1			1							
*Calla palustris* L.	1		1												1	1	1		

Only those languages are included with at least 20 records of bear-related phytonyms, and only those taxa that have bear-related phytonyms in at least five different languages among these. The numbers show the number of different meanings bear-related phytonyms have for a specific plant in the corresponding language. In those cases where the two names have a similar meaning, but different words are used to express it, they are considered as different. Abbreviations: Est – Estonian, Germ – German, Rus – Russian, Sw – Swedish, Turk – Turkish, Ukr – Ukrainian, It – Italian. S-C – Serbo-Croat, Bulg – Bulgarian, Sloven – Slovenian, Cz – Czech, Cr – Croatian, Lith – Lithuanian, Fin – Finnish, Hung – Hungarian, Norw – Norwegian, Bel – Belarussian, Eng – English, Sorb – Sorbian

From the point of view of word-formative models, we may conclude that bear-names might be coined in the following ways: 1) “bear/she-bear” (transfer of meaning), 2) bear + a body part of the bear (e.g. [“bear’s paw”]), 3) bear + a part of the plant (e.g. [“bear berry”]), 4) bear + a plant name (e.g. [“bear willow”]), 5) bear + a plant group name (e.g. [“bear tree”]), 6) bear + an object name (e.g. [“bear spindle”]).

**Fig. 3:**
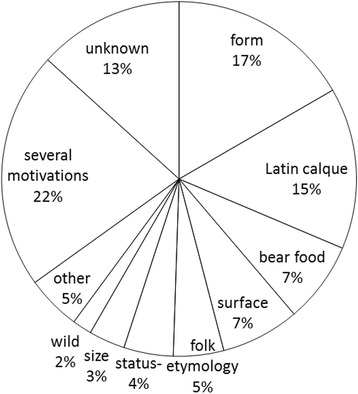
Proportional division of motivations. Both motivations identified as the source and also hypothesized are counted here

The plant taxa having most various phytonyms within regions/languages with rich representation of bear-related phytonyms were outlined in the Table 3. Although roughly one third of the motivations were hypothesized, the general proportion of the motivations is in large outlined in Fig. 3. The motivations below are listed based on the logical assumptions of the authors about the most popular motivations, yet the analysis proved such assumptions were incorrect.

#### Nomination by size

This nomination might be presumed to be the largest group, but in fact contributed only 3% of recorded or assumed motivations and some more in the combinations with other motivations. Bear names were given, as a rule, to large plants with high stalks and/or large leaves, other parts of unusually big size. Into this group such names might be included as Fin. *karhunputk* [“bear’s thistle”], Izh. *karhuntruba* [“bear’s pipe”] *Angelica archangelica* L. [55, 56]; Rus. Tobol. *мeдвeжьe дepeвo* [“bear’s tree”] *Asparagus* [57]; Est. *karuein*, *karuhaina*, *karuoblik* [“bear’s grass”, “bear’s sorrel”] *Rumex crispus* L. [58]. An unidentified broad-leaved meadow plant was called Rus. Perm. *мeдвeжья лaпa* [“bear’s paw”] [57]. Turk. name *ayı ardıcı* [“bear’s juniper”] was given to *Juniperus drupacea* having bigger cones than other species [59]. It seems that size was also important for *Arbutus unedo* L., as it is compared with grapes and strawberries, but the nominations could also be influenced by the surface of the fruit, as well as by using them as bear food.

#### Nomination by surface type

Another group which assumingly could be big due to hairy look of bear and many plants, in fact contributed only 7% of the motivations. Here a good example is found in the folk names of *Lycopodium* spp.: in Upper Sorbian, German, Polish, Czech, Slovak, Italian, and Hungarian dialects it was compared with bear’s paws, feet, legs, and hair (as well as more general words as ‘herb’ and ‘(club) moss’ in Estonian and German [45, 58, 60–76];. *Lycopodium* was called *björnmåssa* [“bear moss”] in a Swedish source from 1694 [77], which is probably a translation from a German source.

*Equisetum arvense* in Hungarian dialects was called *medveszakál(l)a* [“bear’s beard”], *medvefarka* [“bear’s tail”], and *medvebajusz* [“bear’s moustache”] [78].

#### Nomination by form

In this section it is possible to find several subgroups, according to the form of various parts of the plants. This is probably the reason why this group contributes to highest number of both recorded and assumed motivations (17%). The names could be based on the appearance of:

- large leaves of roundish shape (rather often compared to bear paws): Rus. Kostrom. *мeдвeжьи лaпы* [“bear’s paws”], Bel. Smol., Gomel. *мядзвeжaя лaпкa* [“bear’s little paw”], Norw. *bjønnblekker* [“bear’s leaves”], Est. *karukoll* [“bear’s ogre”] *Caltha palustris* L. [57–59, 80], Bulg. *мeчa cтъпкa* [“bear’s step”] *Tussilago farfara* L. [81], Eng. *bear’s foot Alchemilla vulgaris* L. [45].
- spherical organs of plants (e.g. inflorescences), sometimes with thorns and prickles; could be compared with bear’s head or ear: Kaz. *ayïwbastiken* [“bear’s head”] *Cirsium* sp. [6], Est. *karuohakad*, *karused uhakad*, *karuuhak*, *karuohtjas*, *karuõhakas* [“bear’s thistle”, “bear’s thorn”] *Cirsium arvense* (L.) Scop. [58], Norw. *bjønnehatt* [“bear’s hat”] *Cirsium heterophyllum* L. [80], Rus. Psk. *мeдвeжник бoлoтный* [“swamp bear-plant”] *Cirsium oleraceum* Scop. [57]. Estonian name *karune ohak* for *Carlina vulgaris* L. literally means “bear thistle” [58]. There was also a small group of names for *Centaurea* spp.: Est. *karukellad* [“bear’s bells”] [58] and Rus. *мeдвeдник* [“bear’s plant”], *мeдвeжья лaпa* [“bear’s paw”] [75], Turk. *ayıkulaği* [“bear’s ear”] *Arctotis* sp.; *Aster amellus* [6]. Names of mushrooms with untypical shape, for example, Liv. *okš-šõrməz* [“bear’s septum”] *Gyromitra esculenta* (Pers. ex Pers.) Fr. [81] could also be put here.
  This type of nomination still stays productive; it shows, for example, in Rus. Novosib. *мeдвeжья лaпa* [“bear’s paw”] for some species of cacti; at that, the phytonym has obviously been derived recently.
- Plants with small flowers pressed in bunches, looking fluffy (often compared with ears or paws): Turk. *ayıkulaği* [“bear’s ear”] *Glycyrrhiza glabra*, Tat. *ayu bašï* [“bear’s head”] *Trifolium* sp*.* [6], Rus. Psk. *мeдвeжьe уxo* [“bear’s ear”] *Verbascum nigrum* L. [57], Fin. *karhunkukka* [“bear’s flower”], *karhunruoho* [“bear’s grass”] *Achillea millefolium* L. [82].

As the reason for nomination was seldom indicated explicitly in sources, it seems sound to draw typological arguments. For example, a number of names for *Antennaria dioica* (L.) Gaertn. – Germ. *Bärentatze*, *Bärenpratze*, Cz. *medvědí tlapičky*, Sloven. *medvedove tačice*, Est. *karukäpp*, all literally meaning “bear’s paw(s)” [58, 65, 83, 84], − finds a typological parallel in Rus. *кoшaчьи лaпки*, Germ. *Katzenpfoetle* [“cat’s paws”]. This sub-group may also be enlarged by names for *Clavaria* spp., compared in German, Slovenian, Bulgarian, and Serbo-Croat dialects with bear’s paws, and in Slovenian also with bear’s mane [65, 84, 85].

#### Nomination by toxicity/inedibility for humans/lower status in comparison with cultural analogues

The toxicity along with lower status contribute in sum only 5% of records. Plants with poisonous or just inedible fruits often have the word ‘bear’ as the first element and the word ‘berry’ (or a name of some specific berry plant) as the second, as in Swedish, Norwegian, Bulgarian, Russian, Lithuanian, Estonian, and Finnish names for *Paris quadrifolia* L. [15, 55, 57, 58, 80, 82, 86–88]. The same may be found in many other plants names: for instance, *Crataegus* spp. was compared with apples in Bulgarian, and pears in Slovenian and Italian [84, 86, 89, 90]; *Paeonia* spp. was compared with roses in Turkish [91–94]; *Ribes* spp. was compared with grapes in Italian and Serbo-Croat [84, 90, 91], *Corylus colurna* L. – with hazelnut in Albanian [95], *Lathyrus sylvestris* and *Vicia sepium* L. was compared with peas, in Lithuanian [87, 96], *Oxalis acetosella* was compared with sorrel in Hungarian [97, 98] – in all these cases the abovementioned species have the ‘bear’ prefix. The scornful, negative character of nomination is confirmed by the fact that many of the plants analysed received other folk names with ‘dog’, ‘wolf’, ‘pig’, ‘snake’, etc. as the first component. To step beyond the scope of zoological code, the plants supposed to be unpleasant, harmful, or “not real” were also often named in ethnic terms, specific for each nominating language.

Nevertheless, as bear is taken as the dominating European animal, in Russian dialects (Novgorod, Tver, Vologda, Olonets) the bear’s name was used for nomination of *Boletus edulis* as the best mushroom, the mushroom *par excellence* [57]. The same can be said about Hungarian folk names for *B. edulis* and other *Boletus* spp. in Transylvania [99, 100], and others; see the Table 1, though there is no information on motivations for corresponding cases in Turkish.

#### Nomination by place

Perhaps here “bear nomination” was a particular, narrower case of “wild nomination”. The location in the wild is a “background feature” of the plants, growing far from the human dwelling, in the places where animals live, which serves as a basis for metaphorical alikening of a plant and an animal. Here it seems appropriate to consider a number of names for the fungus *Lycoperdon* spp. In this case, the ability to produce a cloud of spores caused a set of second components of phytonyms, connecting them to bathing, smoking, etc.: Germ. *Bärenfurz* [“bear’s fart”], Bulg. *мeчкинь пуфeш* [“bear’s puff”], Rus. Middle Ob *мeдвeжий дым* [“bear’s smoke”], *мeдвeжий тaбaк* [“bear’s tobacco”], Rus. Vlad. *мeдвeжьи бaни* [“bear’s baths”], Rus. Perm. *мeдвeжьe куpeвo* [“bear’s tobacco”] [65, 86, 101–103]. It is noteworthy that the same fungus was known as Fr. *vesse-de-loup* [“wolf’s fart”], which is the literal translation from Greek. Here we see again, how a bear and a wolf, being similar in folk ideas, could be interchangeable also in folk plant nomenclature. Another example is *Lychnis chalcedonica* L. which has names not only Rus. Ob *мeдвeжьe мылo* [“bear’s soap”], but also Rus. Ob *coбaчьe мылo*, [“dogs’s soap”] [101], Rus. Samar. *кукушкинo мылo* [“cuckoo’s soap”] as well a number of other names [12]. Although this motivation can be perceived as the background in many more cases, especially in case of several possible motivations, it has explicitly contributed to only 2% all records.

#### Nomination by usage for food in bears

Bears are typically omnivorous animals, their diet includes succulent shoots and leaves, fruits, insects, and meat [104], although recent studies have shown that carnivory is positively correlated with latitude among omnivorous mammals [105]. Omnivority of the bear gave it in many cultures the attribute of medicine animal, knowing all the plants and foods in general. As bears were often believed to have supernatural powers (due to their size and long hibernation period) people observed with great attention bear’s way and the way they foraged. We can thus assume that a large proportion of bear names in plants referred to their diet. The literature on bear ecology gave us dozens of bear food plants, and some of them had bear-related names in some languages. The main example of such plants is hogweed (the genus *Heracleum*) reported as one of the main spring foods of the bear from many countries, e.g. the USA, China, Japan, and Poland, e.g. ([106–109], Tomasz Kozica – pers. comm.). Also “bear’s garlic”, *Allium ursinum*, was reported as important bear food in Croatia [110]. There was also an evidence from Mr. Sándor Tímár (Eastern Carpathians) of bears eating *Allium ursinum* and *A. victorialis* (Hung. *vadfokhagyma*, wild garlic), though the plant was not named after bear in this area: “The bear does not eat anything during winter, he licks his paws, and licks so much that by spring they are white. And then he eats first from that plant (wild garlic), in order to clean his stomach from the “deposits”. He is such a clever animal. He searches for what he has to eat after the winter sleep”.^1^ Bears eat a large diversity of wild fruits so it is not surprising that some of them got the names of bear berries, though it is probably impossible to say if it was because they were main fruits eaten by bears or rather those fruits which are less eaten by humans, left for the bears, like *Arctostaphylos uva-ursi*. Bears have also been observed using plants for self-medication, so some of the plants which are not typical bear food or do not resemble bears in any way may have acquired their names from incidents of humans observing a bear using this plant as medicine. This was the case with *Ligusticum porter* which was observed as being sought after by bears and was regarded as bear medicine by Native Americans [111].

It is possible to assume that some plants – their fruits, stalks or rhizomes – were eaten by bears, though dialect dictionaries seldom give explanations, and we do not always know the folk ideas behind this or that nomination. Sw. *björnbär* [“bear berries”] for *Vaccinium myrtillus* L. [15], Cz. *medvědice* [“bear’s plant”] for *Rubus caesius* L. [72], Rus. *мeдвeжьи ягoды* [“bear’s berries”] for *Rubus idaeus* L. [57] seem rather reliable. *Arctostaphylos alpina* (L.) Spreng was also known as “bear berries” in many languages, such as N.S. *guovžžamuorji*, Sw. *björnbär* and Norw. *bjønnbær* (recorded already in 1766 by Gunnerus [112]) [15].

However, not all scholars agreed that there was a necessary connection with the *bear*- prefix and the berries consumed by the animal. Many different kinds of berries were named *björnbär* locally in northern Scandinavia. Black shining fruit varieties of *Vaccinium myrtillus* L. and *Empetrum hermaphroditum* Hagerup were for instance known as *björnbär* “bear berries” in northern Sweden. But normal bluish-coloured fruits, although being eaten by bears, were not called “bear berries” (cf. [37]). However, in some areas the normal-coloured berries were also known as “bear berries”, and known to be eaten by bears as well. Swedish plant name scholar and linguist Karl-Hampus Dahlstedt [15], who studied the north-Scandinavian berry-names in particular, stressed that the naming motif sometimes could be pejorative, for instance for *Paris quadrifolia* and maybe, but not necessary, for *Vaccinium oxycoccos*. Dahlstedt concluded that it was not easy to find one explanation for why many different kinds of berries were known as “bear berries” in northern Scandinavia [15]. Why *Rubus caesius* was called *karuu-marja* “bear-berry” in Votic is not clear either [113].

Another example of a bear food plant is *Cicerbita alpina* (L.) Wallr., which was known as *björnmat* [“bear food”] in Dalecarlia, *björngräs* [“bear grass”] and *björnkål* [“bear cabbage”] in Lapland, as well as *bjønnturt* [“bear plant”] and *bjønnmat* [“bear food”] in Norway. The plant was well-known as appreciated by bears among the peasantry in northern Scandinavia [114]. The same may be assumed about It. *pan d’ors(o)* [“bear’s bread”] *Sorbus aria* L. [74, 115], and Hung. *medvesaláta* [“bear’s lettuce”] which was a name for some woodland fringe tall herbs (*Cirsium erisithales*, *C. oleraceum*, *C. rivulare*, *Carduus personatus*) in the Eastern Carpathians.

This motivation group contributed 7% to all records and the list of plants assumingly motivated by bear food contains 66 plant taxa, among which are the taxa with several possible motivations. Very limited list of taxa detected as bear food in [105], however, only partially overlap with our extended list, as obviously not all bear food was called related to bear (like for example *Populus tremula* L.). Yet for example *Aegopodium podagraria* L., which has unknown motivation, or *Taraxacum* spp., *Tussilago farfara* L. and *Trifolium* spp. assumingly motivated by the form in our sources, or *Urtica dioica* L. motivated by surface have been detected as bear food in [105], which can indicate possible earlier motivation that was later over interpreted.

#### Nomination by folklore motif or belief

This motivation group is not numerous (5%). Rus. Perm. *мeдвeжий тaбaк* [“bear’s tobacco”] *Lycoperdon* was based on the belief that “a bear, to exterminate fleas, rolls about the clearing dotted with puff-balls” [102].

Another case is presented by folk nominations of *Polytrichum commune*. It was called Rus. Orl. *мeдвeдь* [“bear”], Germ. *Bärenmoos*, Est. *karusammal*, Izh. *karunsammõl*, Sw. *björnmossa* (first recorded in 1638), Norw. *bjørnemose*, Liv. *okš-šōmal*, Vot. *karasamma* [“bear’s moss”], Lith. *meškakūšis* [“bear’s penis”] [11, 56, 65, 80, 81, 87, 113, 116].

Slavonic sources did not say anything about the reasons for naming, Finno-Ugrian researchers surmise form nomination type. Really, one could suppose that thick, densely growing small stalks were compared with bear fur (see *Lycopodium* spp. above). But there were several interpretations of this specific name on Nordic material, all of them as good as the others [17, 80] admitted it was not easy to interpret the name: it could be explained by the fact that the moss turned red-brown and could remind one of a bear skin, but more probable, he said, was the connection with the belief that the bears used it in their winter-home. Also the moss *Rhacomitrium lanuginosum* was, on the same reason, named “bear moss” in Norwegian [80].

#### (Partial) translation from Latin

A very important phenomenon mentioned in the beginning of the article is Latin loan translations (calques) in national phyto-taxonomies contributes 15% to all records. The most remarkable cases seem to be *Arctostaphylos* spp., especially *Arctostaphylos uva-ursi* (L.) Spreng.; its names (and over 80 of these have been recorded) have as their inner form “bear’s grapes” or “bear’s berries” in Albanian, Bulgarian, Czech, Danish, English, Estonian, French, German, Italian, Hungarian, Polish, Russian, Serbo-Croat, Slovenian, Turkish, and Ukrainian.

Of course, there is no guarantee that some folk names might not appear independently of learned ones, as various second components are also possible; but the greater the number of semantically identical plant names we find all over Europe, the greater the chances that they are the result of borrowing, as seems to be the case with *Allium ursinum*, called “bear’s onion” or “bear’s garlic” in Italian, German, Lithuanian, Russian, Ukrainian, Polish, Slovenian, Serbo-Croat, Bulgarian, Hungarian, Romanian, Albanian, Estonian, Turkish, Azeri, Bashkir, and Kirghiz. Böhling [117] discussed why *Allium ursinum* had been referred to as “bear’s onion” already by the ancient Greeks. He suggested that bear in the plant epithet referred to Ursa Major (Big Dipper), − the constellation which could be seen in northern skies (other *Allium* species occurred in southern Europe, while *A. ursinum* was one of the most northerly distributed species of onions). His hypothesis is not convincing. First of all, for typological reasons, there do not seem to be any plants named after stars in any of the Germanic or Slavonic languages; the abovementioned motivation by bear food or lower status seems much more logical.

William T. Stearn [118] analysed the means of coining the medieval Latin name *branca ursina* [“bear’s paw”] for *Acanthus mollis* –someone “noted a resemblance between a floral bract of *Acanthus mollis* and a bear’s clawed paw” – and then transferred it (first in France) to *Heracleum sphondylium* which “has large divided leaves somewhat like those of *Acanthus mollis*” [118, 119]. The name *branc* (*branque*, *branche*) *ursine Acanthus mollis*, evidently, provoked the French form *patte d’ours* [2].

It seems that the model turned out to be rather productive, as numerous names for both plants, mostly translated as “bear’s paw” (but also ‘foot’, ‘palm’, ‘claw’, ‘nail’, ‘finger’, etc.), are spread all over Europe – in Upper Sorbian, Slovak, Polish, Czech, Ruthenian (in Vojvodina), Serbian, Croat, Slovenian, Bulgarian, Russian, Romanian, French, Hungarian, English, German, Swedish, Danish, Dutch, North Saami, Votic, Tatar, Chuvash, Azeri, and Bashkir [6, 16, 45, 61–63, 65, 68, 84, 86, 87, 89, 113, 116, 120–129]. The model might also concern other *Heracleum* species; a larger group was made by folk names for *Heracleum sphondylium* L., mostly in Northern Europe.

In these cases we may suggest that first corresponding phytonyms penetrated to Indo-European languages, and later – very likely, through their mediation – to Finno-Ugrian and Turkic ones.

A noteworthy unity of nominations was demonstrated by a number of names for *Primula auricula* L.: Germ. *Bärenohr* [65], S.-C. *мeдвиђe уxo* [84], Cz. *medvědí ouško* [60, 61], Eng. *bear’s ears*, *bazier*, *ba(i)sier* [45], Rus. *мeдвeжьe ушкo* [116], It. *urie/orie d’ours*, *orecchio d’orso, recchietta d’urss* [74], Fr. *oreille d’ours* [2], Ukr. *medved’ače uxo* [130], Bulg. *мeчo уxo* [86]. The semantically analogous Turkish name *ayı kulağı* referred to *Primula auriculata*, *P. elatior* subsp. *pseudoelatior*, *P. longipes*, *P. veris* subsp. *columnae* and *P. veris* subsp. *macrocalyx* [131]. All of them have the inner form “bear’s ear(s)” – thanks to loan translation from Latin. It had a pre-Linnaean name *Auricula ursi* (because of its leaves which seem to be very much shaped as bear ears), which has been translated into many languages, and rendered in the medicinal and botanical literature, for instance in Germ. *Berenohrlein* [132], Dan. *bjørnsøre* [133], Sw. *björnöron* [134, 135].

#### Several nomination types

As it was said above, there are often reasons to assume the existence of more than one basis for nomination, and this group constituted 22% of all motivations. Thus, Rus. Perm *мeдвeжий гpиб* [“bear’s mushroom”] *Sarcodon imbricatus* (L.) P/Karst. was explained by respondents reasoning from its bitter taste; the researcher preferred to mention the size of the mushroom (reaching 30 cm), as well as dark and velvety colouring of the cap [102]. But it also seems important to note that the species has greyish brittle teeth instead of gills on hymenophore which could cause comparison with bear’s head or ear in Bulgarian, Czech, and Belorussian [61, 79, 86]. We have already discussed the case of *Heracleum* species, combining language borrowing and motivation by shape; at the same time, the plants of *Heracleum* genus are used by bears for food. The names for *Allium ursinum* also seem to combine bear-food motivation and loan translation.

#### Motivation unknown

Folk nomination is sometimes so doubtful and dubious that the question of motivation may remain unsolved, as it is, for example, for Alb. *bar i ariut* [“bear grass”] *Erica herbacea* L. [136], and in many other cases. Unfortunately, dialect dictionaries, while recording plant names, seldom give the motivation. This was the case in 13% of all records.

## Discussion and conclusions

**Fig. 4:**
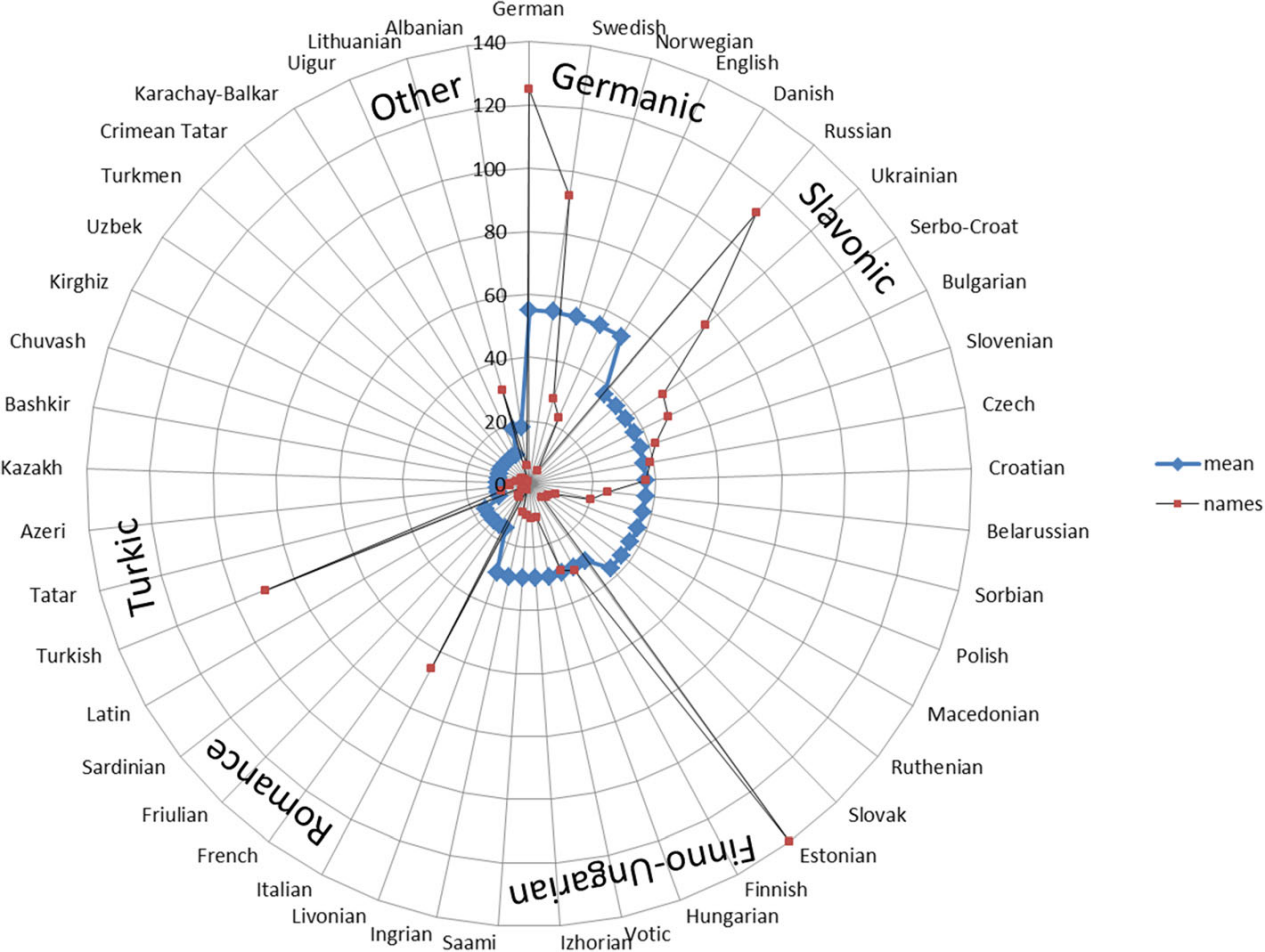
Relation between language groups and the number of recorded bear-phytonyms. names – the number of recorded bear-names. mean – the mean number of names within the language group

The material presented gives wide opportunities for linguo-geographical and ethno-cultural observations and studies. Bear phytonyms were quite widespread in some countries/languages such as Estonian (140), German (125), Russian (122), Swedish (92) and rather rare in the others – Macedonian and Ruthenian (7), Albanian and Slovak (6), Danish (5), Bashkir (4), Chuvash (3), Livonian (2) (these numbers are not absolute, as it is not always easy to differentiate phonetical variants and different phytonyms). This fact can hardly be explained only by environment differences (like for example speakers of Estonian and Livonian inhabited the same ecological niche) nor to linguistic peculiarities (as for example there are bear-rich and bear-poor languages in all main language groups) and needs deeper cultural anthropological studies combined with folklore and ethnology. A very interesting situation was observed in the Turkic languages. There are 93 recorded Turkish bear names, while the other members of the language group demonstrate a much weaker interest in this kind of nomination: 7 (in Tatar), 6 (Kazakh), 5 (Kirghiz), 2 (Turkmen and Uzbek), and 1 (in Gagauz, Uyghur, and Karachay-Balkar) – and even here, around half of these are Latin calques. However, this may, at least to certain extent, be related to the amount of the historical ethnolinguistic research done on the selected languages. Nevertheless, a rough approximation based on the mean number of names in all languages belonging to a language group allowed to list the language groups according to the bear-richness: Germanic, Slavonic, Finno-Ungarian, Romance, Other (Albanian and Lithuanian) and Turkic (Fig. 4). However, such approximation should not be taken too literary, as much depended on the presence of small languages and the languages we researched do not cover all languages of the groups.

From structural point of view, we may conclude that bear-names might be created according to several models: 1) bear/she-bear, 2) bear + a body part of the bear, 3) bear + a part of the plant, 4) bear + a plant name, 5) bear + a plant group name, 6) bear + an object name.

On the one hand, some plants are “champions” in bear-nomination, while others have only one or two bear names. On the other hand, some features – form and/or surface, in our case – cause a very rich pool of names, while such features as colour seem to provoke rather few associations with bear.

Some names recorded in a certain tradition may be used for explanation of dark spots in another one. For example, Serbo-Croat, Hungarian, and Turkish bear-names for *Boletus* spp., having no explanations inside these traditions, may be ascribed to status nomination by analogy with Russian folk names. The case of *Polytrichum commune*, analysed above, allows us to surmise not only form nomination in Russian, Estonian, etc. (based on Nordic explanations), but also borrowing from the Scandinavian languages, as all the names in the pool are recorded in the Northern regions of Europe.

The database we assemble is also valuable for comparative and typological studies. For example, it shows correspondence of names in Latin and in other languages and demonstrates the deep influence of Latin natural-science scholarship upon European culture. Comparing names in neighbouring cultures (e.g. Northern Russian and Estonian; Baltic and Nordic) could become a good basis for studying the question of the borrowing of plant names.

## Geographical marks

Gomel.: Gomelj province, Belarus
Kostrom.: Kostroma province, Russia
Middle Ob: middle part of the river Ob
Novosib.: Novosibirsk area
Orl.: Orel province, Russia
Perm.: Perm province, Russia
Psk.: Pskov province, Russia
Smol.: Smolensk province, Russia
Tobol.: Tobolsk region, Russia
Vlad.: Vladimir province, Russia

## Languages

Alb.: Albanian
Az.: Azeri
Bash.: Bashkir
Bel.: Belarusian
Bulg.: Bulgarian
Chuv.: Chuvash
Cr.: Croat
Cr.-Tat.: Crimean Tatar
Cz.: Czech
Dan.: Danish
Eng.: English
Est.: Estonian
Fin.: Finnish
Fr.: French
Germ.: German
Gr.: Greek
Hung.: Hungarian
I.F.: Ingrian Finnish
It.: Italian
Izh.: Izhorian
K.-B.: Karachay-Balkar
Kaz.: Kazakh
Kirg.: Kirghiz
Lat.: Latin
Lith.: Lithuanian
Liv.: Livonian
N.S.: North Saami
Norw.: Norwegian
Pol.: Polish
Rus.: Russian
Ruth.: Ruthenian
S.-C.: Serbo-Croat
Serb.: Serbian
Slov.: Slovak
Sloven.: Slovenian
Sorb.: Sorbian
Sw.: Swedish
Tat.: Tatar
Turk.: Turkish
Uyg.: Uyghur
Ukr.: Ukrainian
Uz.: Uzbek
Vot.: Votic

## Historical parishes in Estonia

Äks: Äksi
Amb: Ambla
Aud: Audru
Ha: Harjumaa
Hää: Häädemeeste
Hag: Hageri
Han: Hanila
Har: Hargla
Hel: Helme
HJn: Harju-Jaani
Hlj: Haljala
Hls: Halliste
Iis: Iisaku
JJn: Järva-Jaani
JMd: Järva-Madise
Jõe: Jõelähtme
Jõh: Jõhvi
Jür: Jüri
Juu: Juuru
Kaa: Kaarma
Kad: Kadrina
Käi: Käina
Kam: Kambja
Kan: Kanepi
Kei: Keila
Khk: Kihelkonna
Kod: Kodavere
Kos: Kose
Krl: Karula
Kse: Karuse
Ksi: Kursi
Kul: Kullamaa
Kuu: Kuusalu
Lai: Laiuse
Lih: Lihula
LNg: Lääne-Nigula
Lüg: Lüganuse
Mar: Martna
Mih: Mihkli
MMg: Maarja-Magdaleena
Mus: Mustjala
Nis: Nissi
Noa: Noarootsi
Nrv: Narva
Ote: Otepää
Pal: Palamuse
Pär: Pärnu
Phl: Pühalepa
PJg: Pärnu-Jaagupi
Plt: Põltsamaa
Puh: Puhja
Rap: Rapla
Rid: Ridala
Rõu: Rõuge
Saa: Saarde
Se: Setumaa
SJn: Suure-Jaani
Tln: Tallinn
Tõs: Tõstamaa
Trm: Torma
Trt: Tartu
Trv: Tarvastu
Tür: Türi
Vai: Vaivara
Var: Varbla
Vig: Vigala
Vil: Viljandi
Vll: Valjala
Võn: Võnnu

## Footnotes

1 Special thanks to Dániel Babai for this fieldwork evidence.
